# Rational Design of Broad‐Spectrum Anti‐Enteroviral Molecular Glues Targeting Enteroviral RNAi Suppressors

**DOI:** 10.1002/advs.75317

**Published:** 2026-04-16

**Authors:** Yuan Fang, Xiong Xie, Huidi Fan, Botao Wu, An Wang, Wenhao Dai, Zezhong Liu, Jian Li, Huoyan Tong, Jianan Li, Yujie Ren, Jinlin Wang, Xi Zhou, Hong Liu

**Affiliations:** ^1^ State Key Laboratory of Virology and Biosafety Wuhan Institute of Virology Chinese Academy of Sciences Wuhan Hubei China; ^2^ University of Chinese Academy of Sciences Beijing China; ^3^ State Key Laboratory of Drug Research Shanghai Institute of Materia Medica Chinese Academy of Sciences Shanghai China; ^4^ School of Pharmaceutical Science and Technology Hangzhou Institute for Advanced Study University of Chinese Academy of Sciences Hangzhou China; ^5^ Department of Pharmacology & the Key Laboratory of Smart Drug Delivery Ministry of Education School of Pharmacy Fudan University Shanghai China

**Keywords:** broad‐spectrum anti‐enterovirus, molecular glues, rational design, viral suppressor of RNAi (VSR)

## Abstract

Rational design of molecular glues (MGs) remains challenging, as most have been discovered serendipitously and have found limited application in antivirals. Previously, we identified the enteroviral 3A protein as a viral suppressor of RNAi (VSR) that functions through homodimerization to inhibit the antiviral RNA interference (RNAi) pathway. Herein, capitalizing on this homodimerization mechanism, we rationally designed 3A‐targeting broad‐spectrum anti‐enteroviral molecular glues targeting the dimeric interface to induce dysfunctional dimerization. The optimal compound, VTP‐32, exhibited good binding affinity with 3A (K*_D_* = 0.29 µm), potent and pan‐enterovirus (groups A, B, D) antiviral effects (EC_50_ = 0.21–0.92 µm), and good safety (CC_50_ > 500 µm). VTP‐32 treatment (20 mg/kg) could effectively reduce viral load, alleviate clinical symptoms, and improve survival in EV‐A71‐infected mouse models. Mechanistic studies revealed that VTP‐32 stabilizes 3A protein into an abnormal dimer, promotes viral siRNA generation, and ultimately leads to RNAi‐mediated viral genome degradation. Overall, this study provides a promising countermeasure against enteroviral diseases and a rational design strategy for developing antiviral molecular glues.

## Graphical abstract

By leveraging this homodimerization mechanism, molecular glues were rationally designed to induce dysfunctional 3A dimerization, thereby restoring antiviral RNAi. The optimal molecular glue, VTP‐32, demonstrated potent and pan‐enterovirus (groups A, B, D) antiviral effects. VTP‐32 significantly reduced viral load and improved survival rates in an EV‐A71‐infected mouse model. These findings highlight a promising strategy for developing antiviral molecular glues.

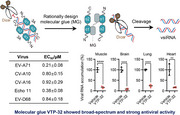

## 1 Introduction

Traditional antiviral agents typically function by binding to functional active sites of virus infection‐related factors, such as well‐established protease inhibitors and polymerase inhibitors. This kind of antiviral approach often falls into unsatisfactory efficacy outcomes due to rapid drug‐resistance mutation, side effects, or a lack of broad‐spectrum efficacy, highlighting the urgent need for novel strategies to effectively prevent and treat both existing and newly emerging viral infectious diseases [1]. Proximity induction is an emerging concept that, unlike conventional occupation‐driven strategies, achieves pharmacological efficacy by modulating protein‐protein interactions in spatial proximity. Notable examples, including PROTAC and molecular glues, have gained significant attention in recent years [2]. This innovative concept has already been applied in the antiviral field [3], as exemplified by several reported viral PROTAC degraders that exhibit antiviral activity targeting HCV NS3/4A [4], SARS‐CoV‐2 M^pro^ [5], and HIV‐1 Nef [6]. However, molecular glues have been rarely reported in the field of virology [7, 8, 9, 10, 11]. Molecular glues are capable of inducing novel protein‐protein interactions or stabilizing existing ones, thereby facilitating protein degradation, complex stabilization, interaction network modulation, and transporter inhibition [12, 13]. Currently, most molecular glues have been discovered serendipitously, and the rational design of molecular glues remains a major challenge in drug discovery due to the limited elucidation of design principles and modes of action. Therefore, the development of antiviral molecular glues based on a rational drug design strategy is highly significant.

During virus infection, RNA interference has been demonstrated to play a crucial role in antiviral immune responses in eukaryotes [14, 15]. Double‐stranded RNA (dsRNA) produced during viral RNA replication is processed by the endogenous ribonuclease Dicer into 22±1 nucleotide virus‐derived siRNAs (vsiRNAs), which are then loaded into the RNA‐induced silencing complex (RISC). This ultimately leads to the degradation of viral RNA by Argonaute (AGO) proteins, thereby inhibiting viral infection [16, 17]. However, viruses have evolved mechanisms to evade this immune response by encoding viral suppressors of RNAi (VSRs) that interact with key components of the RNAi pathway [16, 17, 18]. Many viruses encode VSR proteins, including influenza virus, flaviviruses, enteroviruses, alphaviruses, coronaviruses, and Ebola virus [19, 20, 21, 22, 23, 24, 25]. The presence of VSRs is a key reason why vsiRNAs have been difficult to detect in infected mammalian somatic cell lines. Nevertheless, recent advances have enabled the unlocking of RNAi‐mediated antiviral immunity through strategies involving mutant viruses [20, 21, 26], chemical agents [27, 28], or viral vaccines [29]. These approaches have significantly unleashed the antiviral potential of RNAi in mammals, demonstrating strong antiviral activity. This highlights the great therapeutic potential of targeting VSRs to activate cellular RNAi‐based antiviral immunity.

Enterovirus A71 (EV‐A71) has been the main causative pathogen of HFMD among enteroviruses for a long time. However, with the emergence of the EV‐A71 vaccine, the major causative pathogens of HFMD shifted toward other group A enteroviruses, including coxsackieviruses CV‐A16, CV‐A4, CV‐A6, CV‐A8, and CV‐A10, and so on [30]. In addition, Enterovirus‐B, such as CV‐B3, CV‐B5, echovirus 11 (Echo 11), and Enterovirus‐D, especially EV‐D68, can also cause severe human diseases, including myocarditis, aseptic meningitis, severe pneumonia, respiratory failure, and more [31, 32, 33]. Therefore, it is of great significance to develop broad‐spectrum anti‐enteroviral drugs. Our previous works have found that some VSRs employ dimerization or oligomerization to execute their RNAi suppression function, including the enterovirus EV‐A71 3A protein [21, 28, 34, 35, 36]. The 3A protein consists of two amphipathic α‐helices, α1 and α2. In the functional form, the α‐helices from two 3A monomers interact to form an antiparallel homodimer, which binds to viral dsRNA to protect it against Dicer cleavage, thereby blocking the antiviral RNAi response [21]. These studies have demonstrated that mutations at the dimerization interface of the EV‐A71 3A protein, along with the rational design of 3A‐targeting peptide ER‐DRI, can disrupt 3A dimerization and restore the antiviral efficacy of RNAi [21, 27]. Furthermore, the high conservation of the 3A protein across enterovirus groups A, B, C, and D makes it an attractive target for broad‐spectrum anti‐enteroviral drugs (the comparative computational analysis of sequence identity is described below). Therefore, in this study, building upon the homodimerization mechanism and the antiparallel dimeric structure and inspired by the 3A‐targeting peptides—particularly this peptide derived from the α2 helix confers superior antiviral activity compared to that from α1 helix [27]—we hypothesize that it is possible to design a broad‐spectrum anti‐enteroviral molecular glue containing two α2 helix mimics that each binds to the α1 helix of a separate 3A monomer, thereby modulating the formation of a dysfunctional dimer and disrupting the VSR function of 3A.

To achieve the rational design of molecular glues, we adopted a two‐step strategy. Building on the observation that α2‐based peptides exhibit superior antiviral activity compared to α1‐based ones in our previous study [27], we first aimed to design, synthesize, and optimize a cell‐permeable α2 helix peptidomimetic with antiviral activity, then linked two units via a flexible spacer to form the desired molecular glue, which promotes the formation of an antiparallel dysfunctional dimer. Specifically, a core octapeptide EEVRQYCR derived from the α2 helix involved in the dimeric interface was selected as the starting point, and a series of peptidomimetics without a cell‐penetrating peptide (CPP) was designed and synthesized. The antiviral activities of these peptidomimetics were evaluated, and VTP‐30 displayed good antiviral activity against EV‐A71 with an EC_50_ value of 0.98 µm. Then, we dimerized VTP‐30 via a disulfide bond to obtain the designed molecular glue, and systematic peptidomimetic modification was conducted. The optimized homodimer VTP‐32 showed significantly improved anti‐EV‐A71 activity in RD cells with an EC_50_ value of 0.21 µm, and good safety with a CC_50_ value of more than 500 µm. Additionally, VTP‐32 exhibited broad‐spectrum inhibitory activity against various enteroviruses in RD cells, including CV‐A16 (EV‐A), CV‐A10 (EV‐A), Echo 11 (EV‐B), and EV‐D68 (EV‐D). In a mouse model of EV‐A71 infection, treatment with VTP‐32 (20 mg/kg) demonstrated significant antiviral effects with improved survival, alleviated clinical symptoms, and reduced viral loads. Mechanistic exploration using Western blotting with a FITC‐linked probe (FITC‐VTP‐32) and a photoaffinity labeling probe (PAL‐VTP‐32) showed that VTP‐32 could induce the formation of an abnormal dimer (3A–VTP‐32–3A). Furthermore, VTP‐32 treatment promoted viral siRNA production, as detected by Northern blotting, ultimately leading to viral genome degradation, thereby demonstrating potent antiviral activity via a Dicer‐dependent RNAi pathway. This study designed and developed a promising molecular glue with pan‐enterovirus inhibitory effect and potent in vivo antiviral efficacy, which provides a novel insight into antiviral drug development.

## 2 Results

### 2.1 Rational Design of Molecular Glue Targeting 3A Dimerization Interface

**FIGURE 1:**
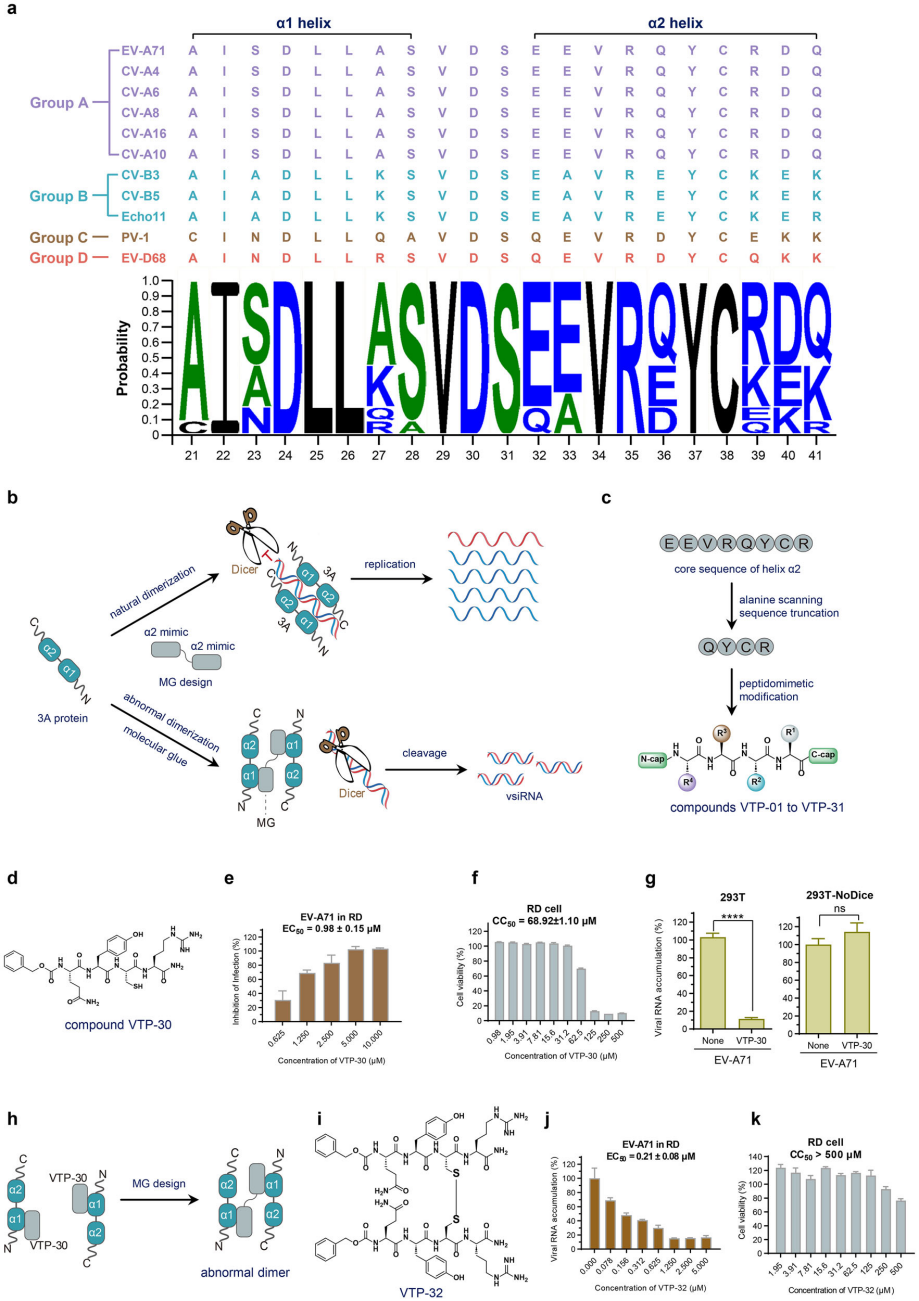
Rational design of 3A‐targeting molecular glues. (a) Comparative analysis of the amino acid sequences of the 3A protein α1 and α2 helices across enteroviruses A, B, C, and D. (b) Schematic diagram of 3A‐targeting molecular glues design. Upon viral infection, the virus‐encoded 3A protein functions as a viral suppressor of RNAi (VSR) by binding to double‐stranded RNA (dsRNA) generated during viral replication. However, the presence of a molecular glue disrupts this mechanism by inducing the formation of a dysfunctional complex. (c) General optimization process for identifying α2 helix mimics. (d) Chemical structure of VTP‐30. (e) VTP‐30 showed good antiviral activity in EV‐A71‐infected RD cells. Effects of increasing concentrations of VTP‐30 on EV‐A71 were determined by CCK‐8 assay, and the absorbance at 450 nm was measured by a microplate reader (Infinite M200PRO). (f) VTP‐30 showed good cellular safety in RD cells. Increasing concentrations of VTP‐30 in DMEM with 2% FBS were added to RD cells for 12 h at 37°C. Cell viability of VTP‐30 was determined by CCK‐8 assay, and the absorbance at 450 nm was measured by a microplate reader (Infinite M200PRO). (g) Antiviral activity of VTP‐30 in 293T and Dicer‐knockout 293T (293T‐NoDice) against EV‐A71 infection. 293T or 293T‐NoDice cells were infected with EV‐A71 and then treated with or without 5 µm VTP‐30 at 1 h.p.i. EV‐A71 genomic RNA levels were determined via qRT‐PCR at 24 h.p.i. (h) Graphical illustration of molecular glue design. (i) Chemical structure of VTP‐32. (j) VTP‐32 showed good antiviral activity in EV‐A71‐infected RD cells. Effects of increasing concentrations of VTP‐32 on EV‐A71 RNA accumulation in RD cells. Viral RNA levels were determined via qRT‐PCR. (k) VTP‐32 showed good cellular safety in RD cells. Increasing concentrations of VTP‐32 in DMEM with 2% FBS were added to RD cells for 12 h at 37°C. Cell viability of VTP‐32 was determined by CCK‐8 assay, and the absorbance at 450 nm was measured by a microplate reader (Infinite M200PRO). Data are means ± SEM. All experiments were independently conducted in triplicate and repeated at least twice with reproducible results. *p*‐value is from a two‐sided unpaired *t*‐test. ns, *p* > 0.05, ^****^, *p* ≤ 0.0001.

We selected several representative enteroviruses from groups A, B, C, and D known to cause severe human diseases and carried out a comparative computational analysis of their 3A protein sequences. The results demonstrated that the 3A protein is conserved, notably the α1 and α2 helices involved in dimerization, which exhibit a high degree of sequence identity (Figure 1a; Figure S1a). Our previous works demonstrated that during natural infection, the enteroviral 3A protein homodimerizes through an interaction in which the α1 and α2 helices of one 3A protein interact with the α2 and α1 helices of another, respectively. The resulting 3A homodimer then binds viral dsRNA, protecting it from cleavage by host Dicer, thereby promoting viral RNA production and replication (Figure 1b) [21]. We further identified a 3A‐targeting peptide, ER‐DRI, which acts as a blocker of 3A homodimerization; however, its delivery requires the use of a cell‐penetrating peptide (CPP) for cellular uptake [27]. Therefore, based on the sequence identity of enterovirus 3A protein and the dimeric interface formed between the α2 and α1 helices, and the superior antiviral activity observed for α2 helix‐derived peptides compared to α1 helix‐based one in our prior work [27], we hypothesize that a broad‐spectrum anti‐enteroviral molecular glue containing two α2 helix mimics can be designed to bind separately to the α1 helices of two 3A monomers, thereby inducing the formation of an abnormal dimer and disrupting the physiological function of 3A (Figure 1b).

To test this hypothesis, we adopted a two‐step strategy. We first tried to identify a cell‐permeable α2 helix peptidomimetic with antiviral activity, and then linked two units via a flexible spacer to form the desired molecular glue, which promotes the formation of an antiparallel dysfunctional dimer. We selected the octapeptide EEVRQYCR (ER) derived from the critical core sequence of the α2 helix involved in the 3A dimerization interface as the starting point for optimizing the α2 helix mimic (Figure 1c). The antiviral activity of octapeptide ER was assessed in EV‐A71‐infected human rhabdomyosarcoma (RD) cells by measuring viral RNA accumulation, to enhance cellular uptake, and a cell‐penetrating peptide (CPP, TAT_47‐57_) was linked to the *N*‐terminus of ER. The results showed that octapeptide ER had a moderate anti‐EV‐A71 activity with an EC_50_ value of 2.43 µm, while the CPP alone showed no inhibition against viral infection (Figure S1b). Next, to further identify the critical amino acid residues in the sequence, alanine scanning was performed, and eight ER variants (ER‐A1 to ER‐A8, Figure S1b) were designed and synthesized. The results indicated that the antiviral effect was decreased to varying degrees. Among them, mutations at the arginine and cysteine residues (EEV **R** QY **CR**) had the most significant impact on antiviral efficacy, with a marked decrease observed when these residues were replaced with alanine (Figure S1b). To investigate the effect of peptide length, we successively truncated ER from the *N*‐ to the *C*‐terminus down to a tetrapeptide, which represents the minimum length required to form a complete α‐helix. These shortened peptides (E2R, VR, RR, and QR, Figure S1c) exhibited comparable or slightly improved antiviral effects compared to ER. Taking into account both antiviral efficacy and peptide size, we finally chose tetrapeptide QYCR for further study.

CPP‐conjugated QYCR (QR) showed a good anti‐EV‐A71 effect with an EC_50_ value of 1.27 µm, but removal of the CPP resulted in loss of antiviral activity at a concentration of 50 µm. Because CPP introduction may decrease proteolytic stability, increase toxicity risk, and weaken drug‐likeness [37], a series of peptidomimetics derived from tetrapeptide QYCR were designed and synthesized without CPP decoration. First, we performed bioisosteric replacement of glutamine (Q), tyrosine (Y), and arginine (R) with hydrophobic residues to increase lipophilicity and cell permeability. Specifically, γ or δ lactam, 4‐fluorophenyl, and ureido were used to replace the side chains amide of Q, 4‐hydroxyphenyl of Y, and guanidino of R, respectively. We successively altered one, two, or three of the proteinogenic residues of QYCR to obtain ten peptidomimetics (VTP‐01 to VTP‐10, Figure S2a). However, these peptidomimetics showed no inhibitory activity against EV‐A71 at 5 µm. Second, we retained the sequence as QYCR and explored terminal capping using different substituted acetyl‐based groups at the *N*‐terminus and substituted amino groups at the *C*‐terminus (VTP‐11 to VTP‐31, Figure S2b). The result demonstrated that appropriate *N*‐ or *C*‐terminus capping groups could moderately enhance the antiviral effect. Among them, compound VTP‐30 (Cbz‐QYCR‐NH_2_), featuring an *N*‐terminal (benzyloxy)carbonyl (Cbz) group and a *C*‐terminal amino (NH_2_) group (Figure 1d), showed complete inhibition against EV‐A71 at a concentration of 5 µm. In summary, small *C*‐caps and flexible *N*‐caps function effectively because such modifications perhaps minimize conformational perturbations in the peptidomimetics, thereby preserving antiviral activity. Furthermore, a serial dilution test was conducted to assess the antiviral activity and cytotoxicity of VTP‐30 in RD cell lines. It was found that VTP‐30 exhibited a similar antiviral effect (EC_50_ = 0.98 µm) to that of QR (CPP‐Linker‐QYCR, EC_50_ = 1.27 µm, Figure 1e; Figure S1c). However, VTP‐30 showed slight toxicity, with a CC_50_ value of 68.92 µm (Figure 1f). Furthermore, the antiviral activity of VTP‐30 is dependent on the RNAi pathway, as evidenced by its significantly reduced effectiveness in Dicer‐knockout human embryonic kidney 293T cells (293T‐NoDice) compared to that in wild‐type 293T (Figure 1g). This suggests that the optimized peptidomimetics retain the RNAi pathway as their antiviral mechanism.

With the optimal α2 helix peptidomimetic VTP‐30 in hand, we next sought to link two VTP‐30s through a flexible linker to construct the designed molecular glues, which were expected to promote the formation of an antiparallel dimer. We connected two VTP‐30 (Cbz‐QY **C** R‐NH_2_) via a disulfide bond formed between their cysteine residues to construct a dimeric molecular glue and designated it as VTP‐32 (Figure 1h,i). To assess whether the increased molecular size after dimerization affected membrane permeability, we tested the cellular uptake of VTP‐32. A fluorophore (fluorescein isothiocyanate, FITC) was conjugated to the *N*‐terminus of VTP‐32 to synthesize a probe FITC‐VTP‐32 (Figure S3a). Obvious fluorescence intensity was observed in FITC‐VTP‐32‐treated cells, indicating that VTP‐32 exhibited good cellular permeability without the need for CPP installation (Figure S3b). We then evaluated the antiviral activity and safety of VTP‐32 and found that VTP‐32 exhibited superior antiviral activity (EC_50_ = 0.21 µm) and lower cytotoxicity (CC_50_> 500 µm) with a higher SI value of 2381, compared to VTP‐30 (EC_50_ = 0.98 µm, CC_50_ = 68.92 µm, SI = 70) (Figure 1e,f, j,k). Subsequently, VTP‐32 was subjected to optimization using different peptidomimetic modification strategies, including sequence reversal, truncation, and terminal capping. First, replacing *L*‐amino acid residues with *D*‐isomers or/and reversing the peptide sequence of VTP‐32 resulted in a significant loss of antiviral activity at a concentration of 5 µm (VTP‐33 to VTP‐35, Figure S3c). Second, attempts to reduce the molecular size by truncation also led to a significant loss of antiviral activity (VTP‐36 to VTP‐39, Figure S3c). Third, we focused on the modification of *N*‐terminal capping group, exploring a series of (benzyloxy)carbonyl group analogues with substitutions of lipophilicity‐increasing moieties, including halogen, alkyl, etc. (VTP‐40 to VTP‐48, Figure S4a). These *N*‐terminus modified peptidomimetics displayed moderate antiviral potency with EC_50_ values ranging from 0.44 to 1.20 µm. However, none exhibited better antiviral activity and safety compared to VTP‐32, which was therefore selected for further antiviral efficacy evaluation and mechanism of action validation (Figure 1j,k; Figure S4a,b).

**FIGURE 2:**
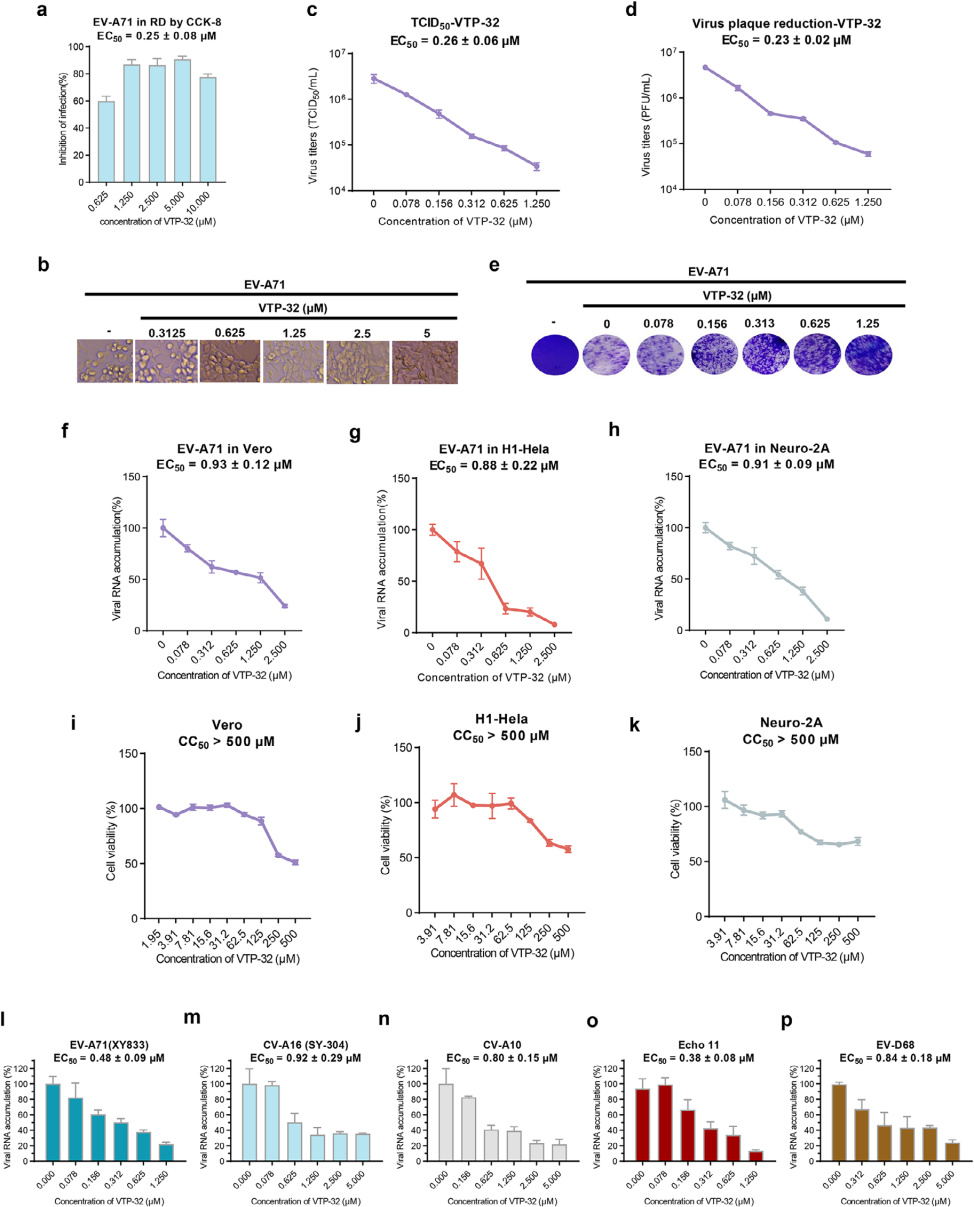
VTP‐32 displayed potent and broad‐spectrum antiviral activity against enteroviruses A, B, and D. (a) VTP‐32 showed good antiviral activity in EV‐A71‐infected RD cells. Effects of increasing concentrations of VTP‐32 on EV‐A71 were determined by CCK‐8 assay, and the absorbance at 450 nm was measured by a microplate reader (Infinite M200PRO). (b) Two‐fold increasing concentrations of VTP‐32 were added into EV‐A71‐infected (MOI = 0.1) RD cells, and the cytopathic effect (CPE) of RD cells was observed using a microscope at 24 h.p.i. (c) Antiviral activity of VTP‐32 was determined using TCID_50_ assay in EV‐A71‐infected RD cells. (d‐e) RD cells were seeded in 24‐well plates, followed by infection of EV‐A71 (MOI = 0.1), and then serially two‐fold diluted VTP‐32 was added. At 4 h.p.i., The supernatants were replaced by fresh DMEM containing 2% low‐melting‐point agarose for 2 days, and cells were fixed with phosphate‐buffered saline (PBS) containing 1% crystal violet and 4% formaldehyde at 4°C for 2 h. (f–h) qRT‐PCR was performed to assess the VTP‐32's antiviral potency, and all of which confirmed the superior antiviral efficacy of VTP‐32. VTP‐32 also exhibited excellent antiviral effects in EV‐A71‐infected Vero (f), H1‐HeLa (g), and Neuro 2A (h). (i–k) VTP‐32 has good safety in corresponding cell lines determined by CCK‐8 assay. (l‐p) Antiviral activity of VTP‐32 against EV‐A71 (XY833) (l), CV‐A16 clinical strain SY304 (m), CV‐A10 (n), Echo 11 (o), and EV‐D68 (p) in RD cells. Effects of increasing concentrations of VTP‐32 on viral RNA accumulation in RD cells (MOI = 0.1). Viral RNA levels were determined via qRT‐PCR. Data are means ± SEM. All experiments were independently conducted in triplicate and repeated at least twice with reproducible results.

To further assess the antiviral potency of VTP‐32, multiple assays were performed in EV‐A71‐infected RD cells, including CCK‐8 assay (Figure 2a), observation of cytopathic effect (Figure 2b), TCID_50_ assay (Figure 2c), and plaque reduction assay (Figure 2d,e). All results consistently confirmed the superior antiviral efficacy of VTP‐32. In a panel of EV‐A71 infection cell lines, including Vero, H1‐HeLa, and Neuro 2A, VTP‐32 exhibited potent antiviral activity, with EC_50_ values of 0.93, 0.88, and 0.91 µm, respectively (Figure 2f–h). VTP‐32 also demonstrated excellent safety across all cell lines tested, with CC_50_ values exceeding 500 µm (Figure 2i–k).

### 2.2 VTP‐32 Exhibits Excellent and Broad‐Spectrum Antiviral Activity

The comparative computational analysis of the 3A protein sequences from several representative disease‐causing enteroviruses (groups A‐D) revealed that the amino acids within the regions designed for molecular glue are conserved among them (Figure 1a). Therefore, we further evaluated the broad‐spectrum anti‐enteroviral activities of VTP‐32, and the results demonstrated potent antiviral activity of VTP‐32 against enterovirus group A, including the clinical strain of EV‐A71 (XY833), CV‐A16, and CV‐A10 in RD cells with EC_50_ values of 0.48, 0.92, and 0.80 µm, respectively (Figure 2l–n). Additionally, although several amino acid differences exist within the 3A‐dimerizing interface of group B and D viruses compared to group A, VTP‐32 still exhibited significant antiviral effect against groups B and D of enteroviruses, with EC_50_ values of 0.38 µm against Echo 11 (group B) and 0.84 µm against EV‐D68 (group D) (Figure 2o,p).

### 2.3 VTP‐32 Functions as a Molecular Glue by Specifically Engaging With the 3A Dimer

**FIGURE 3:**
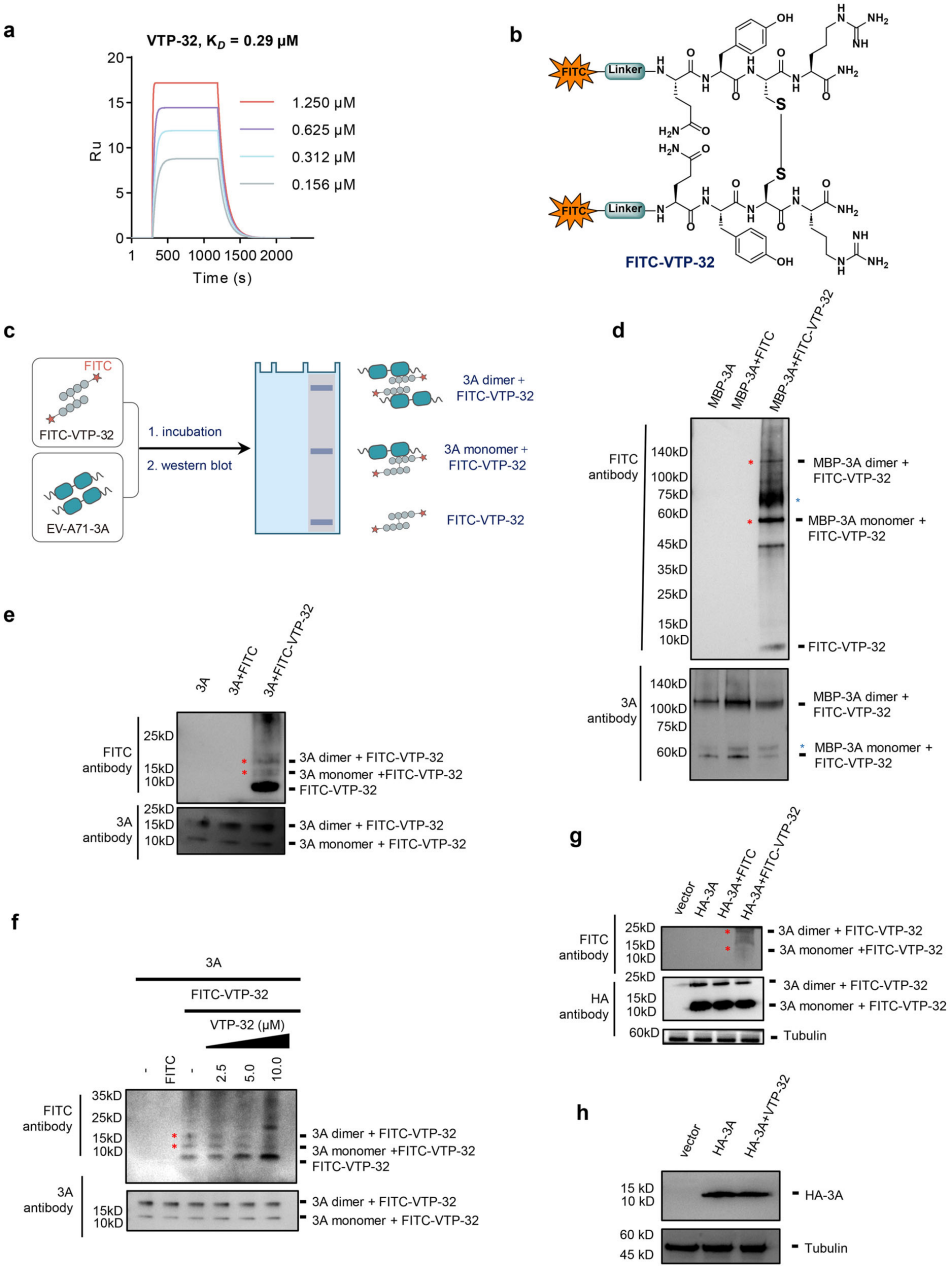
VTP‐32 can bind to the 3A dimer. (a)The surface plasmon resonance assay was performed with 2 mg/mL of the purified MBP‐3A protein incubated with VTP‐32 at the indicated concentrations. (b) Schematic diagram of the VTP‐32 labeled with fluorophore FITC. (c) Schematic diagram of the assay detecting the binding between FITC‐VTP‐32 and 3A: After incubating FITC‐VTP‐32 with EV‐A71 3A protein, a Western blotting was performed to detect the band positions of FITC‐VTP‐32 and the 3A dimer. (d) Incubate MBP‐3A (2 mg/mL) protein with FITC‐VTP‐32 (200 µm) at 4°C overnight, and run a Native‐PAGE gel the next day, followed by Western blotting analysis using FITC‐specific antibody and 3A‐specific antibody, respectively. (e) MBP‐3A (2 mg/mL) is incubated with Factor‐Xa (NEB) at 4°C overnight. Then incubate tag‐free 3A protein with FITC‐VTP‐32 (200 µm) at room temperature for 2 h, and run a Native‐PAGE gel, followed by Western blotting analysis using FITC‐specific antibody and 3A‐specific antibody, respectively. (f) Competitive Binding Assay. Incubate tag‐free 3A protein with tag‐free‐VTP‐32 (200 µm) at 4°C overnight. Then Incubate with FITC‐VTP‐32 (200 µm) at room temperature for 2 h and run a Native‐PAGE gel the next day, followed by Western blotting analysis using FITC‐specific antibody and 3A‐specific antibody, respectively. (g) We transfected 2 µg of pRK‐HA‐3A protein into 293T cells, replaced the medium after 4 h, and treated the cells with FITC‐VTP‐32. Control groups included cells transfected with empty vector, cells transfected with pRK‐HA‐3A only, and cells transfected with pRK‐HA‐3A followed by treatment with FITC alone. After 24 h, protein samples were collected and analyzed by Native PAGE, followed by Western blotting using antibodies against FITC and HA. (h) Detection of the effect of VTP‐32 on the expression level of the 3A protein. 293T cells were transfected with the pRK‐HA‐3A plasmid. The cells were treated with VTP‐32 4 h post‐transfection and were harvested 24 h after transfection. The protein expression level of 3A was then analyzed by SDS‐PAGE and Western blotting.

To prove our initial hypothesis that VTP‐32 functions as a molecular glue, a panel of assays was conducted. First, we confirmed the interaction between VTP‐32 and 3A protein using purified MBP‐tagged 3A in vitro, followed by surface plasmon resonance (SPR) and isothermal titration calorimetry (ITC) assays, and the results showed that VTP‐32 had good binding affinity with EV‐A71 3A with K*_D_* values of 0.29 and 0.24 µm, respectively (Figure 3a; Figure S5a). To further characterize the interaction between them, we labeled VTP‐32 with FITC at its *N*‐terminus and performed Native‐PAGE coupled with Western blotting (Figure 3b). The blot was probed with an anti‐FITC antibody to detect VTP‐32 and an anti‐3A antibody to identify 3A dimer or monomer positions. If FITC‐VTP‐32 binds two 3A monomers to form a new dimer, the anti‐FITC signal should co‐localize with the anti‐3A signal at the dimer position; if it binds the monomer, the signal would align with the 3A monomer, while unbound FITC‐VTP‐32 would migrate lower on the gel (Figure 3c). As shown in Figure 3d, FITC‐VTP‐32 incubated with MBP‐3A produced a clear band at the dimer position, demonstrating specific binding to the MBP‐3A dimer as a molecular glue. Additionally, we also detected the band corresponding to the binding of VTP‐32 to the 3A monomer, which may represent the intermediate state in the formation of abnormal dimers. Moreover, the dual‐binding mode may enhance its interaction efficiency with viral proteins, thereby improving inhibitory activity. In contrast, FITC alone did not produce any signal at the dimer position when incubated with MBP‐3A, confirming that the binding was specific to VTP‐32 and not an artifact of the fluorescent tag (Figure 3d).

To rule out any influence from the MBP tag, we repeated the experiment using untagged 3A and observed that FITC‐VTP‐32 still bound to the 3A dimer (Figure 3e), confirming that the interaction is mediated by 3A itself rather than the MBP moiety. Furthermore, in a competition assay, increasing concentrations of unlabeled VTP‐32 gradually reduced the anti‐FITC signal at the dimer position while the signal for free FITC‐VTP‐32 increased (Figure 3f). This supports the hypothesis that VTP‐32 acts as a “molecular glue”, engaging the 3A into an abnormal dimer. Meanwhile, we overexpressed the 3A protein in 293T cells and treated them with FITC‐VTP‐32. We found that this peptidomimetic could also bind to 3A and facilitate the formation of VTP‐32‐containing 3A dimer under cellular conditions (Figure 3g). For direct validation, we introduced a photo‐crosslinking probe at the *N*‐terminus of VTP‐32, designated as PAL‐VTP‐32. After incubating with MBP‐3A, UV irradiation induced covalent crosslinking, followed by click chemistry to conjugate a rhodamine fluorophore (Figure S5b,c). Native‐PAGE analysis revealed that the rhodamine signal precisely overlapped with the 3A dimer band (Figure S5d), providing additional evidence that VTP‐32 interacts with the 3A dimer and functions as a molecular glue. Finally, to determine whether VTP‐32 affects the expression of the 3A protein, we expressed the pRK‐HA‐3A in 293T cells and treated them concurrently with VTP‐32. Then we examined 3A expression by Western blotting. As shown in Figure 3h, treatment with VTP‐32 did not alter the expression of 3A in cells, nor did it promote 3A protein degradation. Instead, it functions solely as a molecular glue, inducing the formation of an atypical heterodimer. These data demonstrated that VTP‐32 inhibits physiological function by stabilizing pre‐existing protein‐protein interactions, representing a novel therapeutic strategy, which has recently attracted significant attention in drug discovery [38].

### 2.4 The Antiviral Activity of VTP‐32 Is Dependent on the RNAi Pathway

**FIGURE 4:**
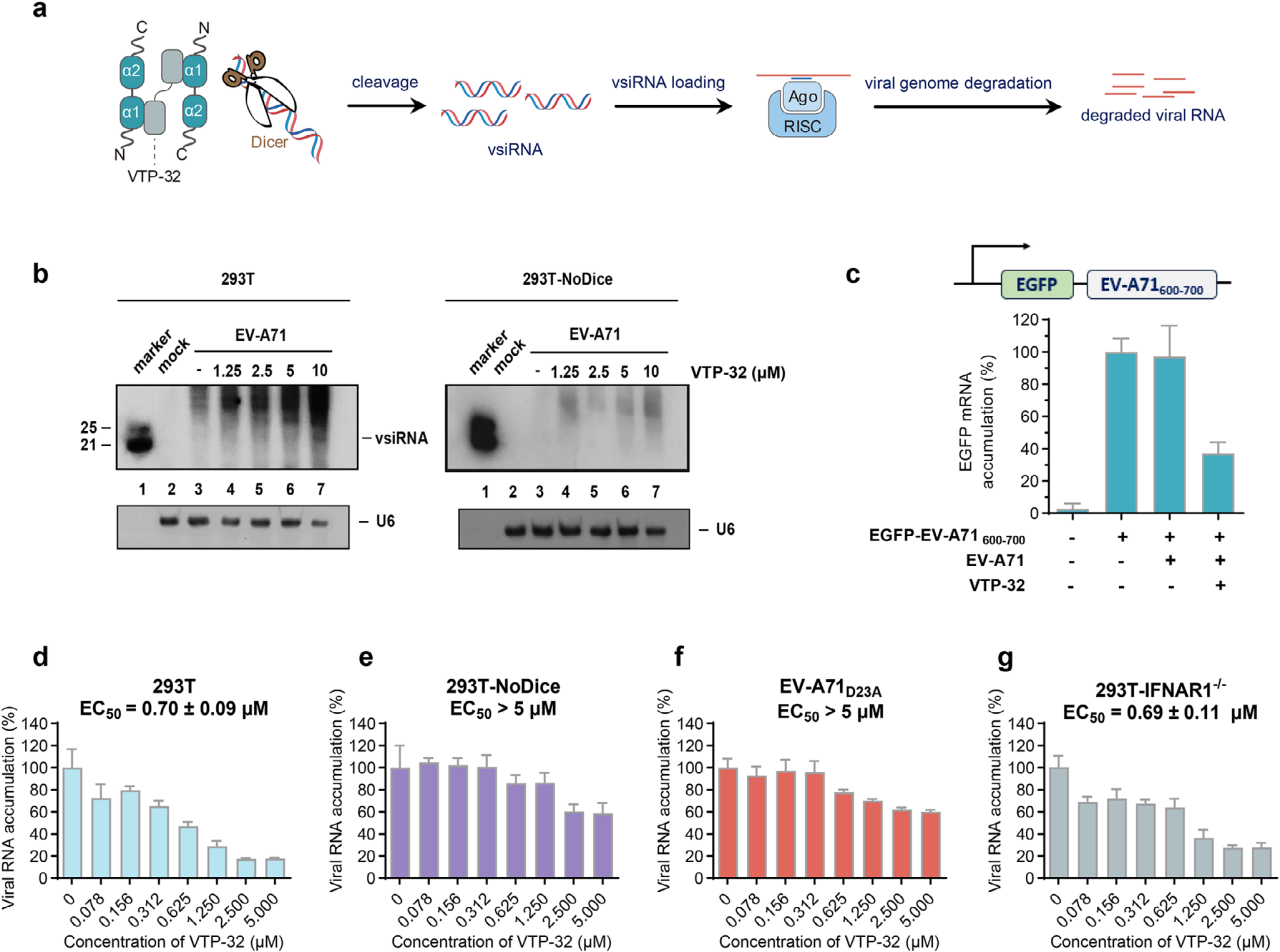
VTP‐32 can unlock the RNAi antiviral immune pathway. (a) A proposed schematic diagram of VTP‐32‐induced RNAi antiviral immunity. Following the binding of the molecular glue VTP‐32 to the 3A dimer, Dicer is able to regain access to and cleave the viral dsRNA. This cleavage generates a substantial amount of vsiRNAs, which are subsequently loaded into RISC, ultimately guiding the degradation of the viral genome. (b) Noninfected or EV‐A71‐infected 293T or 293T cells knockout Dicer (293T‐NoDice) were treated with or without VTP‐32 at one hour post‐infection (h.p.i.), as indicated. Total RNAs were extracted from cells at 24 h.p.i., and the vsiRNAs were detected via Northern blotting by using DIG‐labeled EV‐A71‐specific (‐) vsiRNA probe. (c) Schematic diagram of the reporter plasmid that transcribes the mRNA containing the EGFP open reading frame (ORF), followed by the 600–700 nt of EV‐A71 genome (EGFP‐EV‐A71_600–700_) (upper). 293T cells were transfected with the reporter plasmid, and at 4 h.p.t., cells were infected and treated with 5 µm VTP‐32 at 1 h.p.i., as indicated. At 24 h.p.i., EGFP mRNA levels were determined via qRT‐PCR. (d) 293T or (e) 293T‐NoDice cells were infected with EV‐A71 and then treated with increasing concentrations of VTP‐32 at 1 h.p.i. EV‐A71 genomic RNA levels were determined via qRT‐PCR at 24 h.p.i. (f) 293T cells were infected with EV‐A71_D23A_ and then treated with increasing concentrations of VTP‐32 at 1 h.p.i. EV‐A71 genomic RNA levels were determined via qRT‐PCR at 24 h.p.i. (g) 293T‐IFNAR1‐KO cells were infected with EV‐A71 and then treated with increasing concentrations of VTP‐32 at 1 h.p.i. EV‐A71 genomic RNA levels were determined via qRT‐PCR at 24 h.p.i. Data are means ± SEM. All experiments were independently conducted in triplicate and repeated at least twice with reproducible results.

VTP‐32 functions as a molecular glue to induce the formation of inactive 3A dimer, which raises the question of whether this interaction modulates 3A‐VSR activity. Given that 3A‐VSR mediates immune evasion by suppressing the viral siRNA production during viral replication, we hypothesized that VTP‐32 might unlock the RNAi pathway, leading to enhanced viral siRNA production mediated by Dicer, thereby facilitating vsiRNA loading into the RISC complex for viral genome degradation (Figure 4a). To test this, we performed small RNA Northern blotting analysis, and the results revealed vsiRNA production in EV‐A71‐infected 293T cells treated with VTP‐32, whereas no vsiRNA was detected in uninfected cells, no VTP‐32‐treated cells, or VTP‐32‐treated Dicer‐knockout cell lines (Figure 4b), confirming vsiRNA generation depends on the RNAi antiviral pathway.

To further validate VTP‐32's ability to induce viral RNA degradation, we constructed an EGFP reporter system containing EV‐A71 target sequences. VTP‐32 treatment significantly reduced EGFP expression (Figure 4c), directly demonstrating viral RNA degradation. Key genetic evidence was obtained in 293T‐NoDice cells, where the antiviral activity of VTP‐32 was markedly diminished (Figure 4d,e). Further support came from experiments using an EV‐A71 VSR‐active‐site mutant (EV‐A71_D23A_). This mutant carries a D23A substitution in the 3A protein—a site essential for dimerization and VSR function—which attenuates VSR activity [21] (Figure 4f), thereby providing dual confirmation of RNAi pathway dependence. Notably, VTP‐32's antiviral efficacy remained unchanged in IFNAR1*^−/−^* 293T cells (Figure 4g), and no interferon pathway activation was observed in uninfected VTP‐32‐treated cells (Figure S5e), excluding interferon involvement. Thus, VTP‐32 forms an inactive and abnormal dimer with 3A that disrupts its VSR function, releasing dsRNA from 3A‐VSR complexes for Dicer processing into vsiRNA. Subsequent RISC loading enables targeted viral genome degradation. This study demonstrates how viral proteins can be converted into functionally defective forms through molecular glue strategies, thereby reactivating the host's innate antiviral defense system.

### 2.5 In Vivo Protective Efficacy and Safety of VTP‐32

To determine whether molecular glue VTP‐32 possesses antiviral activity in vivo, we validated its efficacy in a mouse model. Given that hand, foot, and mouth disease and its associated severe neurological complications primarily impact children under five years of age, newborn mice are widely used as a murine model for EV‐A71 infection. Since VTP‐32 and several of its optimized derivatives showed potent antiviral activity at the cellular level, we further investigated their efficacy in vivo. Based on their antiviral efficacy and chemical structural diversity, we selected VTP‐42, VTP‐46, VTP‐47, and VTP‐48, together with VTP‐32, for in vivo safety assessment. ICR suckling mice received a single intraperitoneal administration of the tested peptidomimetics at 500 mg/kg and were then observed for 16 days (Figure S6a). Survival rate and body weight measurement indicated that VTP‐32, VTP‐42, and VTP‐46 exhibited favorable safety profiles, with all mice surviving and gaining body weight normally. In contrast, VTP‐47 and VTP‐48 displayed varying toxicity: VTP‐47 treatment caused one death out of three mice, while VTP‐48 led to the death of all mice (Figure S6b,c). Therefore, VTP‐32, VTP‐42, and VTP‐46 were selected for further in vivo efficacy evaluation.

**FIGURE 5:**
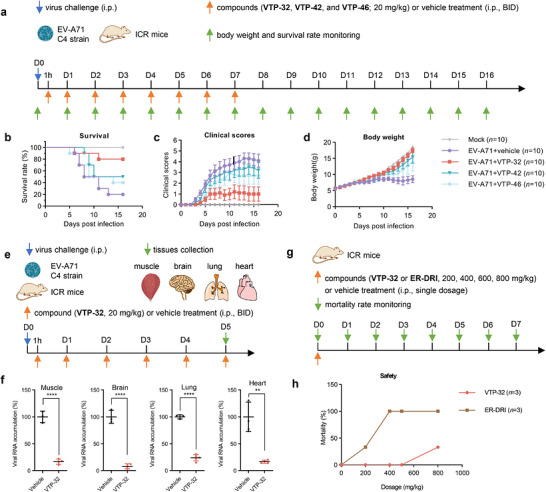
Protective activity of VTP‐32 against EV‐A71 infection in vivo. (a) Groups of ICR mice (8 days old) were challenged with 5 × 10^7^ plaque‐forming unit (PFU) EV‐A71 strain C4 via intraperitoneal injection (i.p.) and treated with VTP‐32, VTP‐42, and VTP‐46 (*n* = 10) at 20 mg/kg of body weight or vehicle (*n* = 10) as a control. Mock, noninfected, and nontreated mice (*n* = 10). (b) Mouse survival was observed and recorded daily until 16 d.p.i. (c) Clinical scores attained for the different groups of mice in (a) were tracked for 16 days. Score 5 indicates death. (d) Body weight changes of the different groups of mice in (a). (e) Groups of ICR mice (8 days old) were challenged with 5 × 10^7^ PFU EV‐A71 strain C4 via i.p. injection and treated with VTP‐32 (*n* = 4) at 20 mg/kg of body weight or vehicle (*n* = 3) as a control. Tissue samples were collected from the mice five days post‐infection for viral load detection. (f) Viral RNA loads in muscles, brains, lungs, and hearts of EV‐A71‐infected ICR mice treated with VTP‐32 or vehicle in (e). (g) Groups of ICR mice (8 days old) were administered a single intraperitoneal injection of VTP‐32 at the indicated dosage, and ER‐DRI was used as a control. (h) The mortality of mice was detected in increasing doses of VTP‐32 and ER‐DRI. *p*‐value is from a non‐parametric Mann‐Whitney test. ^**^, *p* ≤ 0.01, ^****^, *p* ≤ 0.0001.

We utilized a virulent strain of EV‐A71 C4 to intraperitoneally challenge eight‐day‐old ICR mice, which are known to be susceptible to lethal outcomes from this strain. Following the infection, tested peptidomimetics were treated one hour post‐infection, with subsequent treatment twice daily for seven consecutive days. Body weight, clinical scores, and survival rates were monitored until 16 days post‐infection (Figure 5a). The results indicated that a dosage of 20 mg/kg of VTP‐32, VTP‐42, and VTP‐46 significantly improved survival rates to 60%, 30%, and 20% among the EV‐A71‐infected mice, respectively, compared to the mock group (Figure 5b). Furthermore, peptidomimetics treatment significantly alleviated the clinical symptoms (Figure 5c) and helped maintain normal body weight (Figure 5d). Among them, VTP‐32 treatment produced the most pronounced therapeutic effect. In a dose‐response study, ICR suckling mice were treated with 10 or 50 mg/kg of VTP‐32. Increasing the dosage from 10 to 50 mg/kg raised the survival rate from 33% to 75% (Figure S6d,e), demonstrating dose‐dependent antiviral efficacy in vivo. Improvement in clinical symptoms and body weight change also followed a dosage‐dependent trend consistent with survival outcomes (Figure S6f,g). Finally, mice were treated twice daily with 20 mg/kg VTP‐32 for 4 days followed by a morning dose on day 5, and tissues were collected in the afternoon for viral load analysis (Figure 5e). VTP‐32 treatment significantly reduced viral load in multiple tissues, including muscle, brain, lung, and heart (Figure 5f), further substantiating its potent in vivo antiviral activity.

Subsequently, to evaluate the in vivo toxicity of VTP‐32, we assessed the acute toxicity of a high dose of VTP‐32 on mouse survival. In parallel, we included our previously developed 3A‐VSR‐targeting peptide ER‐DRI, which requires CPP conjugation for activity, as a control and assessed in vivo toxicity under the same conditions. Both compounds were administered via intraperitoneal injection to 8‐day‐old ICR mice at doses of 200, 400, 500, and 800 mg/kg. The results indicated that ER‐DRI caused significant toxicity, with one‐third of the mice dying at 200 mg/kg, and all succumbing at 400 mg/kg. In contrast, no deaths occurred in VTP‐32‐treated mice at 200, 400, and 500 mg/kg. At the highest dose of 800 mg/kg, only one‐third of the mice died (Figure 5g,h). As mentioned above, ER‐DRI depends on a cell‐penetrating peptide for its antiviral activity both in vitro and in vivo. This CPP dependency not only limits the drug‐likeness of ER‐DRI but also raises potential toxicity concerns. In contrast, VTP‐32 is a CPP‐free peptidomimetic with substantially improved drug‐like properties. These findings indicate that VTP‐32 has a markedly better safety profile than ER‐DRI, supporting its potential as a safer and more effective therapeutic candidate.

## 3 Discussion

Molecular glues are a class of small molecules that can induce or stabilize protein‐protein interactions (PPIs) and have emerged as a promising drug discovery strategy in recent years. However, the rational design of molecular glues remains challenging due to their complex and often unpredictable mechanisms of action, as well as the lack of generalizable design principles. In this study, we developed a general strategy for molecular glue design by redirecting pre‐existing PPIs into abnormal complexes that disrupt physiological functions. Specifically, many proteins rely on homodimerization or oligomerization for their physiological functions, providing a rationale for designing molecular glues that distort these native interactions into dysfunctional assemblies. As a proof of concept, we targeted the enterovirus nonstructural protein 3A to design broad‐spectrum anti‐enteroviral molecular glues. Enteroviral 3A forms a homodimer that binds viral dsRNA, protecting it from cleavage by host Dicer and thereby suppressing the RNAi pathway to facilitate viral immune evasion [21]. Previous 3A‐targeting peptides exhibited excellent antiviral potency in cells and mice by blocking 3A homodimerization in an RNAi‐dependent manner [27]. Based on the dimerization mechanism and the dimeric interface of the enterovirus 3A protein, we rationally designed molecular glues that simultaneously bind two 3A monomers. By linking two peptidomimetics derived from the 3A α2 helices that each interact with a separate 3A monomer, a complex structure was formed between the peptidomimetics and the two protein monomers, like a sandwich shape. This disrupts the native dimer, resulting in abnormal dimerization and loss of function. Unlike the earlier ER‐DRI, which acts as a dimerization blocker requiring a cell‐penetrating peptide (CPP) and shows obvious toxicity, VTP‐32 functions as a CPP‐free molecular glue that effectively brings two 3A monomers together into an aberrant, inactive dimer with low toxicity. This rationally designed molecular glue holds promise for application to other dimeric molecules or even interacting proteins, disrupting their native binding states to impair physiological function.

Viral suppressors of RNAi (VSRs) have emerged as promising therapeutic targets, as their pharmacological disruption has been shown to elicit a potent interferon‐independent antiviral RNAi response [27, 28]. This underscores the potential of targeting VSRs to activate RNAi‐mediated antiviral immunity as a highly attractive drug design strategy. The enterovirus 3A protein has been identified as a VSR and is highly conserved across different groups of enteroviruses, and exerts physiological function by homodimerization. These features establish 3A as an ideal target for the design of broad‐spectrum anti‐enteroviral molecular glues. In this work, the molecular glue VTP‐32 exhibited potent and broad‐spectrum antiviral efficacy against multiple enteroviruses, including EV‐A71 (group A), CV‐A16 (group A), CV‐A10 (group A), Echo 11 (group B), and EV‐D68 (group D). VTP‐32 binds two 3A monomers to form a dysfunctional dimer, thereby releasing viral dsRNA produced during viral RNA replication and restoring the host RNAi‐mediated antiviral response. This target offers considerable druggability and is expected to accelerate the development of broad‐spectrum antiviral agents.

Moreover, this antiviral strategy may be particularly suitable for immunocompromised patients with deficient interferon responses. Such individuals often have a struggle to mount an effective interferon response upon viral invasion but can still combat viruses through RNAi‐mediated antiviral immunity [19, 21]. Additionally, this strategy does not induce excessive inflammatory responses, thereby better protecting the host from collateral damage. Furthermore, EV‐A71 is not the only virus with a dimerized form of VSR; several other viruses exhibit similar VSR characteristics as EV‐A71, including NS1 of influenza A virus, B2 of nodavirus, p19 of tombusvirus, and nucleocapsid (N) protein of SARS‐CoV‐2 [21, 28, 34, 35, 36]. Focusing on the dimerization interface of various other VSRs could be a promising approach for combating medically important viruses. This study holds promise for developing first‐in‐class antiviral drugs that act via a novel mechanism (RNAi activation) against new targets (VSRs), thereby expanding the therapeutic arsenal to combat viral diseases.

## 4 Methods

### 4.1 Cells and Viruses

The RD, 293T, Vero, H1‐HeLa, Neuro‐2A cell lines were commercially obtained from ATCC and cultured at 37°C in a humidified atmosphere with 5% CO_2_ in Dulbecco's modified Eagle's medium (DMEM) (Gibco) supplemented with 10% fetal bovine serum (FBS) (Gibco), 100 U/mL penicillin, and 100 µg/mL streptomycin (Hyclone). The NoDice 293T cell line [39] was generously provided by Dr. Bryan R. Cullen (Durham, NC, USA). The IFNAR1 knockout 293T cell line used in this study was previously established in our laboratory [28].

EV‐A71 strain H (VR‐1432) was obtained commercially from ATCC, EV‐A71 C4 strain used in mice was provided by Prof. Shi Weifeng (Shanghai, China). And XY833, a clinical isolate of EV‐A71 strain, SY‐304, and a clinical isolate of CV‐A16 strain were provided by Hubei Province Center for Disease Control and Prevention (Hubei, China). The CV‐A10 was obtained from Microbial Culture and Virus Collection Center of Wuhan Institute of Virology, CAS (Hubei, China). EV‐D68 was kindly provided by Professor Wei Wei (Jilin, China). Echovirus 11 was provided by the Guangzhou Women and Children Medical Center. The VSR‐deficient EV‐A71, EV‐A71_D23A_, was described previously [21]. All viruses were amplified, and titers were determined in RD cells as previously described [40]. The viruses were concentrated by ultrafiltration using Amicon Ultra‐15 filters (Millipore) and quantified by plaque assay. Virus stock was diluted to 0.5 multiplicity of infection (MOI) for in vitro experiments.

### 4.2 Animals

Institute of Cancer Research (ICR) mice were purchased from the Beijing Vital River Laboratory Animal Technology Co., Ltd. (Beijing, China). All experiments were performed according to protocols approved by the Institutional Animal Care and Use Committee of the Wuhan Institute of Virology, CAS (WIVAF24202207).

### 4.3 RNA Extraction Quantitative Reverse Transcription‐PCR

Total RNAs were extracted using Trizol reagent (Takara) or Total RNA kit (Foregene) according to the manufacturer's instructions. For the samples required for the detection of both vsiRNAs and viral genomic RNAs, small RNA‐enriched total RNAs were isolated using the miRNeasy Mini kit (Qiagen) according to the manufacturer's instructions. Quantitative reverse transcription‐PCR (qRT‐PCR) was performed after RNA extraction. Total RNAs were extracted using a Foregene Total RNA kit according to the manufacturer's instructions. Then qRT‐PCR was performed using the specific primers for EV‐A71 genome, EGFP ORF, the indicated IFN‐I and cytokine pathway products, and the universal primers of enterovirus for CV‐A16 genome, CV‐A10 genome, Echo 11 genome, and EV‐D68 genome. The primer list is shown in Table S1.

### 4.4 Assays for Antiviral Activity

We used different methods to measure the antiviral activity of peptidomimetics, including TCID_50_ assay, plaque assay, qRT‐PCR, and CCK‐8 reagent (Yeasen). The plaque assay is the direct method for detecting a specific number of viruses. Briefly, 12‐well plates were seeded with RD cells, followed by infection with approximately 100 P.F.U. EV‐A71, and 1 h later, serially 2‐fold‐diluted peptidomimetics were added to the infected cells with an incubation time of 4 h. Then, the supernatants were replaced by fresh DMEM containing 2% low‐melting‐point agarose, 6% FBS, 200 µg/mL streptomycin, and 200 U/mL penicillin (HyClone). The plaques were incubated for 2 days postinfection (d.p.i.) until they were evident. The cells were stained and fixed with phosphate‐buffered saline (PBS) containing 1% crystal violet and 4% formaldehyde at 4°C for 2 h. After the agarose was washed away, the plaque reduction was calculated.

TCID_50_ assay and qRT‐PCR were performed after infection and treatment. Briefly, 24‐well plates were seeded with cells, followed by infection with EV‐A71 at a multiplicity of infection (MOI) of 0.1. One hour later, the diluted peptides were added to infected cells. At 24 h postinfection (h.p.i.), the supernatant was inoculated onto confluent cell monolayers in a 96‐well plate. The plate was incubated under appropriate conditions for several days. Wells were then examined for virus‐induced cytopathic effect (CPE) under a microscope. The Reed‐Muench method was subsequently used to calculate the TCID_50_/mL based on the proportion of CPE‐positive wells at each dilution. Cells were harvested, and RNAs were extracted to perform qRT‐PCR with the specific primers for EV‐A71 and universal primers of enterovirus for CVA16, CVA10, Echo 11, and EVD68. The primer list is shown in Table S1.

The colorimetric viral infection assay using the CCK‐8 reagent was performed as follows. 96‐well plates were seeded with RD cells, followed by infection with EV‐A71 at an MOI of 0.1 for 1 h. Then the infected cells were treated with the diluted peptidomimetics for about 24 h.p.i. until an evident cytopathic effect (CPE) could be observed, at which point CCK‐8 reagent was added into the well for 2 h. Finally, the optical density at 450 nm (OD450) was measured using a microplate reader, and the data were analyzed.

### 4.5 Cell Viability Test

CCK‐8 reagent was used to evaluate the cytotoxicity of peptidomimetics in RD, Vero, H1‐HeLa, and Neuro‐2A cell lines. Briefly, 96‐well plates were seeded with cells and incubated with two‐fold‐increasing concentrations of peptidomimetics. After incubation for 24 h at 37°C, CCK‐8 solution was added to the well for 2 h, followed by measurement of the value with a microplate reader.

### 4.6 Cellular Fluorescent Imaging

RD cells were seeded in 35‐mm dishes and cultured overnight. They were then treated with 2.5 mm FITC‐labeled VTP‐32 (FITC‐VTP‐32) for 24 h. After treatment, the cells were fixed with 4% paraformaldehyde for 15 min at room temperature, followed by blocking and permeabilization steps performed as previously described [41]. Images were acquired using a confocal microscope (Olympus FV1000).

### 4.7 Northern Blotting

For Northern blotting analysis of small RNAs, 20 µg of small RNA‐enriched total RNAs were resolved on 8 M urea‐18% PAGE, transferred to Hybond‐A nylon membrane (GE Healthcare), and chemically cross‐linked using 1‐ethyl‐3‐(3‐dimethylaminopropyl) carbodiimide (EDC) (Sigma) as previously described [42]. The small RNA markers and digoxin (DIG)‐labeled oligo RNA probes targeting EV‐A71 vsiRNAs and U6 were synthesized by Takara as previously described [21].

### 4.8 Plasmids, Protein Expression, and Purification

The shRNA‐induced RNAi and the EGFP‐EV‐A71_600–700_ reporter silencing assay were performed as previously described [21] in the presence or absence of 10 mm VTP‐32. EV‐A71 3A protein was cloned into the mammalian expression vector pRK‐HA. Plasmids for the purification of MBP fusion protein 3A were constructed by inserting the 3A ORF into the pMAL‐c2X vector.

For the expression and purification of recombinant MBP‐3A, the constructs were expressed in *E. coli* strain TB1 at 27 °C with 0.2 mm IPTG induction. Cells were harvested and resuspended in binding buffer [20 mm Tris‐HCl (pH 7.4), 200 mm NaCl, 1 mm EDTA, 10 mm 2‐mercaptoethanol] containing 1.5% Triton X‐100 and a protease inhibitor cocktail (Roche). After sonication, the lysate was clarified by centrifugation at 11,000  ×  g for 30 min. The supernatant was subjected to affinity purification using amylose resin (New England BioLabs) according to the manufacturer's instructions, followed by concentration with Amicon Ultra‐15 filters (Millipore). Protein concentrations were determined using a UV–vis spectrophotometer (Shimadzu).

### 4.9 Western Blotting

Cells were harvested in lysis buffer containing 50 mm Tris‐HCl (pH 7.4), 150 mm NaCl, 1% NP‐40, 0.25% sodium deoxycholate, and a protease inhibitor cocktail. For native analyses (Figure 3d–g), lysates were mixed with native protein loading buffer (typically containing glycerol, bromophenol blue, and optionally a non‐ionic tracking dye, but without SDS or reducing agents) and resolved on 15% Native‐PAGE gels. For denaturing analysis (Figure 3h), samples were treated with denaturing protein loading buffer (containing SDS, *β*‐mercaptoethanol or dithiothreitol (DTT), glycerol, bromophenol blue, and Tris‐HCl buffer), boiled at 95 °C for 10 min, and then separated on 15% SDS‐PAGE gels for Western blotting according to our standard procedures [43]. The anti‐3A polyclonal antibody was raised in rabbits against peptide GPPKFKPIKISLEEC (GenScript antibody service, Nanjing, China) as previously described [21]. The FITC‐antibody was purchased from Proteintech (68132‐1‐lg).

### 4.10 Surface Plasmon Resonance

The affinity measurements were performed using a Biacore 1K instrument. Additionally, 3A was covalently coupled to the CM5 sensory chips via amine coupling to a response unit (RU) of 10,000 RU. For the Biacore studies, Binding measurements were performed at 25°C and a flow rate of 30 µL/min. VTP‐32 was diluted in PBS containing 0.05% Surfactant P20 (Cytiva) and injected over the chip for 120 s, followed by a 300‐second dissociation phase. After each injection, the chip was regenerated using 0.5% SDS. Binding curves were exported for analysis using GraphPad Prism, and steady‐state affinity was calculated using Biacore Insight Evaluation Software.

### 4.11 Isothermal Titration Calorimetry

Isothermal titration calorimetry (ITC) experiments were conducted using a Nano‐ITC calorimeter at 25°C. The sample cell was loaded with a 100 µm 3A protein solution, while the syringe contained a 1.0 mm VTP‐32, maintaining a 10:1 compound‐to‐protein ratio. The experimental protocol included an initial 0.2 µL injection, followed by 26 subsequent 1.5 µL injections. The stirring speed was set to 750 rpm. The titration data were analyzed using Origin 7.0 software by fitting the heats of binding to a one‐site binding model.

### 4.12 3A Protein and FITC‐VTP‐32 Binding Experiment

Incubate MBP‐3A (2 mg/mL) protein with FITC‐VTP‐32 (200 µm) at 4°C overnight, and run a Native‐PAGE gel the next day, followed by Western blotting analysis using FITC‐specific antibody and 3A‐specific antibody, respectively. The tag‐free 3A protein is obtained by cleaving the site between MBP and 3A using Factor‐Xa (NEB) to remove the MBP tag. MBP‐3A (2 mg/mL) is incubated with Factor‐Xa (NEB) at 4°C overnight before proceeding with the above steps.

### 4.13 In‐gel Based ABPP

We first synthesized PAL‐VTP‐32, then incubated 2 mg/mL MBP‐3A protein with 200 µm PAL‐VTP‐32 at 4°C overnight. The incubation mixture was cross‐linked under 254 nm ultraviolet light for 1, 3, and 5 min, followed by the addition of 100 µm N_3_‐Rhodamine (MCE), 1 mm copper sulfate, 5 mm ascorbic acid, and 0.1 mm TBTA. Finally, 10 mm EDTA was added and incubated at room temperature for 5–10 min to terminate the reaction. The samples were analyzed by Native‐PAGE, and Rhodamine signals were detected using Typhoon, while 3A protein levels were assessed by Western blotting.

### 4.14 Antiviral Efficacy of Peptidomimetics in Newborn ICR Mice

The progenies of the pregnant ICR mice were assigned randomly to three groups, and each group had eight newborn mice. After 8 days, the 8‐day‐old newborn mice in the vehicle and therapeutic groups were challenged with 5 × 10^7^ P.F.U. EV‐A71 C4 strain intraperitoneally, while the mice in the mock group were i.p. injected with the same volume of PBS. At 1 h post‐challenge, the mice were i.p. injected with 20 mg/kg VTP‐32, VTP‐42, VTP‐46, or vehicle for the first time. VTP‐32 was i.p. injected twice a day for 7 consecutive days. The suckling mice were monitored daily for body weight, clinical symptoms, and mortality for 16 days. A score was used to evaluate the clinical symptoms as previously described: 0, healthy; 1, hunchbacked and slow movement; 2, weakness in one limb; 3, paralysis in one limb; 4, paralysis in both limbs; and 5, death [44, 45]. In addition, another two groups (viral control and treatment) of mice were administered with the same methods except that these mice were sacrificed at 5 days post‐infection (d.p.i.) to determine the virus load in their organs via qRT‐PCR. The peptidomimetics were dissolved in ultrapure water with a concentration of 10 mg/mL for the experiment. Isoflurane was administered as an anesthetic via inhalation at a concentration of 3–4% prior to infection or drug administration. Following the procedure, euthanasia was performed by cervical dislocation while the animal was under anesthesia.

### 4.15 In Vivo Safety

The 8‐day‐old ICR mice were assigned randomly to three groups, and each group had 3 mice. The mice received a single injection of VTP‐32 (200, 400, 500, or 800 mg/kg; *n* = 3 per group), ER‐DRI (200, 400, 500, or 800 mg/kg; *n* = 3 per group), or an equivalent volume of vehicle (*n* = 3). Body weights and survival rates were monitored for 16 days. The peptidomimetics were dissolved in ultrapure water at the indicated concentration.

### 4.16 Synthesis of Peptides

These peptides, summarized in Table S2, were synthesized by KareBay Biochem with more than 95% purity, as analyzed by HPLC.

### 4.17 Synthesis of Designed Peptidomimetics

#### 4.17.1 General Procedure

A combined method of solution‐ and solid‐phase peptide synthesis was applied to prepare these designed peptidomimetics, and the detailed information was described in the Supplementary Information. All reagents and solvents were purchased from commercial sources and used without further purification. The reaction monitoring was performed by ninhydrin reagent staining or by liquid chromatography‐mass spectrometry (LC‐MS). All targeted peptidomimetics were purified by PHPLC and analyzed by HPLC (Agilent 1290), and the purities are more than 95%. Low‐resolution mass spectrometry (LRMS) data were recorded on Agilent 6125B, SHIMADZU LCMS‐2020, or Waters 2695 with electrospray ionization (ESI).

#### 4.17.2 Abbreviations of Reagents Involved in the Synthesis of Peptidomimetics

HATU: 2‐(7‐Azabenzotriazol‐1‐yl)‐*N*,*N*,*N*’,*N*’‐tetramethyluronium hexafluorophosphate; DMF: *N*,*N*‐dimethylformamide; DIPEA: *N*,*N*‐diisopropylethylamine; DCM: dichloromethane; MeOH: methanol; MTBE: *tert*‐butyl methyl ether; TFA: trifluoroacetic acid; EDT: 1,2‐ethanedithiol; TIS: triisopropylsilane; Cbz‐OSu: *N*‐(Benzyloxycarbonyloxy)succinimide; HFIP: 1,1,1,3,3,3‐hexafluoro‐2‐propanol;

#### 4.17.3 Rink Resin Activating and First Amino Acid Loading

##### 4.17.3.1 Rink Resin Swelling

To the Rink resin with a loading of 0.405 mmol/g was added an appropriate amount of DMF, and the mixture was kept for 2 h at room temperature to swell and activate. The resin was filtered and directly used in the next step (Scheme S1).

##### 4.17.3.2 The Fmoc Group Removing

To the swollen and activated Rink resin was added an appropriate amount of a mixed solvent (piperidine/DMF = 1/4, v/v), and the resulting mixture was shaken for 30 min at room temperature. The resin was filtered and washed five times with DMF, and the ninhydrin staining showed blue.

##### 4.17.3.3 The First Amino Acid Loading

A solution of indicated amino acid (Fmoc‐AA‐OH, 3.0 eq.), HATU (3.0 eq.), and DIPEA (3.0 eq.) in an appropriate DMF was added to the resin. The mixture was shaken until the resin was transparent when stained by ninhydrin. Then the resin was filtered, washed five times with DMF, and used in the next step.

#### 4.17.4 2‐CTC Resin Activating and First Amino Acid Loading

To the 2‐CTC resin with a loading of 1.2 mmol/g was added an appropriate amount of DCM, and the mixture stayed for 30 min at room temperature to swell and activate (Scheme S2). Then Fmoc‐AA‐OH (2.0 eq.) and DIPEA (4.0 eq.) were added, and the resulting mixture was shaken for 1 h. The resin was filtered and washed five times with DCM. A mixed solvent of DCM/MeOH/DIPEA = 17/2/1 (v/v/v) was added to the resin and reacted for 15 min to endcap any remaining reactive trityl chloride group. The resin was filtered and washed five times with DCM and DMF, respectively, and directly used.

#### 4.17.5 Peptide Elongation

According to the above‐mentioned general procedure, remove the Fmoc group of the first amino acid loaded on the resin. Then, the amide condensation was performed by using the HATU/DIPEA system as aforementioned. These two steps were repeated until the targeted peptide sequence was obtained.

#### 4.17.6 Introduction of N‐Terminus Caps

To introduce cap groups into the *N*‐terminus of amino acid loaded on the resin, the corresponding N‐cap reagent (2.0 eq., the chemical structure was shown in Scheme S3) and DIPEA (2.0 eq.) were added sequentially to the suspension of peptide‐coupled resin with *N*‐terminal free amine groups in DMF. The resulting mixture was gently shaken until the resin was transparent when stained by ninhydrin. The resin was filtered and washed five times with DCM, DMF, and MeOH, respectively. The dried resin was directly used.

#### 4.17.7 Selective Removal of the 2‐CTC Resin

To a peptide conjugated resin was added an appreciable amount of a mixed solution of HFIP/DCM (1/4, v/v), and the mixture was shaken for 1 h. The mixture was filtered, and the resin was washed twice with the mixed solution of HFIP/DCM (1/4, v/v). The combined filtrate was continuously blown by a stream of nitrogen to remove most of the solvent, and the residual was added 6 volumes of MTBE to precipitate the targeted peptides. The suspension was filtered, and the cake was washed eight times with MTBE and dried in vacuo to obtain the crude peptide as a white solid. The crude compound was used in the next step without additional purification.

#### 4.17.8 Introduction of C‐Terminus Caps

##### 4.17.8.1 General Procedure 1

The crude peptide was dissolved in an appropriate amount of DMF, and the mixture was cooled under an ice‐water bath. The corresponding amine (1.05 eq., Scheme S4), HATU (1.5 eq.), and DIPEA (3.0 eq.) were added sequentially, and the resulting mixture was warmed to room temperature and stirred for 3 h. The mixture was continuously blown by a stream of nitrogen to remove most of the solvent, and 6 volumes of MTBE were added. The precipitated white solid was filtered, washed eight times with DMF, dried *in vacuo*, and directly used in the next step.

##### 4.17.8.2 General Procedure 2

To a solution of the crude peptide in an appropriate amount of DMF was added benzyl bromide (2.0 eq.) and DIPEA (2.0 eq.) at room temperature, and the resulting mixture was stirred for 2 h. Then, most of the solvent was evaporated by a continuous stream of nitrogen gas. The residual was added 6 volumes of MTBE and the precipitated white solid was filtered, washed eight times with DMF, dried in vacuo, and directly used in the next step without any purification.

#### 4.17.9 Cleavage of Protected Groups of Side Chains of Amino Acids and/or Resin

The crude peptide or peptide‐loaded resin was charged with an appropriate amount of DMF, and then an appropriate amount of cleavage solution (TFA/EDT/H_2_O/Phenol/TIS = 90/2.5/2.5/2.5/2.5, v/v/v/v/v) was added. The resulting mixture was stirred (peptide) or shaken (resin) for 3 h at room temperature. The resin was filtered and washed with an appropriate amount of cleavage solution. The collected filtrate (peptide‐loaded resin) or the reaction solution (peptide) was continuously blown by a stream of nitrogen gas to remove most of the solvent. To the residual was added 6 volumes of MTBE, and the precipitated white solid was filtered and washed eight times with DMF. The solid was dried *in vacuo* and purified by preparative HPLC. Notation: If the peptide was not the targeted peptide for antiviral activity evaluation, it was used in the dimerization step without purification.

#### 4.17.10 Dimerization of Monomers by Disulfide Bond

To a solution of monomer peptidomimetic in a mixed solvent of water and acetonitrile (3/7, v/v) was added a solution of iodine in MeOH (2 g in 100 mL), and the resulting mixture was stirred for 30 min at room temperature. The mixture was continuously blown with nitrogen gas, and the residual was added 6 volumes of MTBE. The precipitated white solid was filtered, washed eight times with MTBE, dried in vacuo, and purified by preparative HPLC.

#### 4.17.11 Synthesis of VTP‐01 to VTP‐11 and VTP‐22 to VTP‐31

VTP‐01 to VTP‐11 and VTP‐22 to VTP‐31 were synthesized by the general routine of Rink Amide resin‐based solid‐phase peptide synthesis (Scheme S1). The involved amino acids and reagents for the introduction of *N*‐terminus caps were summarized in Scheme S5 and Scheme S3, respectively.

#### 4.17.12 Synthesis of VTP‐12 to VTP‐21

VTP‐12 to VTP‐21 were synthesized by the general routine of 2‐CTC resin‐based solid‐phase peptide synthesis (Scheme S2). The involved amino acids and reagents for the introduction of *N*‐terminus caps were summarized in Scheme S5 and Scheme S4, respectively.

#### 4.17.13 Synthesis of VTP‐32, VTP‐36 to VTP‐48

The corresponding monomers of dimer peptidomimetics VTP‐32, VTP‐36 to VTP‐48 were synthesized by the general routine of Rink Amide resin‐based solid‐phase peptide synthesis (Scheme S1). The involved amino acids and reagents for the introduction of *N*‐terminus caps were summarized in Scheme S5 and Scheme S3, respectively. Then the monomers were dimerized by the disulfide bond under the oxidation of iodine.

#### 4.17.14 Synthesis of VTP‐33

The synthesis of VTP‐33 is similar to that of VTP‐32, except that the *L*‐amino acids were replaced with corresponding *D*‐configuration ones.

#### 4.17.15 Synthesis of VTP‐34 and VTP‐35

The corresponding monomer of dimer peptidomimetic VTP‐34 was synthesized by the general routine of Rink Amide resin‐based solid‐phase peptide synthesis (Scheme S1). The corresponding monomer of dimer peptidomimetic VTP‐35 was synthesized by the general routine of 2‐CTC resin‐based solid‐phase peptide synthesis (Scheme S2). The involved amino acids are summarized in Scheme S6. The reagents for the introduction of the N‐terminus cap are Cbz‐OSu and acetic anhydride for VTP‐34 and VTP‐35, respectively. The reagent for the introduction of the C‐terminus cap of VTP‐35 is benzyl bromide. Then the monomers were dimerized by the disulfide bond under the oxidation of iodine.

#### 4.17.16 Synthesis of Probe FITC‐VTP‐32

FITC‐VTP‐32 was synthesized by the general routine of Rink Amide resin‐based solid‐phase peptide synthesis (Scheme S7). The linker loading protocol is similar to the amino acid loading. And the FITC loading procedure is similar to the introduction of *N*‐terminus caps.

#### 4.17.17 Synthesis of Probe PAL‐VTP‐32

PAL‐VTP‐32 was synthesized by the general routine of Rink amide resin‐based solid‐phase peptide synthesis (Scheme S8).

### 4.18 Purification and Characterization of Targeted Peptidomimetics

The crude peptidomimetics were purified by preparative HPLC, and the purities of the targeted peptidomimetics were tested on the same instrument. The targeted peptidomimetics were characterized by LRMS. The HPLC purity and LRMS data are summarized in Table S3.

### 4.19 Statistical Analysis

The detection of antiviral activity, including TCID_50_ assay, plaque assay, qRT‐PCR, and CCK‐8 reagent, was conducted in two independent biological experiments (*n* = 2), each consisting of three technical replicates. SPR and ITC measurements were carried out as three independent experiments for each compound‐protein pair (*n* = 3). The Protein and FITC‐VTP‐32 Binding Experiment and In‐gel Based ABPP were performed as three independent experiments (*n* = 3). Cellular Fluorescent Imaging and Northern Blotting were also carried out as three independent replicates. The in vivo antiviral study initially included ten mice per group (*n* = 10), with final group sizes reduced due to filial cannibalism, as indicated in the corresponding figure legends. For the acute toxicity test in mice, three mice per group were included (*n* = 3). For viral load detection in mouse tissues, four mice per group were initially included (*n* = 4), with final group sizes reduced due to filial cannibalism, as indicated in the corresponding figure legends. Statistical methods were performed as follows: viral RNA loads in cells were analyzed using a two‐sided unpaired *t*‐test. Differences in viral RNA loads in the sera of mice treated with VTP‐32 or vehicle were determined using the non‐parametric Mann‐Whitney test. Statistical significance was defined as ^*^ *p* < 0.05, ^**^ *p* < 0.01, and ^***^ *p* < 0.001. All statistical analyses and curve fitting were performed using GraphPad Prism.

## Author Contributions

H.L. and X.Z. conceptualized the study. H.L., X.Z., Y.F., and X.X. developed the methodology. H.L., X.Z., Y.F., X.X., H.F., B.W., A.W., W.D., Z.L., J.L., H.T., J.L., Y.R., and J.W. conducted the investigation. H.L., X.Z., Y.F., and X.X. performed the visualization. H.L., X.Z., Y.F., X.X., W.D., and J.L. acquired the funding. H.L., and X.Z. administered the project. H.L., X.Z. supervised the work. H.L., X.Z., Y.F., and X.X. wrote the original draft. H.L., X.Z., Y.F., and X.X. reviewed and edited the manuscript.

## Funding

This work was supported by the National Natural Science Foundation of China (U21A20423, 82130105, 32225004, 82404403, 82522054, 82173654, 82341091, U22A20379), and the Strategic Priority Research Program of the Chinese Academy of Sciences (XDC0200200).

## Conflicts of Interest

The authors declare no competing interests.

## Supporting information




**Supporting File**: advs75317‐sup‐0001‐SuppMat.pdf.

## Data Availability

All data generated or analyzed during this study are included in the manuscript and supporting files. Detailed information for peptidomimetics, including LRMS, and HPLC spectra results are shown in supplemental materials. All data that support the findings of the study are available from the corresponding authors upon reasonable request.
