# Supplementary material for: Rational Design of Broad‐Spectrum Anti‐Enteroviral Molecular Glues Targeting Enteroviral RNAi Suppressors
Source: Adv Sci (Weinh). 2026 Apr 16;13(38):e75317. doi: 10.1002/advs.75317 (PMC13335669; doi:10.1002/advs.75317)
Supplement: Supplementary file 1 — Supporting File: advs75317‐sup‐0001‐SuppMat.pdf. [file ADVS-13-e75317-s001.pdf]

Supporting Information

Rational Design of Broad-spectrum Anti-enteroviral Molecular Glues Targeting Enteroviral RNAi Suppressors

Yuan Fang, Xiong Xie, Huidi Fan, Botao Wu, An Wang, Wenhao Dai, Zezhong Liu, Jian Li, Huoyan Tong, Jianan Li, Yujie Ren, Jinlin Wang, Xi Zhou, Hong Liu**

Corresponding author: hliu@simm.ac.cn and zhouxi@wh.iov.cn

The file includes:

Supplementary Figures 1-6, Tables 1-3, Schemes 1-8, and the LRMS and HPLC purity data of designed and synthesized peptidomimetics.

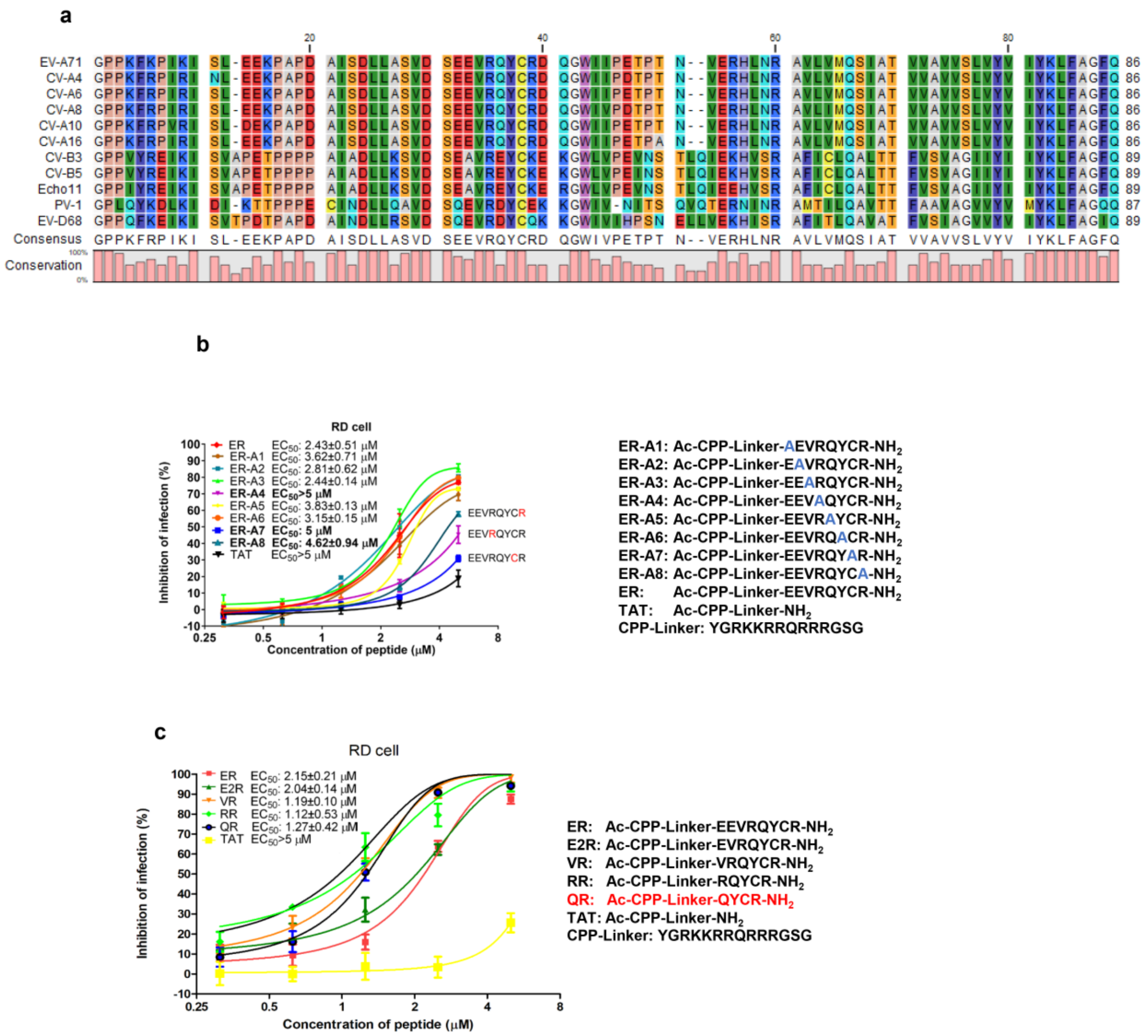

Figure S1. ***Identification of functionally critical amino acid residues in the antiviral peptide by alanine scanning and peptide truncation.*** **(a)** Comparative analysis of the amino acid sequences of the 3A proteins across enterovirus groups A, B, C, and D. **(b)** The alanine scanning of octapeptide EEVRQYCR. **(c)** The sequence truncation of the octapeptide EEVRQYCR from the *N*-terminus to the *C*-terminus. These peptides were attached with a cell-penetrating peptide ($TAT_{47\text{-}57}$) for cellular uptake. The antiviral activities were measured in EV-A71-infected RD cells.

a

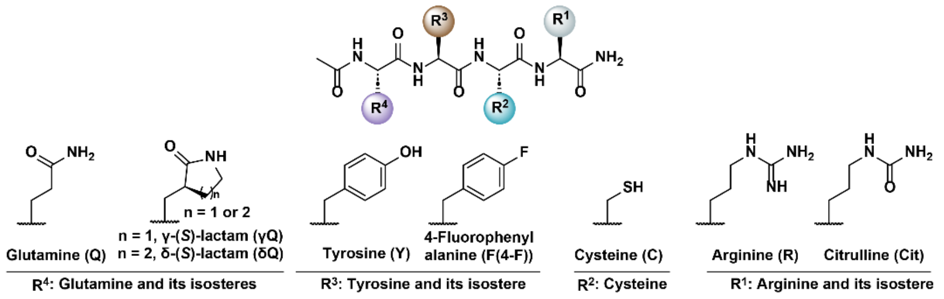

Antiviral activity against EV-A71 in RD cells

Compounds	Structures	Inhibition rate @5 μM[a]	Compounds	Structures	Inhibition rate @5 μM[a]
VTP-01	Ac-QYC-Cit-NH$_2$	NI	VTP-02	Ac-Q-F(4-F)-CR-NH$_2$	NI
VTP-03	Ac-γQ-YCR-NH$_2$	NI	VTP-04	Ac-δQ-YCR-NH$_2$	NI
VTP-05	Ac-Q-F(4-F)-C-Cit-NH$_2$	NI	VTP-06	Ac-γQ-YC-Cit-NH$_2$	NI
VTP-07	Ac-δQ-YC-Cit-NH$_2$	NI	VTP-08	Ac-γQ-F(4-F)-CR-NH$_2$	NI
VTP-09	Ac-δQ-F(4-F)-CR-NH$_2$	NI	VTP-10	Ac-δQ-F(4-F)-C-Cit-NH$_2$	8.3%

b

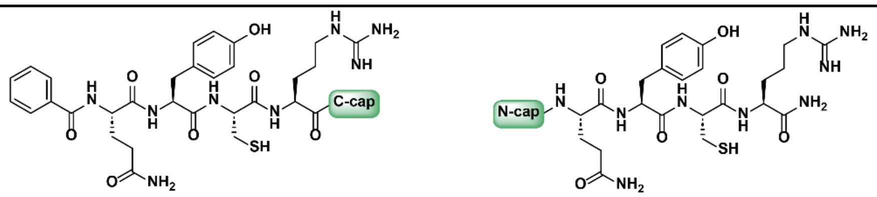

Compounds	C-cap	Inhibition rate @5 μM[a]	Compounds	N-cap	Inhibition rate @5 μM[a]
VTP-11	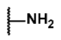	74.3%	VTP-22	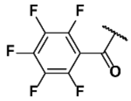	54.7%
VTP-12	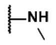	28.8%	VTP-23	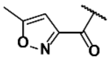	47.9%
VTP-13	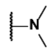	NI	VTP-24	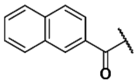	NI
VTP-14	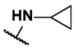	9.80%	VTP-25	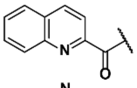	20.9%
VTP-15	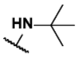	NI	VTP-26	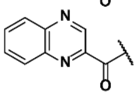	30.8%
VTP-16	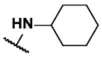	6.80%	VTP-27	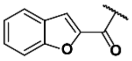	7.10%
VTP-17	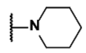	6.30%	VTP-28	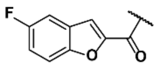	25.7%
VTP-18	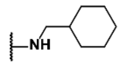	10.7%	VTP-29	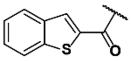	27.6%
VTP-19	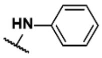	11.5%	VTP-30	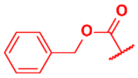	102.7%
VTP-20	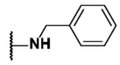	10.3%	VTP-31	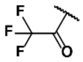	NI
VTP-21	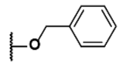	9.80%	ER-ERI		95.2%

Figure S2. Peptidomimetic modification of tetrapeptide QYCR. (a) Bioisosteric replacement of the glutamine (Q), tyrosine (Y), and arginine (R) with hydrophobic residues. **(b)** The exploration of *C*- and *N*-terminal caps. The antiviral activities were tested in EV-A71-

infected RD cells. [a] NI indicates that these peptidomimetics showed no inhibition against EV-A71 with an inhibition rate of less than 5% at a concentration of 5 μM.

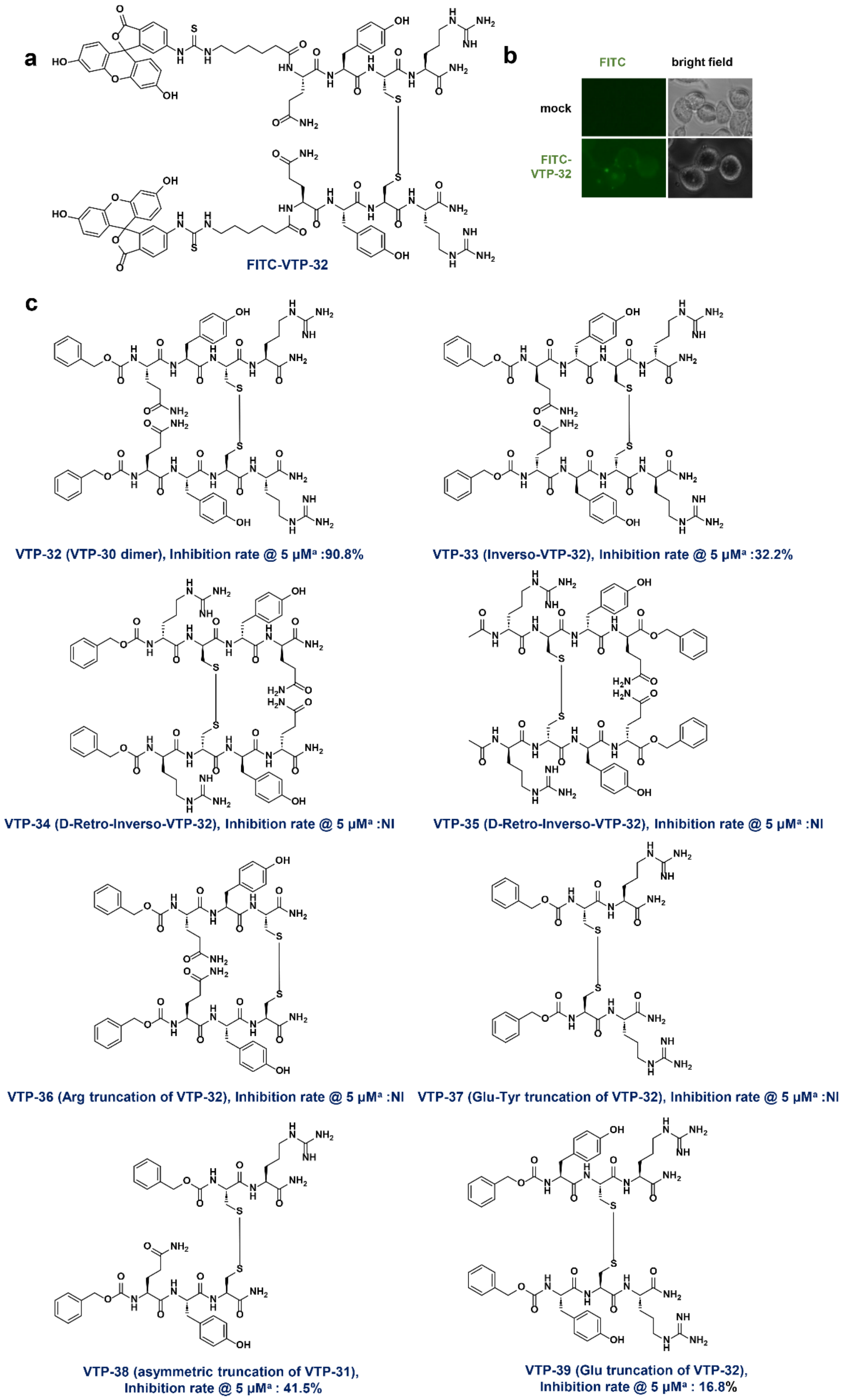

Figure S3. The modification of dimer peptidomimetic residue configuration, sequence, and size. **(a)** The chemical structure of probe FITC-VTP-32. **(b)** RD cells were incubated with 5 μM indicated FITC-labeled VTP-32 (FITC-VTP-32), followed by fluorescence microscopy

at 24 h post-treatment. Scale bar, 100 μm. **(c)** The structural modification of dimer peptidomimetics. The antiviral activities were tested in EV-A71-infected RD cells. [a] NI indicates that these peptidomimetics showed no inhibition against EV-A71 with an inhibition rate of less than 5% at a concentration of 5 μM.

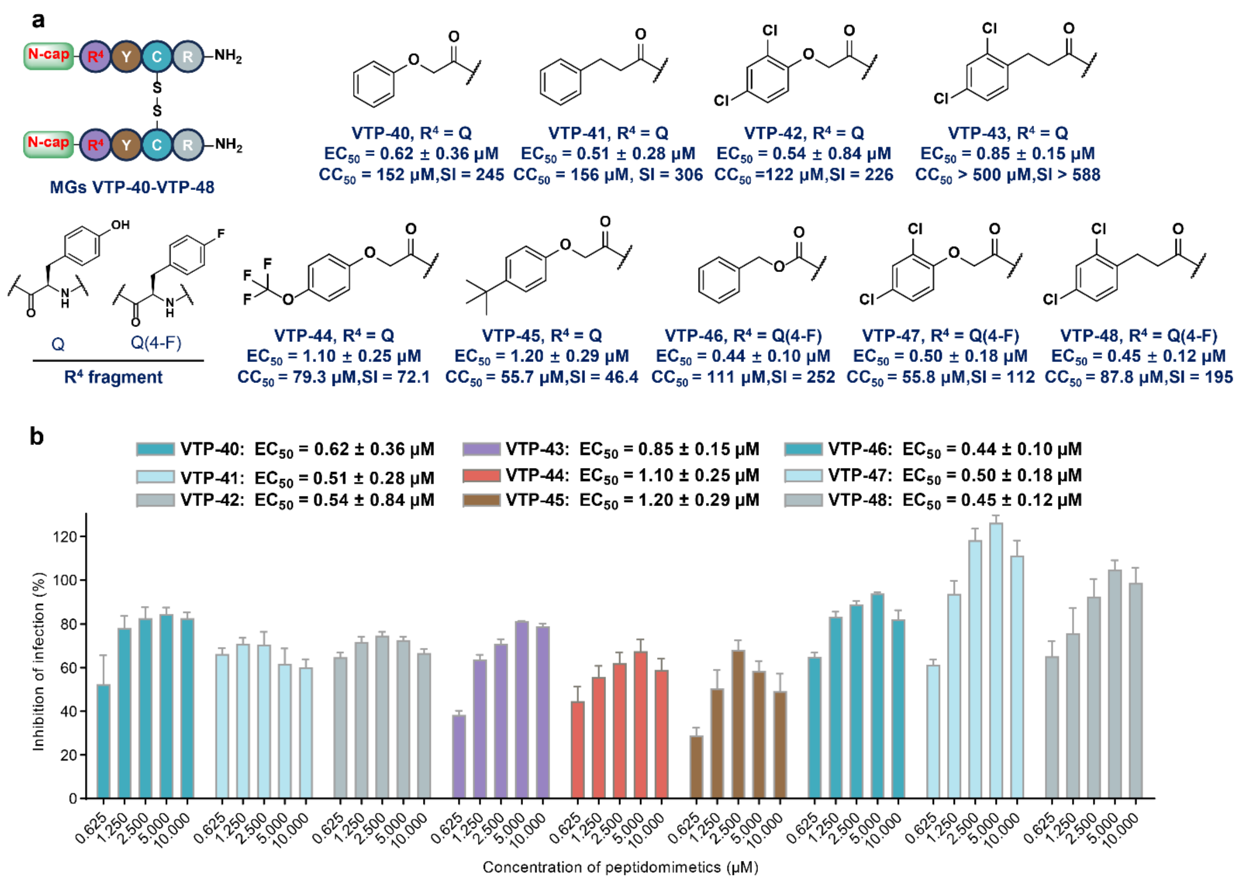

Figure S4. The peptidomimetic modification of VTP-32. The chemical structures **(a)** and the anti-EV-A71 activity **(b)** of modified peptidomimetics.

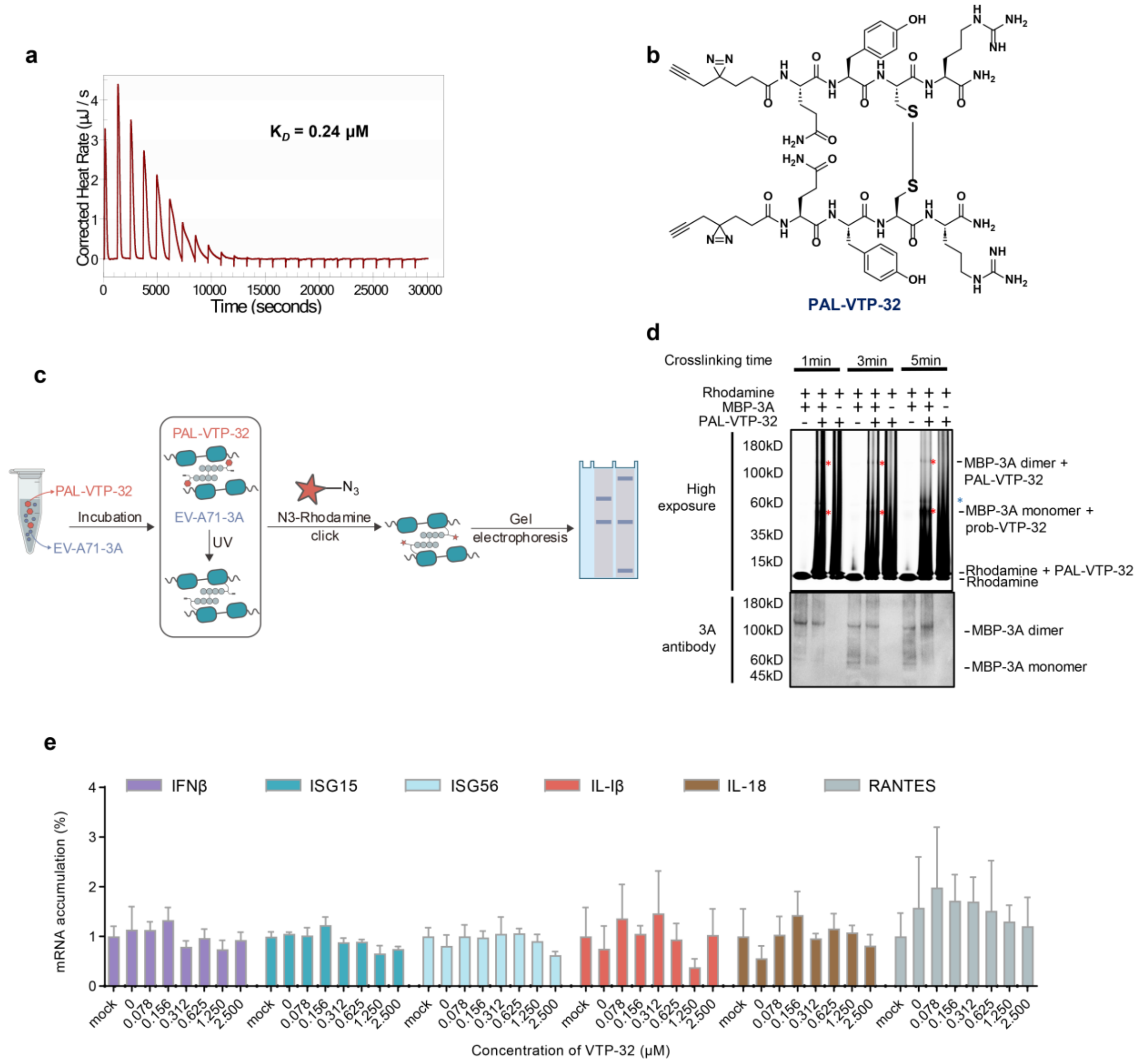

Figure S5. VTP-32 can bind to 3A protein to form a homodimer, and the antiviral activity of VTP-32 is independent on the IFN pathway. (a) ITC measurement confirms the direct binding of VTP-32 to the enteroviral 3A protein, yielding a dissociation constant (K_D) of 0.24 μM. **(b)** The chemical structure of the photoaffinity probe PAL-VTP-32. **(c)** Schematic diagram of the assay in-gel based ABPP to detect binding between PAL-VTP-32 and 3A. **(d)** The results of in-gel based ABPP to detect binding between PAL-VTP-32 and 3A. The red asterisks indicate the binding of different oligomers of the 3A protein to PAL-VTP-32. **(e)** To assess the effects of VTP-32 on interferon pathway and inflammatory cytokine pathway-related factors in RD cells, we treated RD cells with 10 μM VTP-32 for 24 hours, followed by RNA extraction and quantitative real-time PCR analysis to detect changes in the expression of relevant factors.

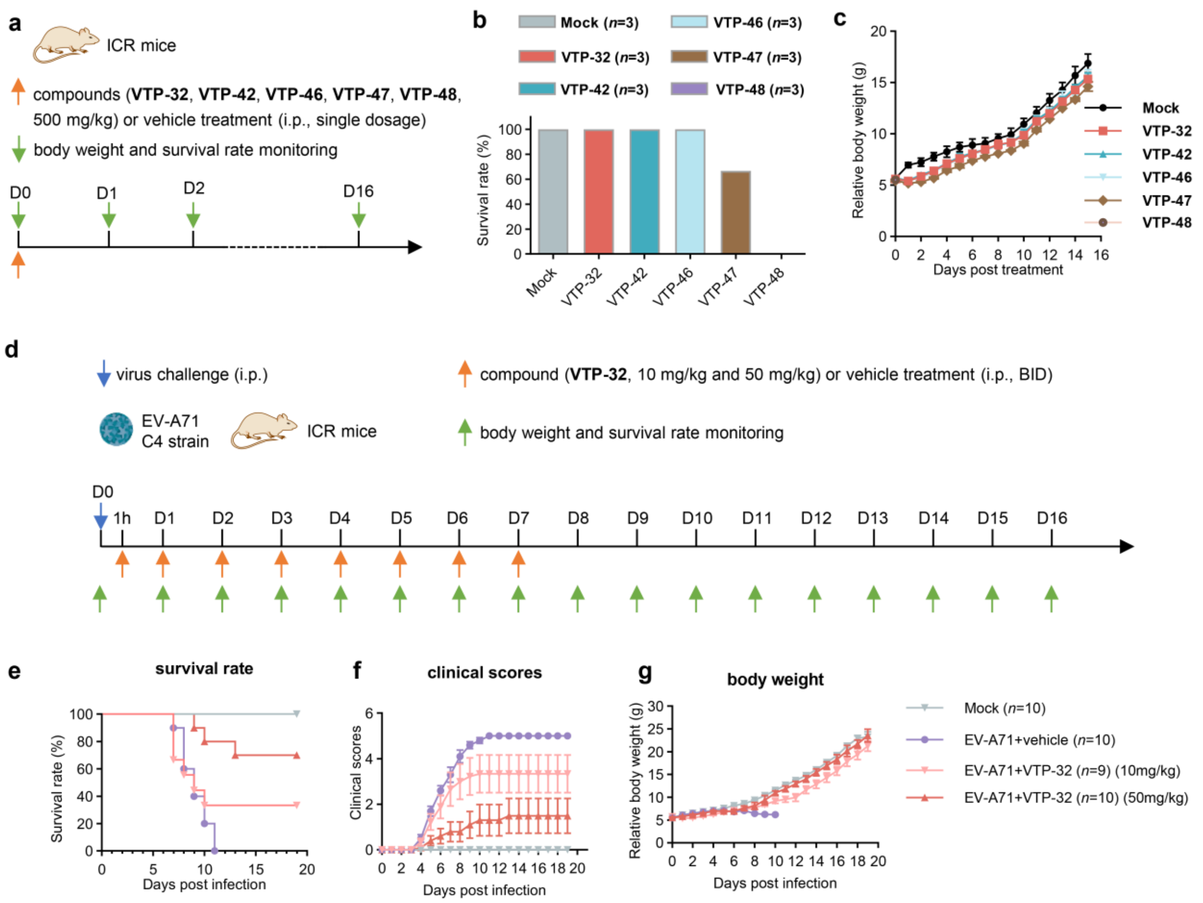

Figure S6. *In vivo* antiviral efficacy and toxicity of the representative dimers to support the selection of VTP-32 for further study. (a) The schematic procedure of *in vivo* safety evaluation of selected peptidomimetics. The survival rate **(b)** and relative body weight change **(c)** of mice treated with peptidomimetics ($n = 3$) or vehicle ($n = 3$). **(d)** The schematic procedure of *in vivo* antiviral efficacy evaluation of VTP-32 at different dosages. The survival rate **(e)**, clinical scores **(f)**, and relative body weight change **(g)** after the treatment with 10 mg/kg VTP-32 ($n = 9$), 50 mg/kg VTP-32 ($n = 10$), or vehicle ($n = 10$). Mock, noninfected, and nontreated mice ($n = 10$).

Table S1. The primers and oligonucleotides used in this study. Related to STAR Methods

Primers	Sequence (5' to 3')	Purpose
EV-A71-F	GGCCATTTATGTGGGTAACTTTAGA	Primers used for qRT-PCR
EV-A71-R	CGGGCAATCGTGTCACAAC	
Human β-actin-F	AGAGCTACGAGCTGCCTGAC	
Human β-actin-R	AGCACTGTGTTGGCGTACAG	
Mu-GAPDH-F	CATCACTGCCACCCAGAAGACTG	
Mu-GAPDH-R	ATGCCAGTGAGCTTCCCGTTCAG	
CV-A16-F	ATCCAGTAAGGATCCCAGACT	
CV-A16-R	GATTTGCATAGTGGAGAGCAG	
EV-KaresF	CGGCCCCTGAATGCGGCTAA	
EV-KaresR	GAAACACGGACACCCAAAGTA	
Monkey β-actin-F	CACACAGGGGAGGTGATAGC	
Monkey β-actin-R	GCACTTTTATTCAACTGGTCTCA	
EGFP-F	AAGGGCATCGACTTCAAGG	
EGFP-R	TGCTTGTCGGCCATGATATAG	
Human-IFNβ-F	TTGTTGAGAACCTCCTGGCT	
Human-IFNβ-R	TGACTATGGTCCAGGCACAG	
Human-ISG15-F	GAGAGGCAGCGAACTCATCTT	
Human-ISG15-R	CCAGCATCTTCACCGTCAGG	
Human-ISG56-F	TTGATGACGATGAAATGCCTGA	
Human-ISG56-R	CAGGTCACCAGACTCCTCAC	
Human-IL-1β-F	CTTCCTTCCTTCCTTCCT	
Human-IL-1β-R	CAGAGCCTCATAGCAGTA	
Human-IL-18-F	AAGGAAATGAATCCTCCTGATAACA	
Human-IL-18-R	CCTGGGACACTTCTCTGAAAGAA	
Human-RANTES-F	TACACCAGTGGCAAGTGCTC	
Human-RANTES-R	ACACACTTGGCGGTTCTTTC	

Table S2. Peptides and sequences.

Peptides	Sequences
TAT_{47-57}	Ac-YGRKKRRQRRRGSG-NH_2
ER-DRI	Ac-rcyqrveepprrrqrrkkrgy-NH_2
ER-A1	Ac-YGRKKRRQRRRGSGAEVRQYCR-NH_2
ER-A2	Ac-YGRKKRRQRRRGSGEAVRQYCR-NH_2
ER-A3	Ac-YGRKKRRQRRRGSGEEARQYCR-NH_2
ER-A4	Ac-YGRKKRRQRRRGSGEEVAQYCR-NH_2
ER-A5	Ac-YGRKKRRQRRRGSGEEVRAYCR-NH_2
ER-A6	Ac-YGRKKRRQRRRGSGEEVRQACR-NH_2
ER-A7	Ac-YGRKKRRQRRRGSGEEVRQYAR-NH_2
ER-A8	Ac-YGRKKRRQRRRGSGEEVRQYCA-NH_2
ER	Ac-YGRKKRRQRRRGSGEEVRQYCR-NH_2
E2R	Ac-YGRKKRRQRRRGSGEVRQYCR-NH_2
V2R	Ac-YGRKKRRQRRRGSGVRQYCR-NH_2
RR	Ac-YGRKKRRQRRRGSGRQYCR-NH_2
QR	Ac-YGRKKRRQRRRGSGQYCR-NH_2

Note: uppercase letters represent *L* amino acids, and lowercase letters represent *D* amino acids.

Table S3. The HPLC purity and LRMS data of targeted peptidomimetics

compounds	LRMS			HPLC	
	ion form	calculated	observed	retention time/min	Purity
VTP-01	$[M+H]^+$	611.3	611.4	7.137	95.94%
VTP-02	$[M+H]^+$	612.3	612.4	7.373	95.69%
VTP-03	$[M+H]^+$	636.3	636.4	9.928	95.89%
VTP-04	$[M+H]^+$	650.3	650.3	8.770	97.88%
VTP-05	$[M+H]^+$	613.3	613.3	11.234	97.27%
VTP-06	$[M+H]^+$	637.3	637.9	14.605	95.78%
VTP-07	$[M+H]^+$	651.3	651.4	10.517	95.60%
VTP-08	$[M+H]^+$	638.3	638.3	11.113	95.39%
VTP-09	$[M+H]^+$	652.3	652.4	12.203	95.36%
VTP-10	$[M+H]^+$	653.3	653.4	12.000	96.08%
VTP-11	$[M+H]^+$	672.3	672.3	8.742	99.55%
VTP-12	$[M+H]^+$	686.3	686.4	10.136	98.52%
VTP-13	$[M+H]^+$	700.3	700.4	8.665	99.59%
VTP-14	$[M+H]^+$	712.3	712.3	10.915	99.25%
VTP-15	$[M+H]^+$	728.4	728.5	8.655	99.56%
VTP-16	$[M+H]^+$	754.4	754.5	8.646	99.36%
VTP-17	$[M+H]^+$	740.4	740.5	8.054	99.38%
VTP-18	$[M+H]^+$	768.4	768.6	8.336	98.74%
VTP-19	$[M+H]^+$	748.3	748.6	10.282	99.05%
VTP-20	$[M+H]^+$	762.3	762.5	8.532	96.95%
VTP-21	$[M+H]^+$	763.3	763.5	10.989	99.52%
VTP-22	$[M+H]^+$	762.3	762.3	8.504	99.51%
VTP-23	$[M+H]^+$	677.3	677.4	9.566	99.72%
VTP-24	$[M+H]^+$	722.3	722.5	8.073	98.21%
VTP-25	$[M+H]^+$	723.3	723.4	9.187	98.28%
VTP-26	$[M+H]^+$	724.3	724.4	9.313	98.19%
VTP-27	$[M+H]^+$	712.3	712.4	9.271	98.88%
VTP-28	$[M+H]^+$	730.3	730.4	8.629	99.00%
VTP-29	$[M+H]^+$	728.3	728.4	8.804	97.06%
VTP-30	$[M+H]^+$	702.3	702.3	9.303	99.07%
VTP-31	$[M+H]^+$	664.3	664.3	9.607	98.22%
VTP-32	$1/2[M+2H]^{2+}$	701.3	701.7	9.239	99.40%
VTP-33	$1/2[M+2H]^{2+}$	701.3	701.7	9.724	95.65%
VTP-34	$1/2[M+2H]^{2+}$	701.3	701.6	9.674	99.64%
VTP-35	$1/2[M+2H]^{2+}$	700.3	700.4	7.895	98.72%
VTP-36	$[M+H]^+$	1089.4	1089.9	8.742	96.98%
VTP-37	$1/2[M+2H]^{2+}$	410.2	410.4	8.111	97.67%
VTP-38	$[M+H]^+$	954.4	954.8	9.562	97.77%
VTP-39	$1/2[M+2H]^{2+}$	573.2	573.5	10.615	99.08%
VTP-40	$1/2[M+2H]^{2+}$	701.3	701.9	10.287	97.88%
VTP-41	$1/2[M+2H]^{2+}$	699.3	699.8	9.148	96.73%
VTP-42	$1/2[M+2H]^{2+}$	770.2	770.2	9.350	97.05%
VTP-43	$1/2[M+2H]^{2+}$	768.2	768.2	12.325	98.22%
VTP-44	$1/2[M+2H]^{2+}$	785.3	785.7	8.200	97.81%
VTP-45	$1/2[M+2H]^{2+}$	757.4	757.8	9.384	98.30%
VTP-46	$1/2[M+2H]^{2+}$	703.3	703.8	9.891	99.24%

VTP-47	1/2$[M+2H]^{2+}$	772.2	772.7	8.832	99.11%
VTP-48	1/2$[M+2H]^{2+}$	770.2	770.5	7.819	95.08%
FITC-VTP-32	1/2$[M+2H]^{2+}$	1069.4	1070.0	12.939	96.15%
PAL-VTP-32	1/2$[M+2H]^{2+}$	701.31	701.45	9.137	98.96%

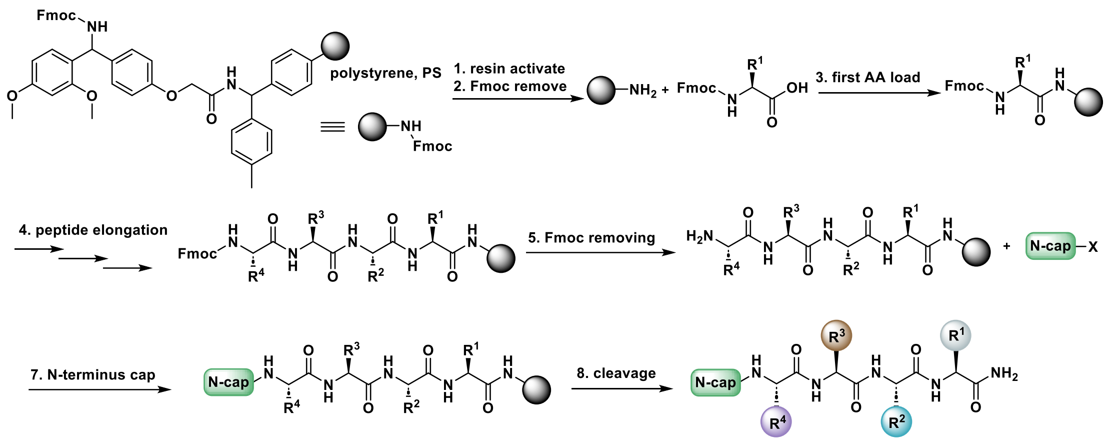

Scheme S1. The general routine of Rink Amide resin-based solid-phase peptide synthesis.

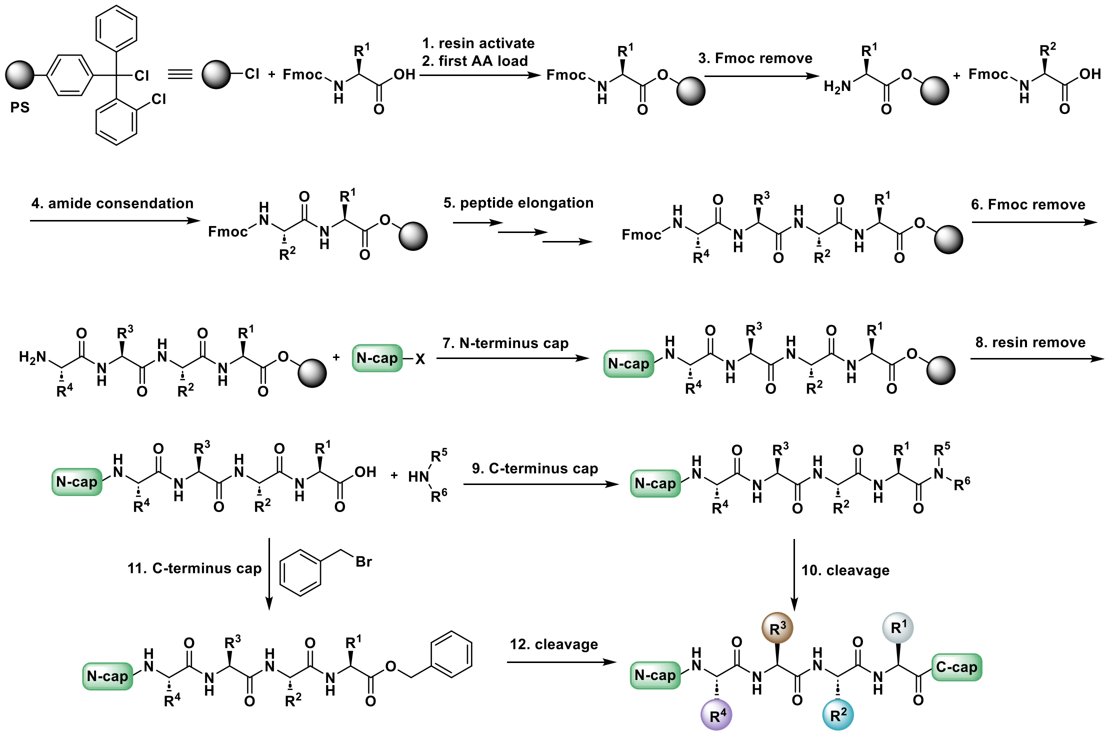

Scheme S2. The general routine of 2-CTC resin-based solid-phase peptide synthesis.

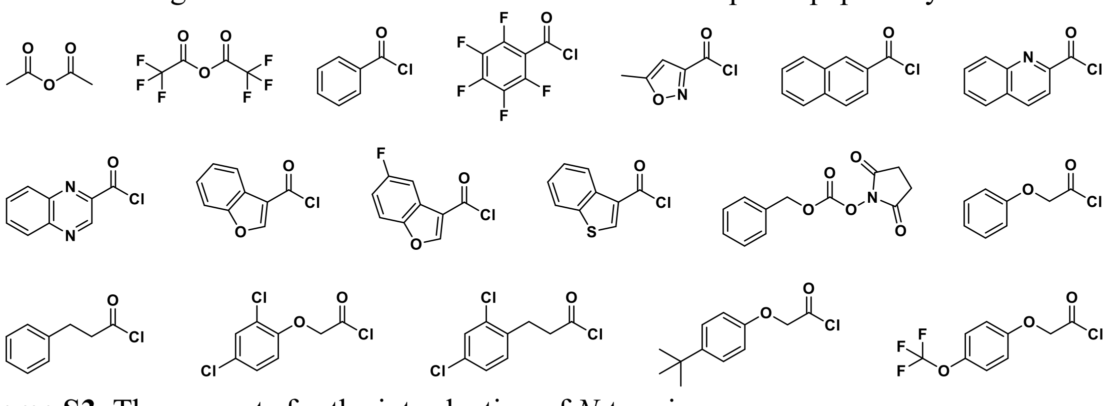

Scheme S3. The reagents for the introduction of *N*-terminus caps.

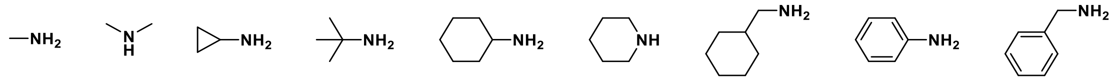

Scheme S4. The reagents for the introduction of *C*-terminus caps using the general procedure 1.

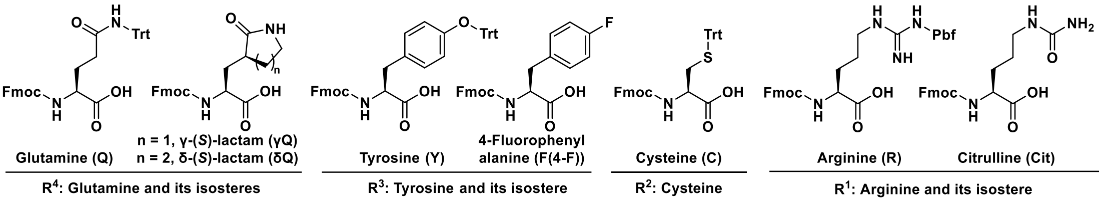

Scheme S5. Amino acids involved in the synthesis of targeted peptidomimetics.

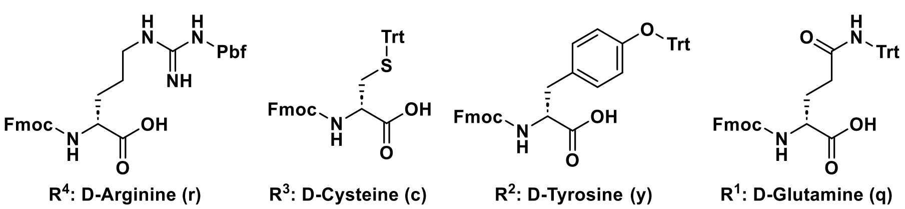

Scheme S6. Amino acids involved in the synthesis of VTP-34 and VTP-35.

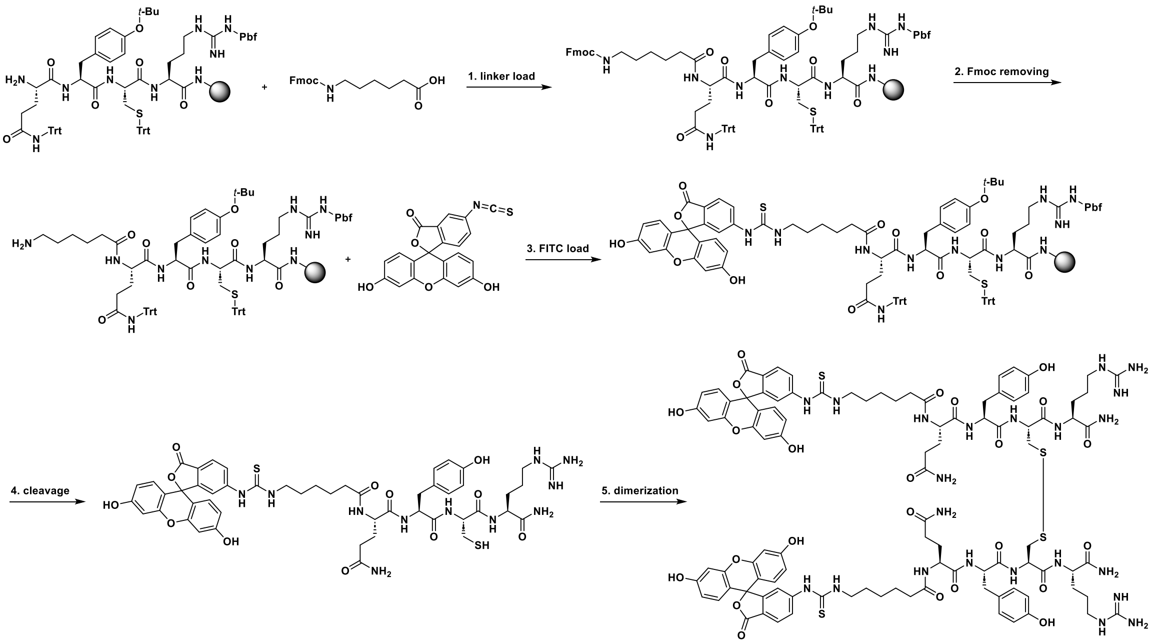

Scheme S7. The synthesis pathway of probe FITC-VTP-32.

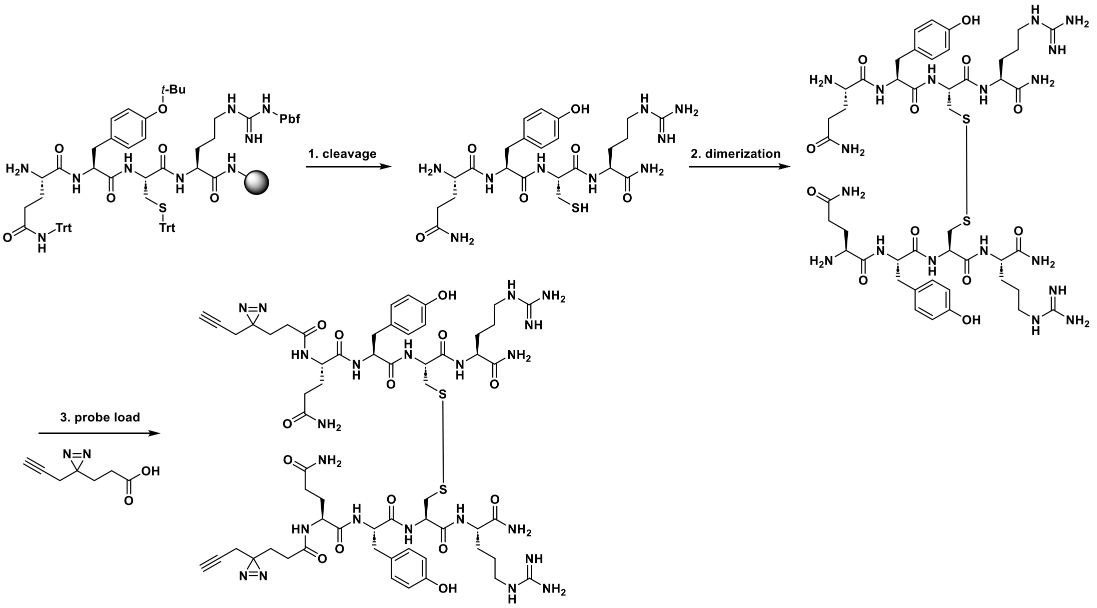

Scheme S8. The synthesis pathway of probe PAL-VTP-32.

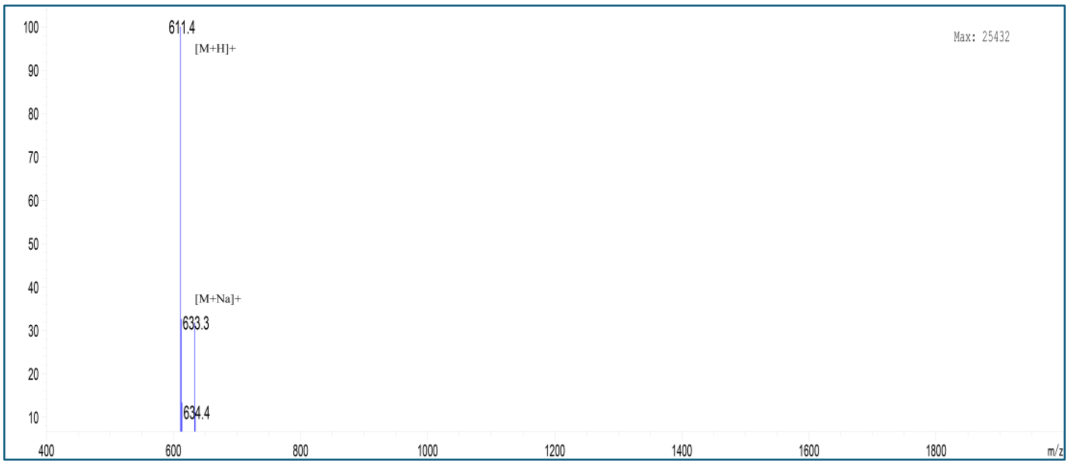

Column:	Gemini-NX 5µ C18 110A, 4.6*250mm
Solvent A	A: 0.1% Trifluoroacetic Acid in 100% Acetonitrile
Solvent B	B: 0.1% Trifluoroacetic Acid in 100% Water

Gradient:

	A	B
0.0min	5%	95%
25.0min	30%	70%
25.1min	100%	0%
30.0min	Stop	

Volume:	20µl
Wavelength:	220nm
Flow rate:	1.0ml/min

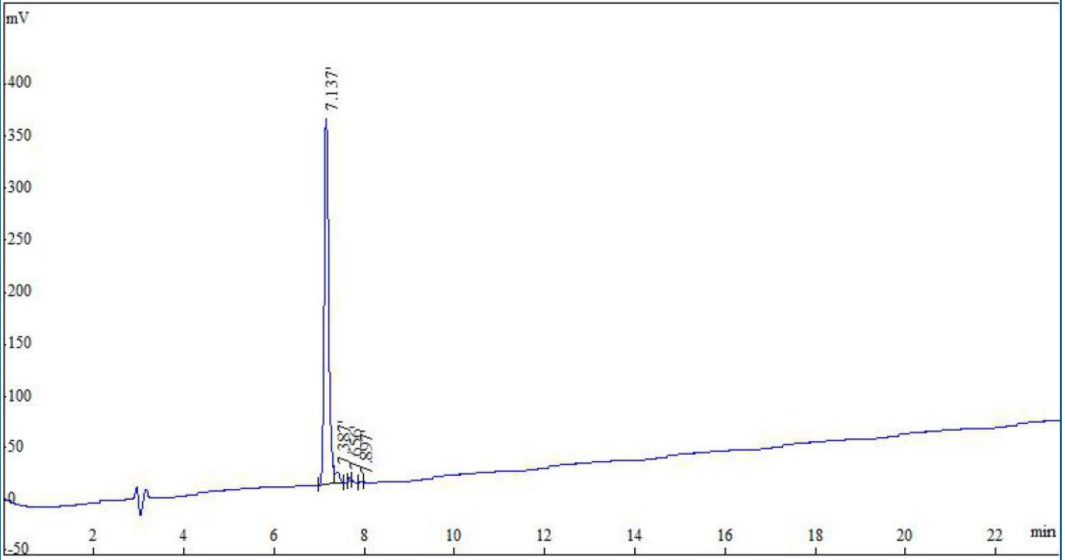

Rank	Time	Conc.	Area	Height
1	7.137	95.9366	2218058	352657
2	7.387	3.2966	76217	11419
3	7.656	0.5770	13340	3521
4	7.897	0.1898	4389	1211
Total		100	2312004	368808

The LRMS and HPLC purity data of VTP-01

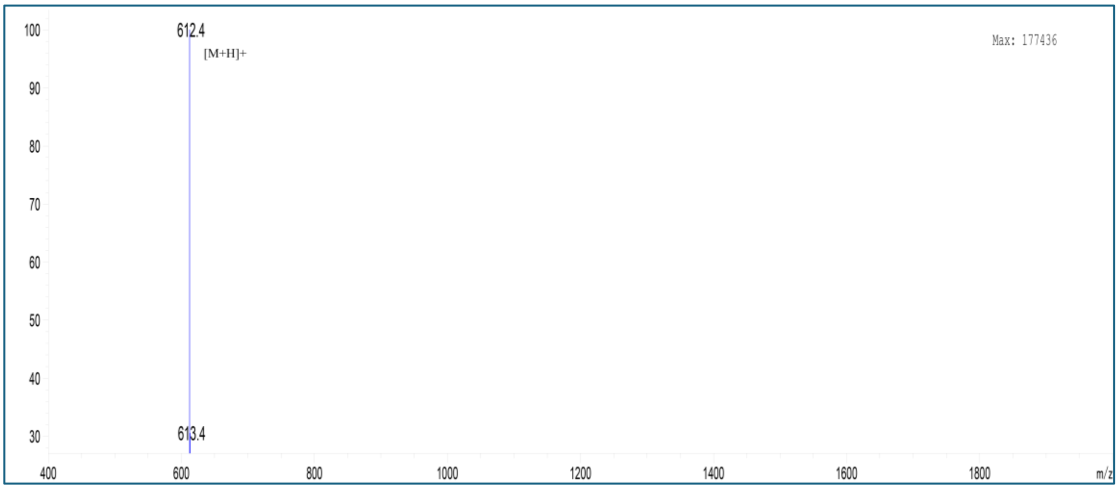

Column:	Gemini-NX 5μ C18 110A, 4.6*250mm
Solvent A	A: 0.1% Trifluoroacetic Acid in 100% Acetonitrile
Solvent B	B: 0.1% Trifluoroacetic Acid in 100% Water

Gradient:		A	B
	0.0min	15%	85%
	25.0min	40%	60%
	25.1min	100%	0%
	30.0min	Stop	

Volume:	20μl
Wavelength:	220nm
Flow rate:	1.0ml/min

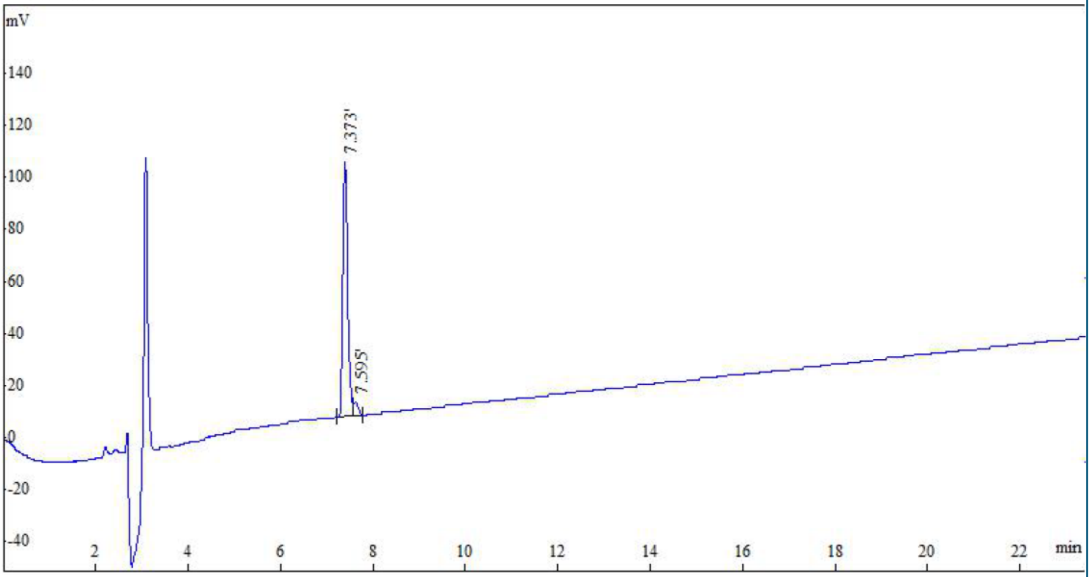

Rank	Time	Conc.	Area	Height
1	7.373	95.6946	663803	97337
2	7.595	4.3054	29865	4877
Total		100	693668	102214

The LRMS and HPLC purity data of VTP-02

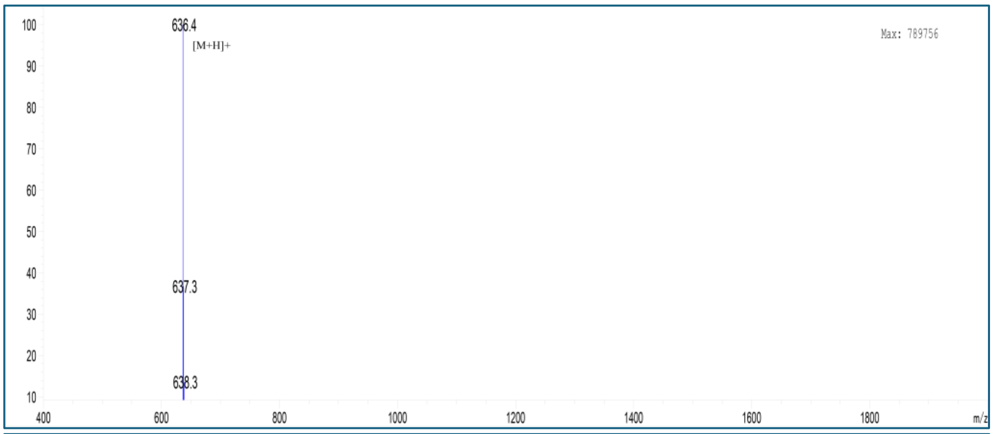

Column : 4.6×250mm,Sinochrom ODS-BP 5μm

Solvent A : 0.1% trifluoroacetic in 100% acetonitrile

Solvent B : 0.1% trifluoroacetic in 100% water

Gradient :

	A	B
0.01min	11%	89%
25min	36%	64%
25.1min	100%	0%
30min	STOP	

Flow rate : 1.0 mL/min

Wavelength : 220nm

Volume : 5ul

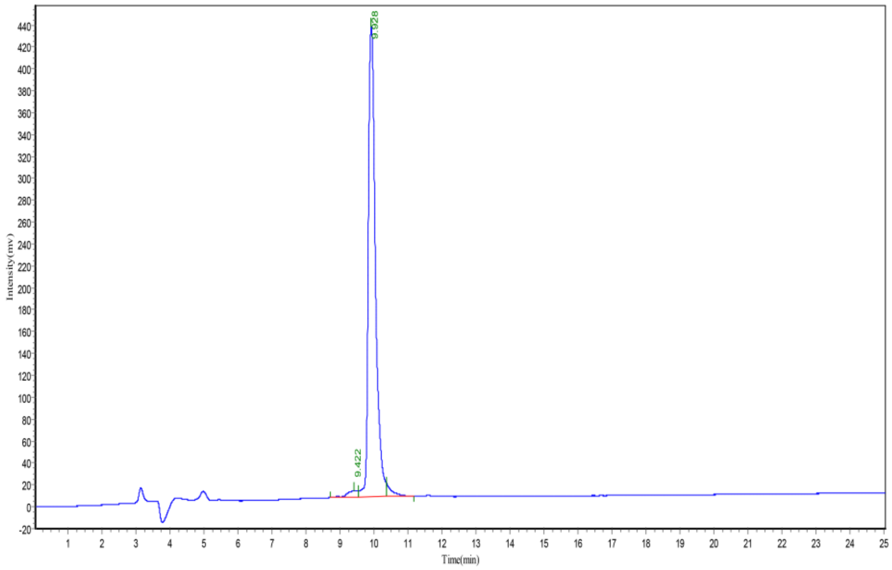

Peak No.	Ret Time	Height	Area	Conc..
1	9.422	6081.014	128272.836	1.9772
2	9.928	429264.813	6221038.500	95.8891
3	9.928	12943.729	138428.375	2.1337
Total				100.0000

The LRMS and HPLC purity data of VTP-03

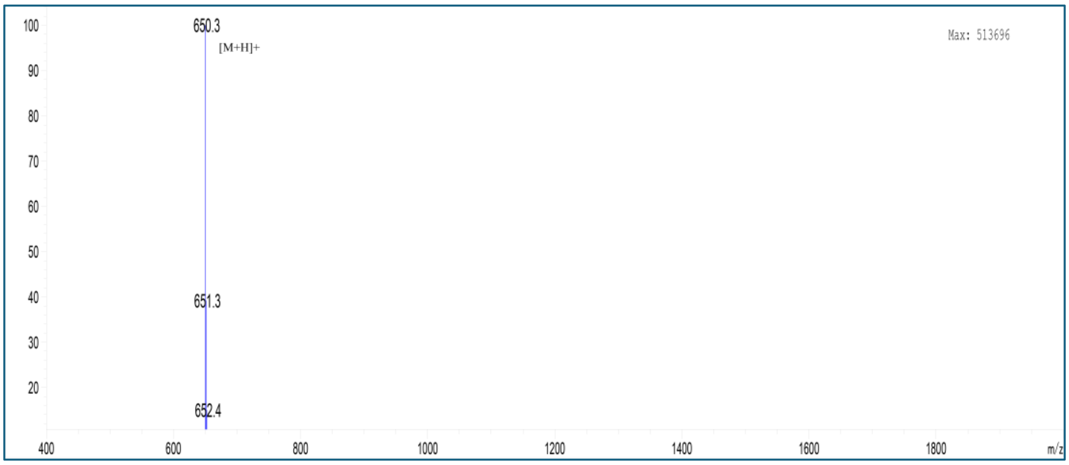

Column : 4.6×250mm,Sinochrom ODS-BP 5μm

Solvent A : 0.1% trifluoroacetic in 100% acetonitrile

Solvent B : 0.1% trifluoroacetic in 100% water

Gradient :

	A	B
0.01min	13%	87%
25min	38%	62%
25.1min	100%	0%
30min	STOP	

Flow rate : 1.0 mL/min

Wavelength : 220nm

Volume : 5ul

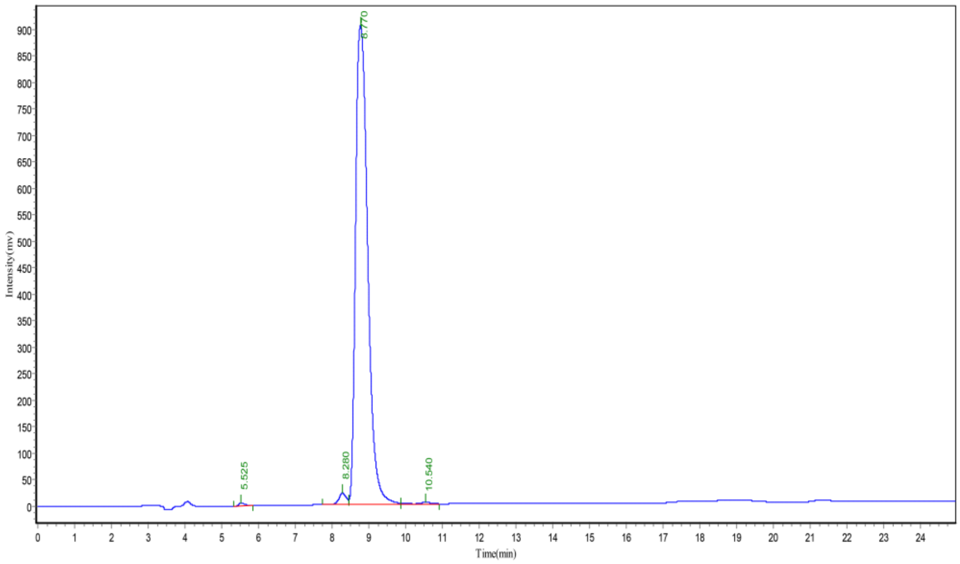

Peak No.	Ret Time	Height	Area	Conc..
1	5.525	5582.356	68246.203	0.3225
2	8.280	21557.426	305536.406	1.4438
3	8.770	903735.125	20713836.000	97.8827
4	10.540	3816.526	74287.430	0.3510
Total				100.0000

The LRMS and HPLC purity data of VTP-04

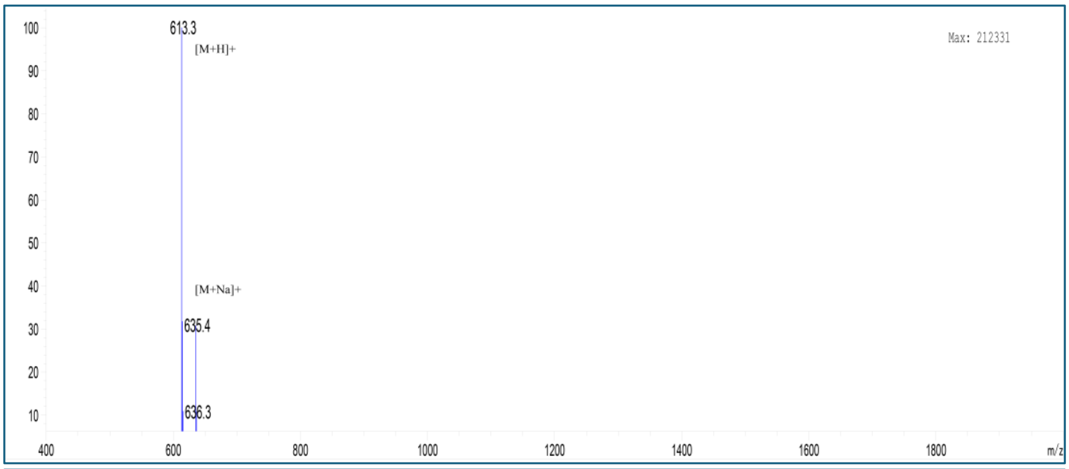

Column:	Gemini-NX 5μ C18 110A, 4.6*250mm
Solvent A	A: 0.1% Trifluoroacetic Acid in 100% Acetonitrile
Solvent B	B: 0.1% Trifluoroacetic Acid in 100% Water

Gradient:		A	B
	0.0min	15%	85%
	25.0min	40%	60%
	25.1min	100%	0%
	30.0min	Stop	

Volume:	20μl
Wavelength:	220nm
Flow rate:	1.0ml/min

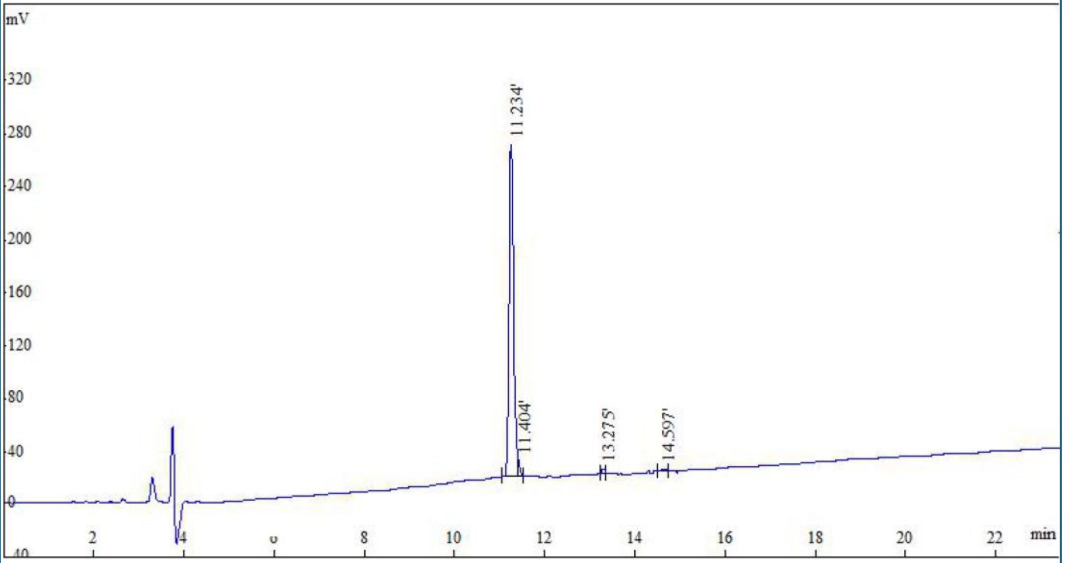

Rank	Time	Conc.	Area	Height
1	11.234	97.2681	1586193	248967
2	11.404	1.4252	23241	10372
3	13.275	0.5781	9427	2239
4	14.597	0.7286	11882	1726
Total		100	1630743	263304

The LRMS and HPLC purity data of VTP-05

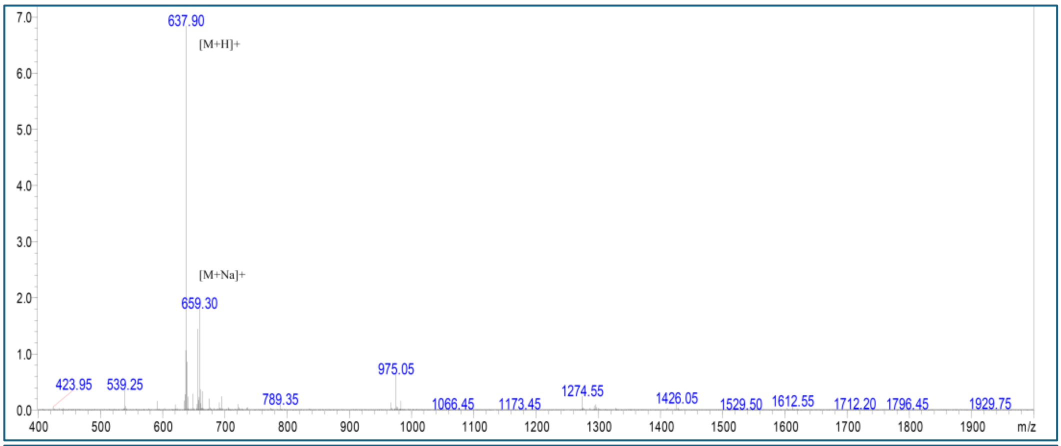

Column : 4.6×250mm,Venusil MP C18-5

Solvent A : 0.1% trifluoroacetic in 100% acetonitrile

Solvent B : 0.1% trifluoroacetic in 100% water

Gradient :	A	B
0.01min	10%	90%
25min	35%	65%
25.1min	100%	0%
30min	STOP	

Flow rate : 1.0 mL/min

Wavelength : 220nm

Volume : 5ul

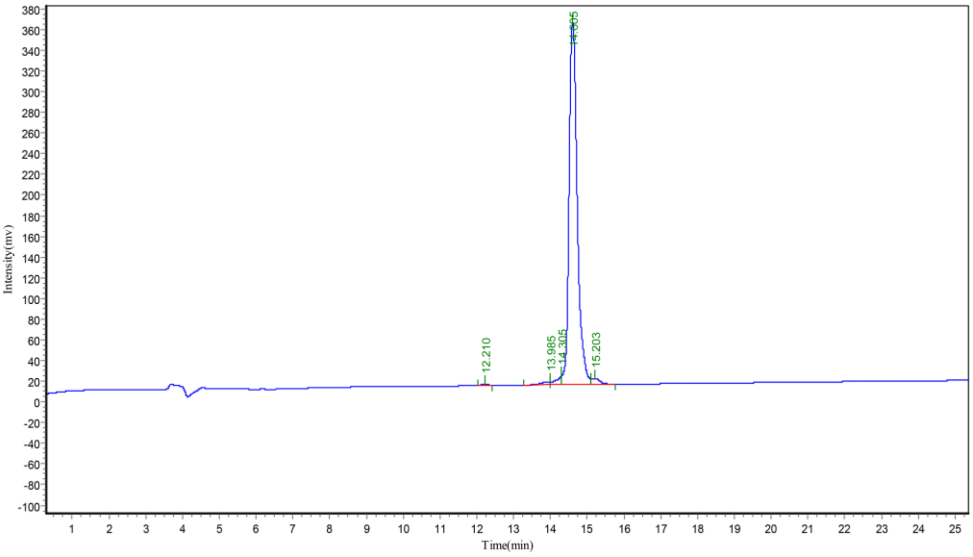

Peak No.	Ret Time	Height	Area	Conc..
1	12.210	1591.390	18691.512	0.3311
2	13.985	2499.778	46033.207	0.8155
3	14.305	8346.845	87180.852	1.5445
4	14.605	350549.688	5406162.500	95.7781
5	15.203	5603.912	86398.969	1.5307
Total				100.0000

The LRMS and HPLC purity data of VTP-06

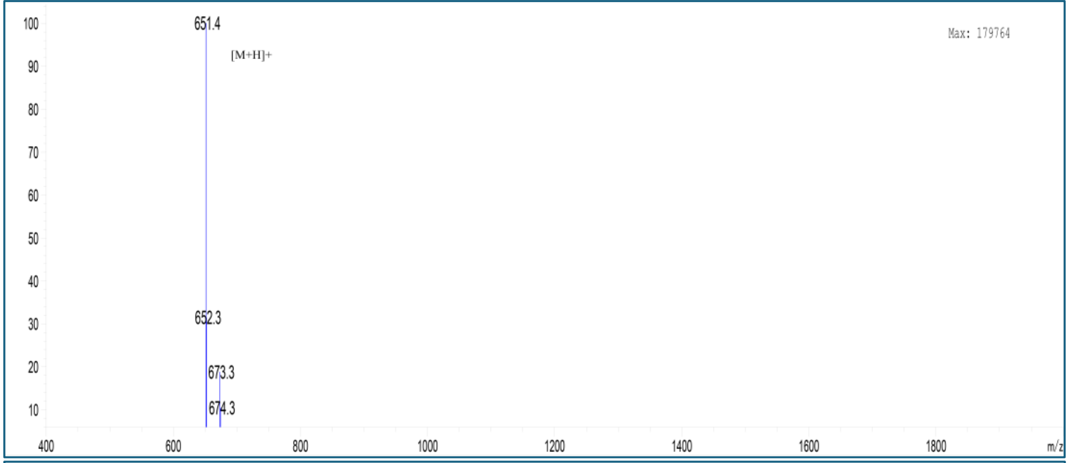

Column : 4.6×250mm,Sinochrom ODS-BP 5μm
Solvent A : 0.1% trifluoroacetic in 100% acetonitrile
Solvent B : 0.1% trifluoroacetic in 100% water

Gradient :	A	B
0.01min	7%	93%
25min	32%	68%
25.1min	100%	0%
30min	STOP	

Flow rate : 1.0 mL/min
Wavelength : 220nm
Volume : 5ul

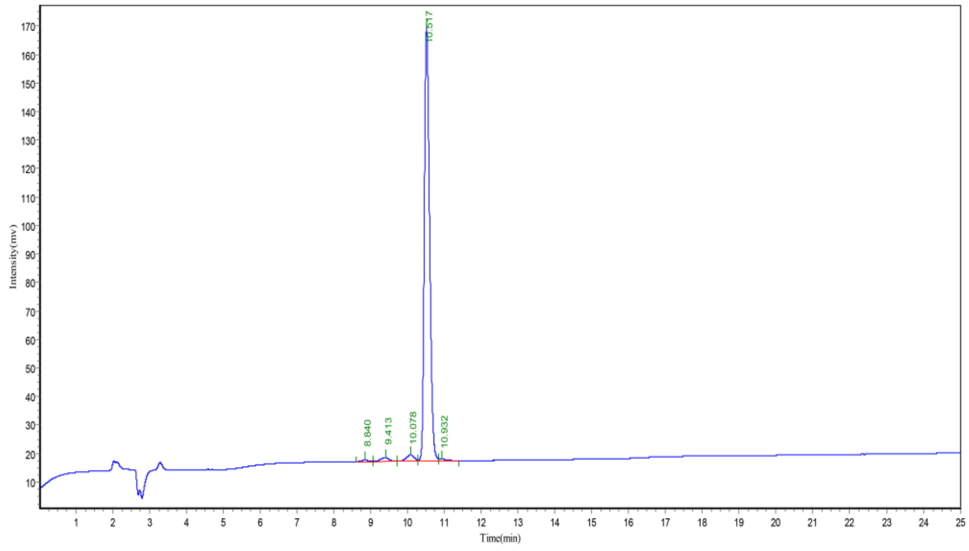

Peak No.	Ret Time	Height	Area	Conc..
1	8.840	546.259	7053.557	0.4500
2	9.413	1109.891	20130.617	1.2844
3	10.078	2203.833	33113.836	2.1127
4	10.517	152482.078	1498446.000	95.6033
5	10.932	686.581	8614.752	0.5496
Total				100.0000

The LRMS and HPLC purity data of VTP-07

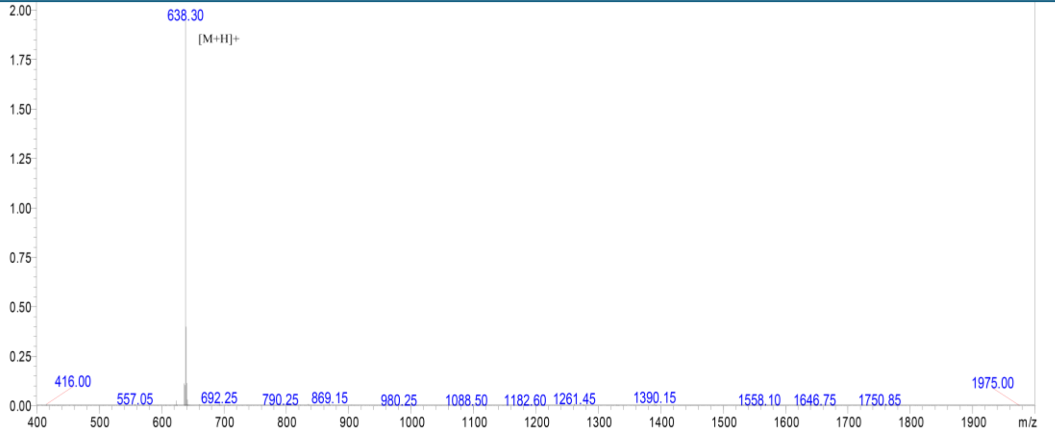

Column : 4.6×250mm,Venusil MP C18-5

Solvent A : 0.1% trifluoroacetic in 100% acetonitrile

Solvent B : 0.1% trifluoroacetic in 100% water

Gradient :	A	B
0.01min	14%	86%
25min	39%	61%
25.1min	100%	0%
30min	STOP	

Flow rate : 1.0 mL/min

Wavelength : 220nm

Volume : 5ul

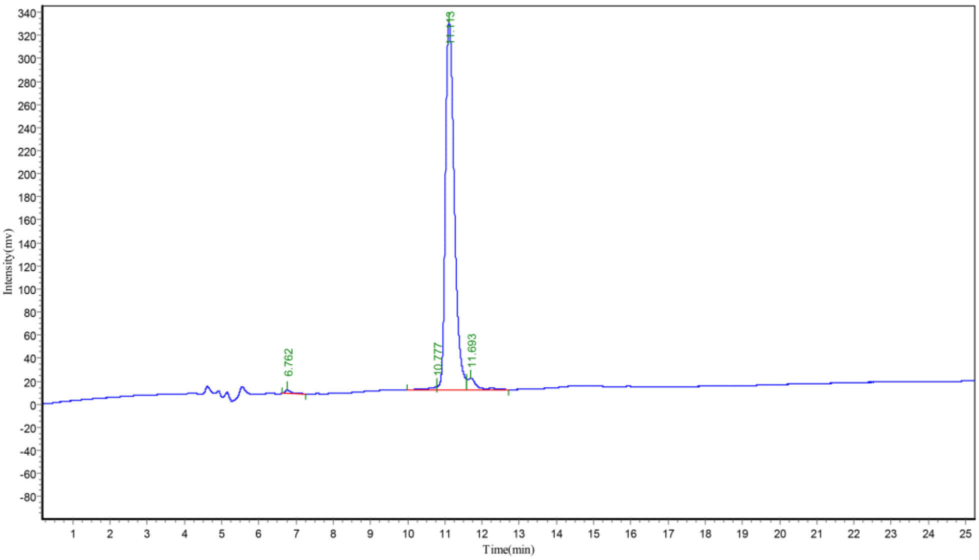

Peak No.	Ret Time	Height	Area	Conc..
1	6.762	2821.587	38838.402	0.6981
2	10.777	2659.510	39192.105	0.7044
3	11.113	319387.750	5307172.000	95.3926
4	11.693	10039.430	178305.141	3.2049
Total				100.0000

The LRMS and HPLC purity data of VTP-08

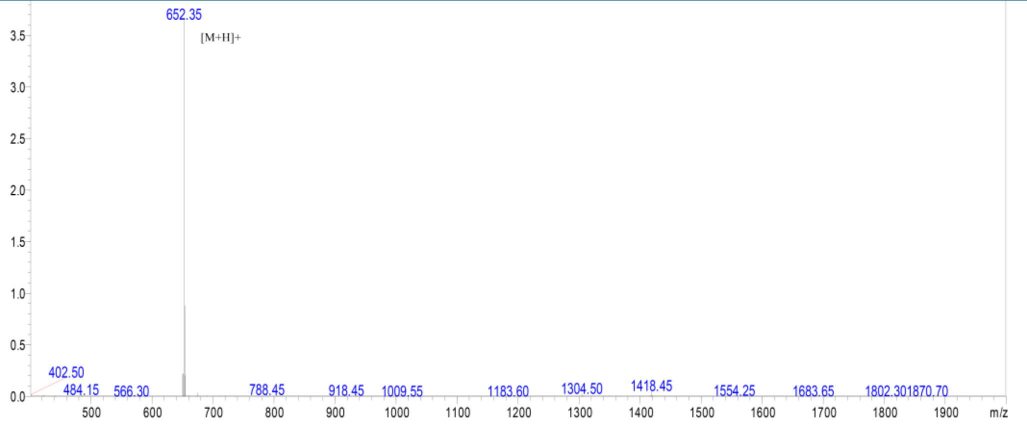

Column : 4.6×250mm,Venusil MP C18-5
Solvent A : 0.1% trifluoroacetic in 100% acetonitrile
Solvent B : 0.1% trifluoroacetic in 100% water
Gradient :

	A	B
0.01min	13%	87%
25min	38%	62%
25.1min	100%	0%
30min	STOP	

Flow rate : 1.0 mL/min
Wavelength : 220nm
Volume : 5ul

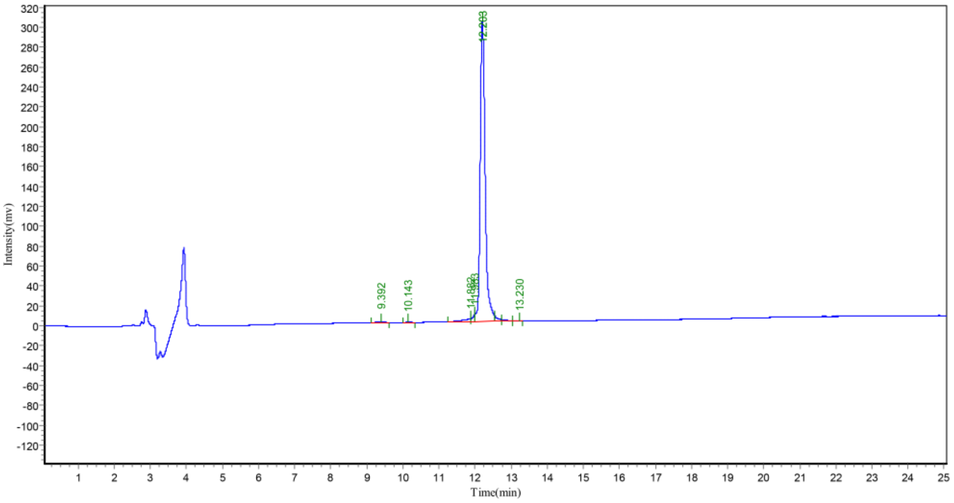

Peak No.	Ret Time	Height	Area	Conc..
1	9.392	1110.888	16473.598	0.5438
2	10.143	541.076	4164.806	0.1375
3	11.882	2577.802	40978.402	1.3526
4	11.983	6316.345	26566.482	0.8769
5	12.203	302683.000	2888851.500	95.3560
6	12.203	5657.930	33936.246	1.1202
7	12.203	1785.608	16144.671	0.5329
8	13.230	224.955	2426.701	0.0801
Total				100.0000

The LRMS and HPLC purity data of VTP-09

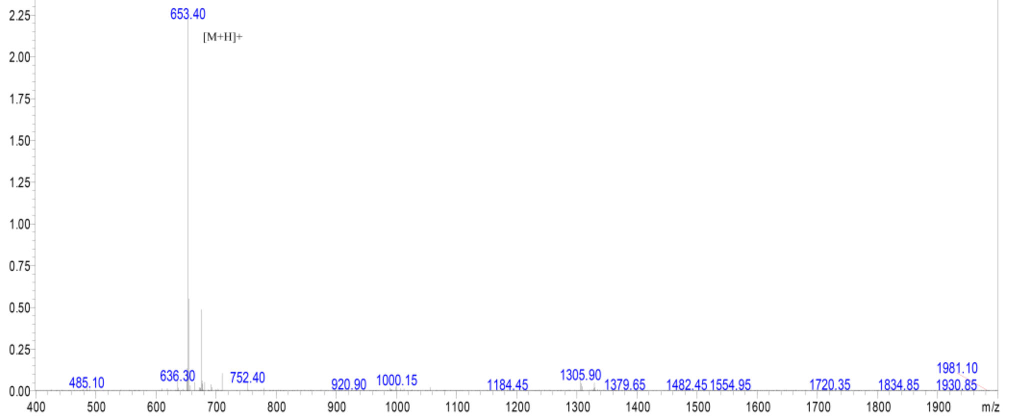

Column : 4.6×250mm, Kromasil 100-5-C18

Solvent A : 0.1% trifluoroacetic in 100% acetonitrile

Solvent B : 0.1% trifluoroacetic in 100% water

Gradient :

	A	B
0.01min	13%	87%
25min	38%	62%
25.1min	100%	0%
30min	STOP	

Flow rate : 1.0 mL/min

Wavelength : 220nm

Volume : 5ul

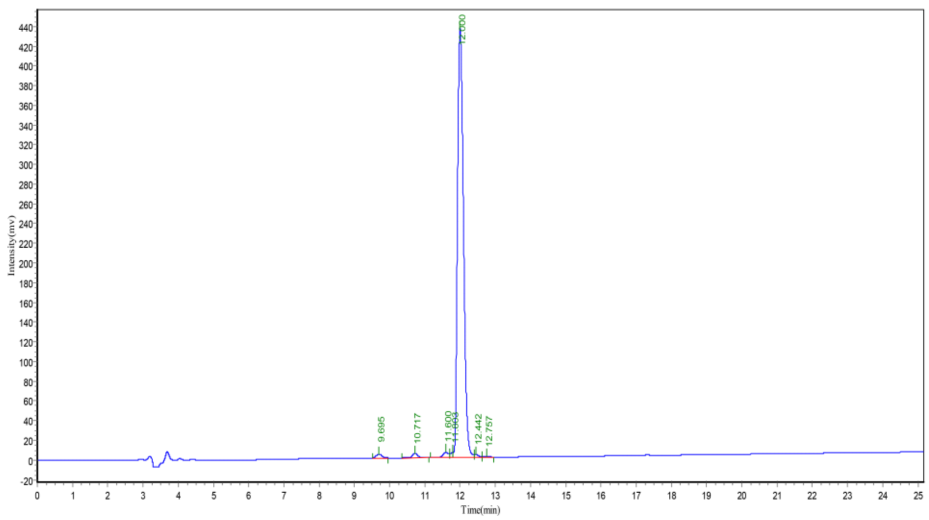

Peak No.	Ret Time	Height	Area	Conc..
1	9.695	3765.600	43752.004	0.8174
2	10.717	4611.042	49135.438	0.9179
3	11.600	5364.941	57330.969	1.0710
4	11.803	5161.043	27922.746	0.5216
5	12.000	435048.469	5143203.500	96.0843
6	12.442	2413.104	21967.184	0.4104
7	12.757	952.762	9493.451	0.1774
Total				100.0000

The LRMS and HPLC purity data of VTP-10

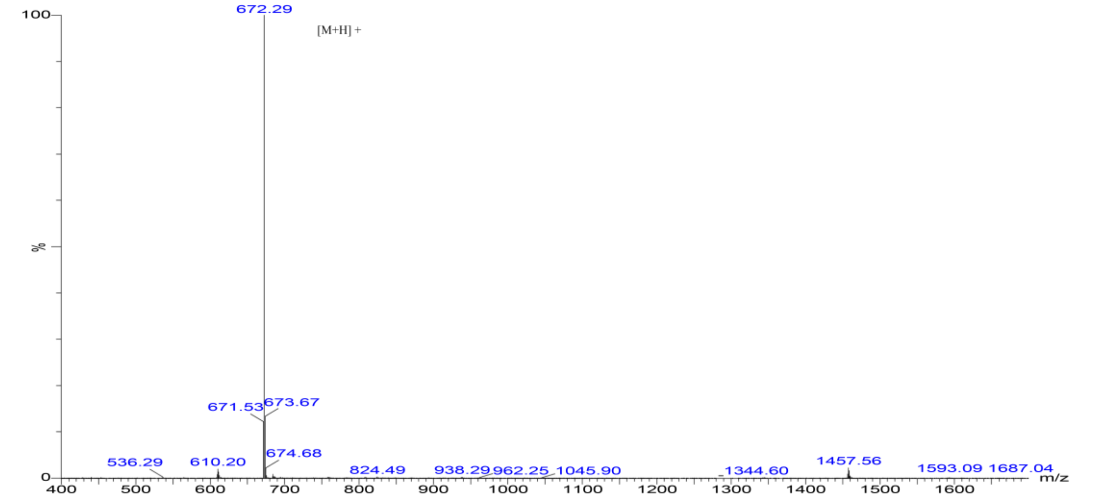

Description: Mobile phase:Buffer A:0.1%TFA in H2O
Buffer B:0.1%TFA in ACN
Gradient:16-26% B in 20min , Flow rate:1.0ml/min
Column:Unitary C18,4.6*150mm,5um 100A

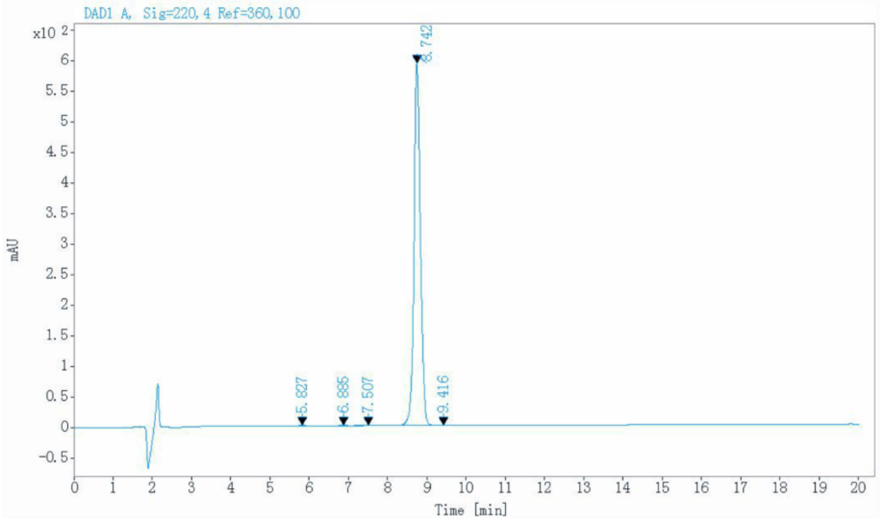

Signal: DAD1 A, Sig=220,4 Ref=360,100

RT[min]	Type	Width[min]	Area	Height	Area%	Name
5.827	BB	0.1527	11.1872	1.1182	0.1670	
6.885	BV	0.2085	5.3838	0.3642	0.0803	
7.507	VB	0.2099	6.7691	0.4541	0.1010	
8.742	MF	0.1876	6670.6113	592.7564	99.5511	
9.416	FM	0.2856	6.7386	0.3932	0.1006	
		Sum	6700.6900			

The LRMS and HPLC purity data of VTP-11

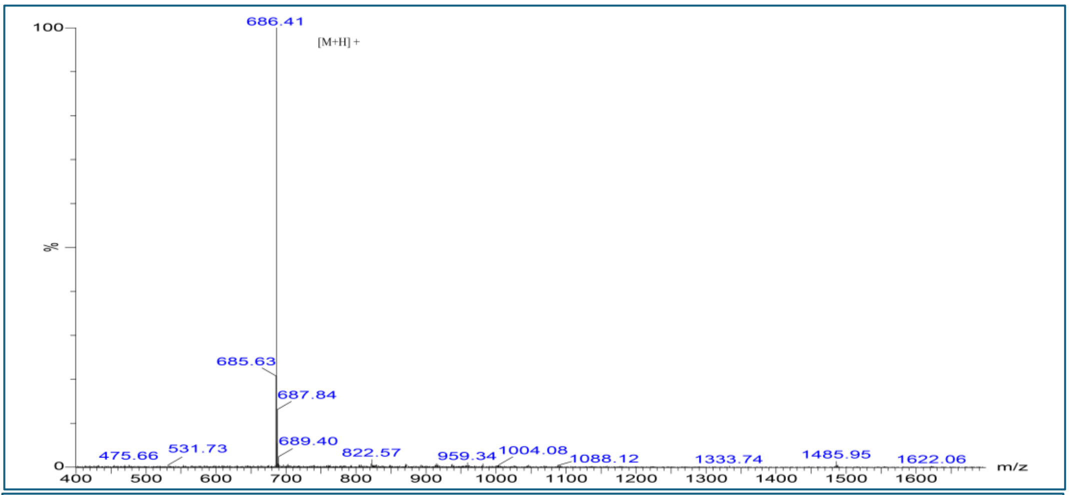

Description: Mobile phase:Buffer A:0.1%TFA in H2O
Buffer B:0.1%TFA in ACN
Gradient:16-26% B in 20min, Flow rate:1.0ml/min
Column:Unitary C18,4.6*150mm,5um 100A

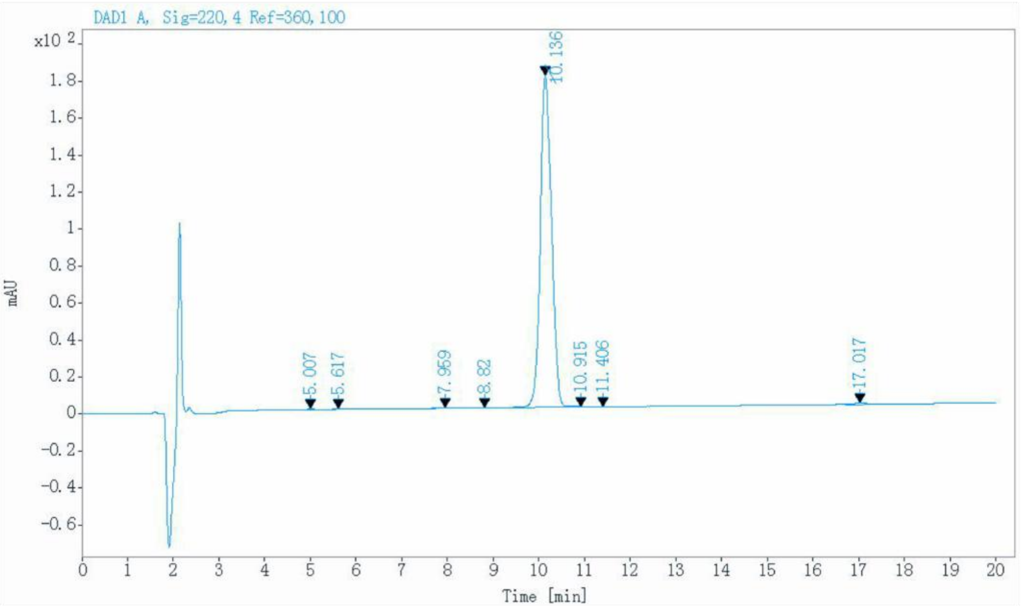

Signal: DAD1 A, Sig=220,4 Ref=360,100

RT[min]	Type	Width[min]	Area	Height	Area%	Name
5.007	VB	0.1845	4.4971	0.3845	0.1454	
5.617	BB	0.2134	3.6750	0.2592	0.1188	
7.959	BB	0.5241	11.7710	0.3038	0.3805	
8.820	BB	0.2509	3.2963	0.2150	0.1066	
10.136	MF	0.2824	3047.7185	179.8802	98.5191	
10.915	MF	0.2792	6.0079	0.3586	0.1942	
11.406	FM	0.2654	1.8847	0.1183	0.0609	
17.017	FM	0.2810	14.6792	0.8708	0.4745	
		Sum	3093.5298			

The LRMS and HPLC purity data of VTP-12

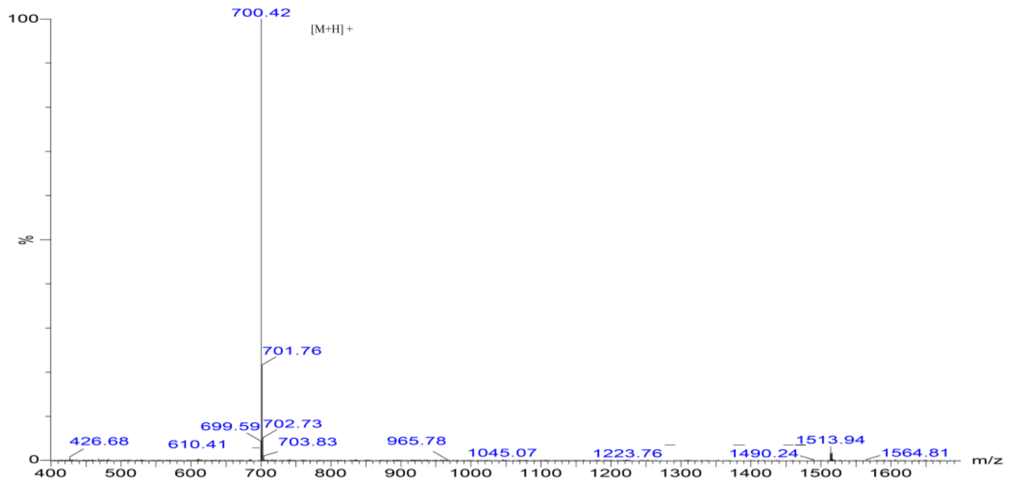

Description: Mobile phase:Buffer A:0.1%TFA in H2O
Buffer B:0.1%TFA in ACN
Gradient:18-28% B in 20min, Flow rate:1.0ml/min
Column:Unitary C18,4.6*150mm,5um 100A

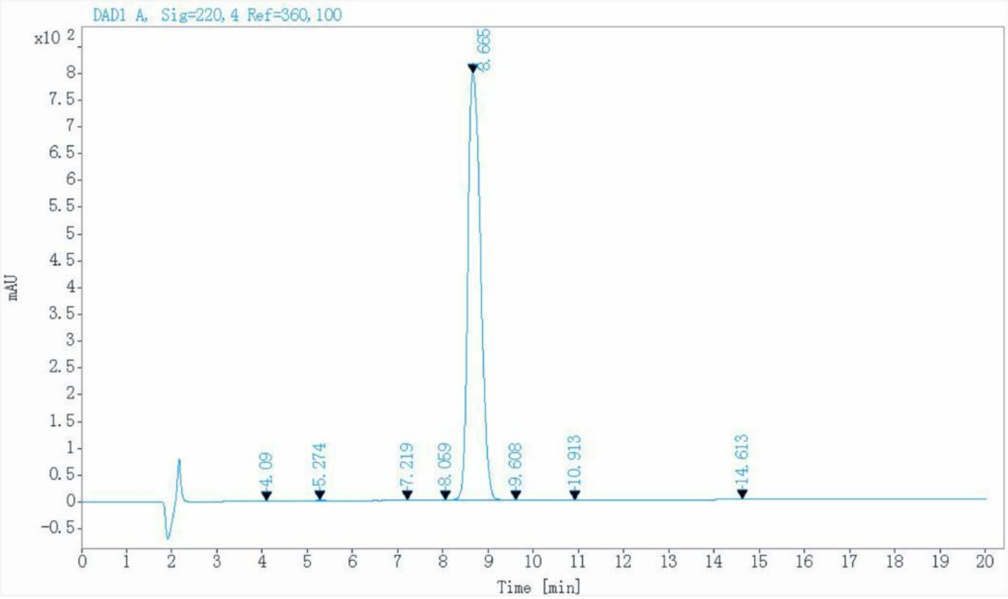

Signal: DAD1 A, Sig=220,4 Ref=360,100

RT[min]	Type	Width[min]	Area	Height	Area%	Name
4.090	BB	0.1576	4.1549	0.4121	0.0267	
5.274	BB	0.1963	15.2525	1.1684	0.0980	
7.219	BV E	0.2210	5.8642	0.3865	0.0377	
8.059	VV E	0.3394	17.9173	0.6712	0.1151	
8.665	VV R	0.3107	15500.4541	797.8574	99.5899	
9.608	VB E	0.2079	3.8212	0.2720	0.0246	
10.913	BB	0.2532	5.9330	0.3308	0.0381	
14.613	BB	0.3178	10.8812	0.5339	0.0699	
		Sum	15564.2784			

The LRMS and HPLC purity data of VTP-13

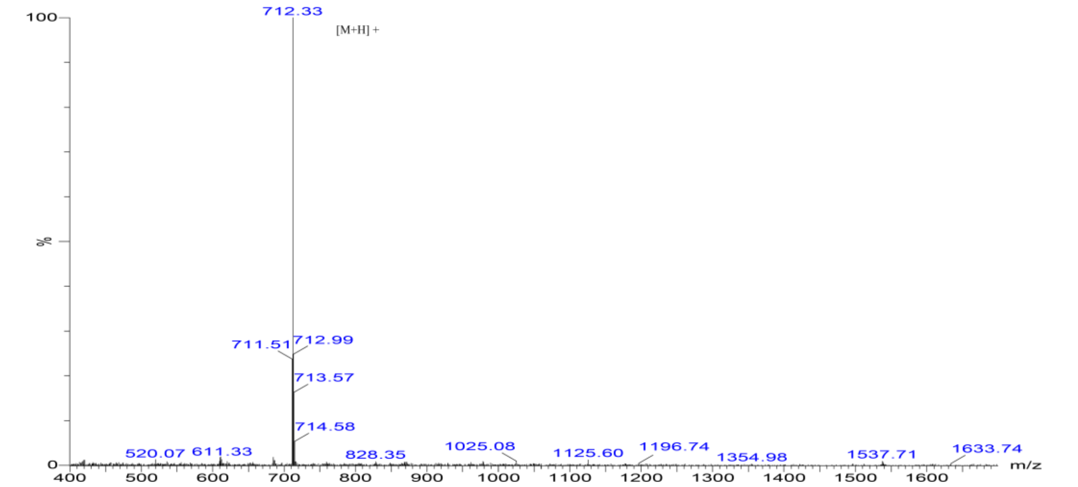

Description: Mobile phase:Buffer A:0.1%TFA in H2O
Buffer B:0.1%TFA in ACN
Gradient:18-28% B in 20min, Flow rate:1.0ml/min
Column:Unitary C18,4.6*150mm,5um 100A

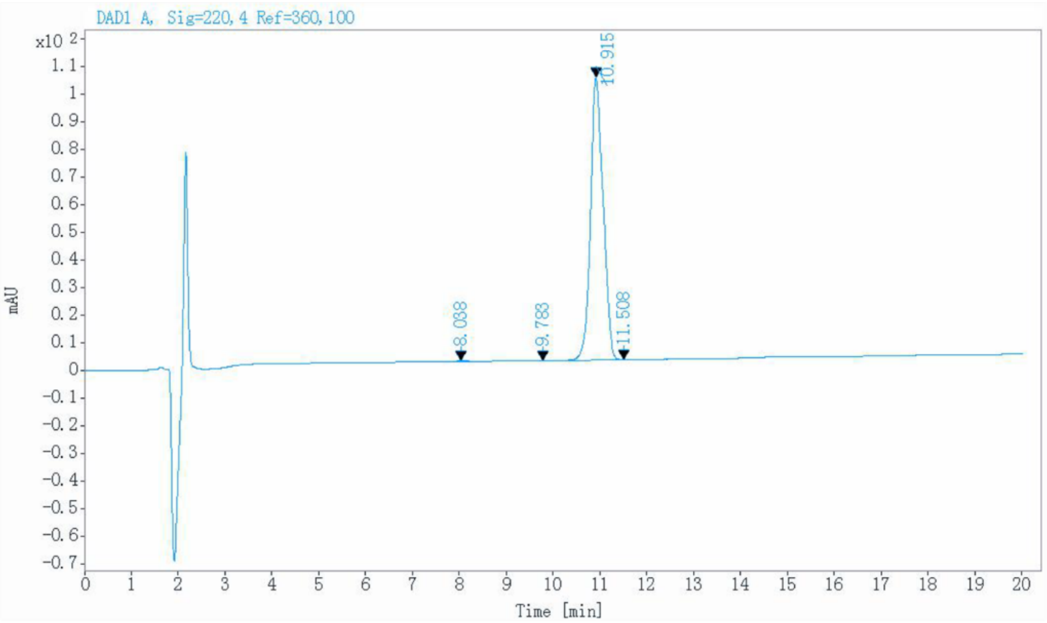

Signal: DAD1 A, Sig=220,4 Ref=360,100

RT[min]	Type	Width[min]	Area	Height	Area%	Name
8.038	FM	0.3422	5.4968	0.2677	0.2845	
9.783	MF	0.3473	4.5653	0.1715	0.2363	
10.915	FM	0.3124	1917.9462	102.3217	99.2541	
11.508	FM	0.3465	4.3519	0.2093	0.2252	
		Sum	1932.3601			

The LRMS and HPLC purity data of VTP-14

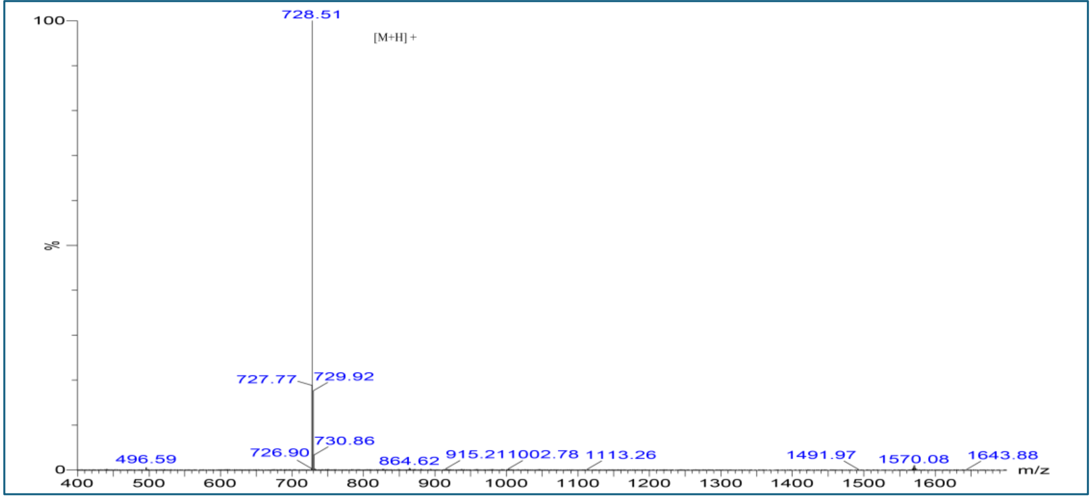

Description: Mobile phase:Buffer A:0.1%TFA in H2O
Buffer B:0.1%TFA in ACN
Gradient:24-34% B in 20min , Flow rate:1.0ml/min
Column:Unitary C18,4.6*150mm,5um 100A

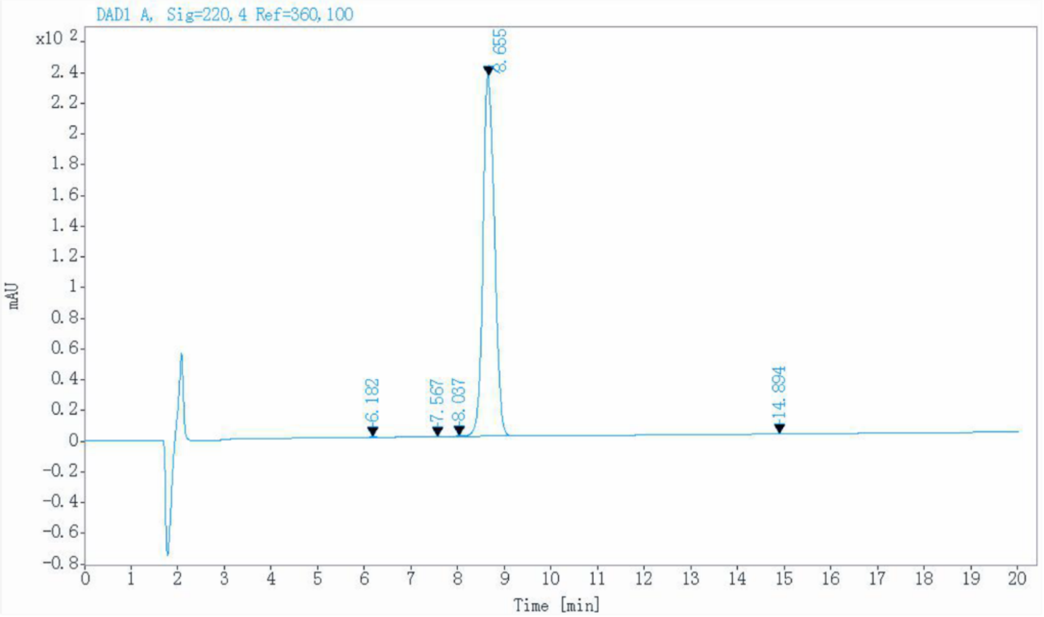

Signal: DAD1 A, Sig=220,4 Ref=360,100

RT[min]	Type	Width[min]	Area	Height	Area%	Name
6.182	BB	0.2559	3.5855	0.2274	0.0890	
7.567	BV E	0.2382	3.0703	0.2056	0.0762	
8.037	VV E	0.2227	6.8208	0.4172	0.1693	
8.655	VB R	0.2790	4010.2795	235.1000	99.5620	
14.894	BB	0.2606	4.1674	0.2473	0.1035	
		Sum	4027.9236			

The LRMS and HPLC purity data of VTP-15

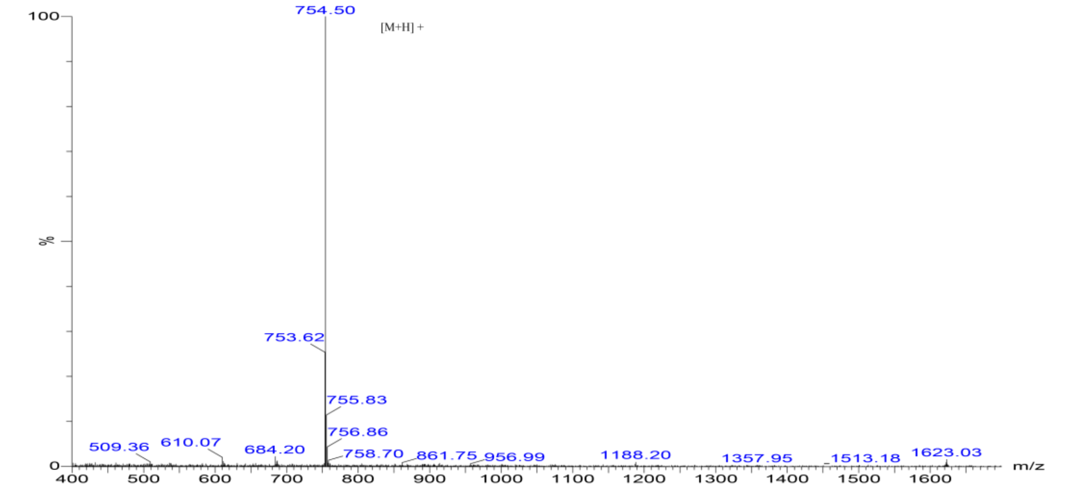

Description: Mobile phase:Buffer A:0.1%TFA in H2O
Buffer B:0.1%TFA in ACN
Gradient:27-37% B in 20min, Flow rate:1.0ml/min
Column:Unitary C18,4.6*150mm,5um 100A

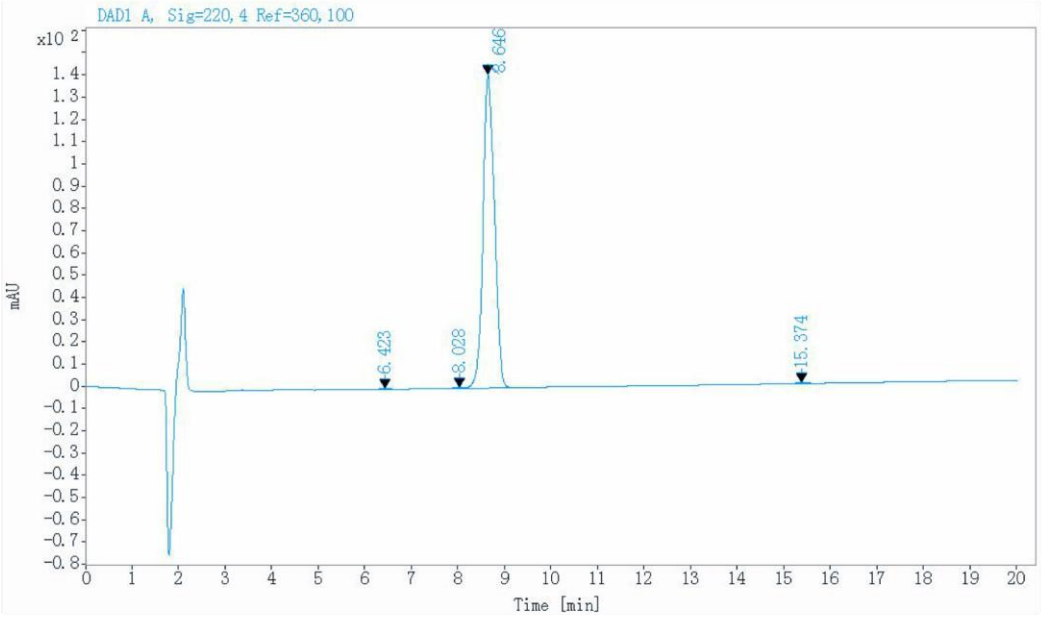

Signal: DAD1 A, Sig=220,4 Ref=360,100

RT[min]	Type	Width[min]	Area	Height	Area%	Name
6.423	BB	0.2329	5.0900	0.3516	0.2115	
8.028	MF	0.3170	3.9178	0.2060	0.1628	
8.646	FM	0.2826	2391.6567	141.0670	99.3597	
15.374	FM	0.3093	6.4043	0.3451	0.2661	
		Sum	2407.0689			

The LRMS and HPLC purity data of VTP-16

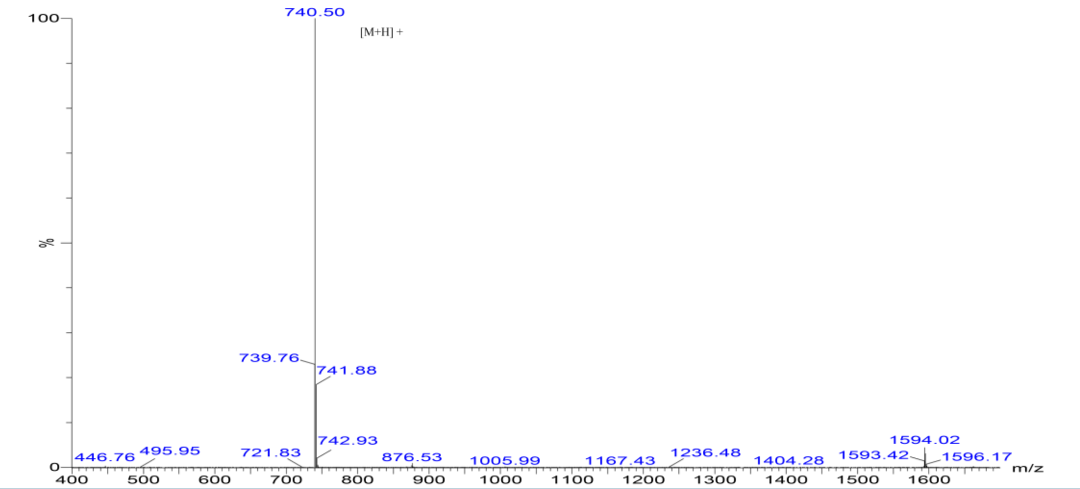

Description: Mobile phase:Buffer A:0.1%TFA in H2O
Buffer B:0.1%TFA in ACN
Gradient:23-33% B in 20min , Flow rate:1.0ml/min
Column:Unitary C18,4.6*150mm, 5um 100A

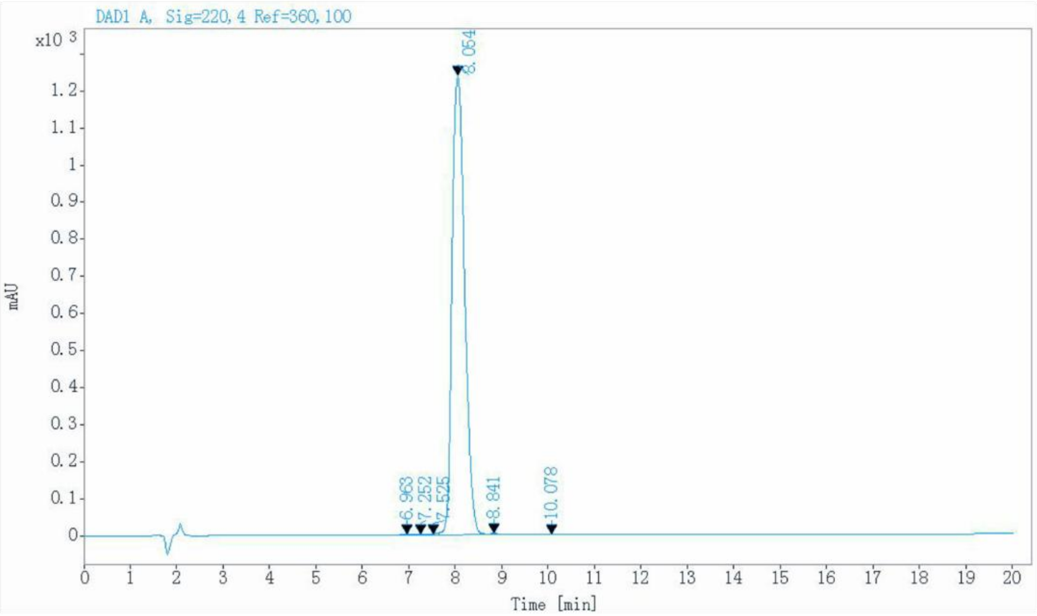

Signal: DAD1 A, Sig=220,4 Ref=360,100

RT[min]	Type	Width[min]	Area	Height	Area%	Name
6.963	BV	0.2314	12.7463	0.8476	0.0547	
7.252	MF	0.2263	16.5939	1.2223	0.0712	
7.525	MF	0.1310	41.5307	5.2824	0.1781	
8.054	MF	0.3117	23174.9180	1239.3436	99.3774	
8.841	MF	0.4010	63.7715	2.6505	0.2735	
10.078	FM	0.3227	10.5517	0.5449	0.0452	
		Sum	23320.1121			

The LRMS and HPLC purity data of VTP-17

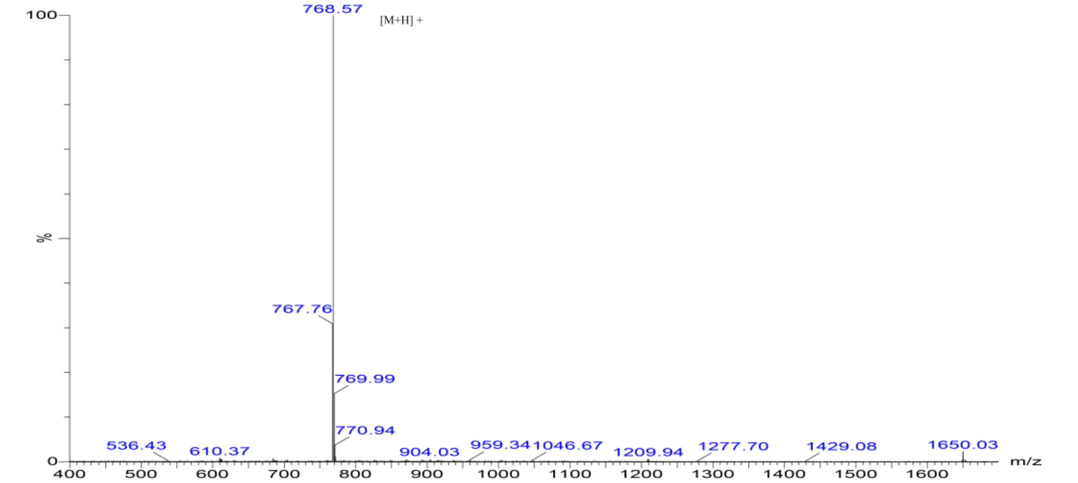

Description: Mobile phase:Buffer A:0.1%TFA in H2O
Buffer B:0.1%TFA in ACN
Gradient:30-40% B in 20min, Flow rate:1.0ml/min
Column:Unitary C18, 4.6*150mm, 5um 100A

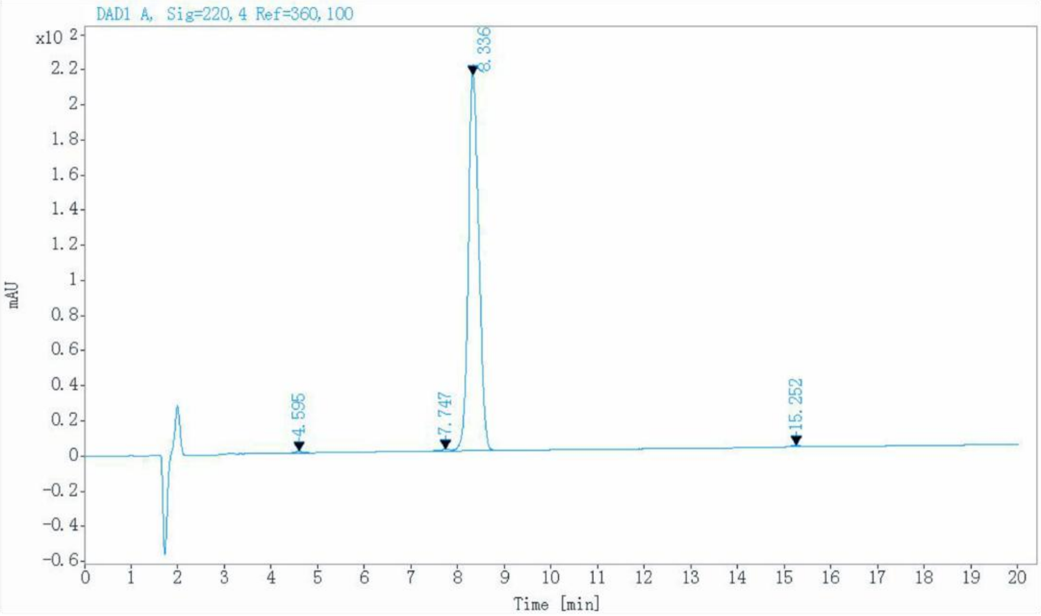

Signal: DAD1 A, Sig=220,4 Ref=360,100

RT[min]	Type	Width[min]	Area	Height	Area%	Name
4.595	BB	0.1791	10.1758	0.9057	0.3001	
7.747	BV E	0.2683	16.2270	0.8594	0.4785	
8.336	VB R	0.2467	3348.2952	213.7985	98.7373	
15.252	BB	0.2609	16.4174	0.9727	0.4841	
		Sum	3391.1153			

The LRMS and HPLC purity data of VTP-18

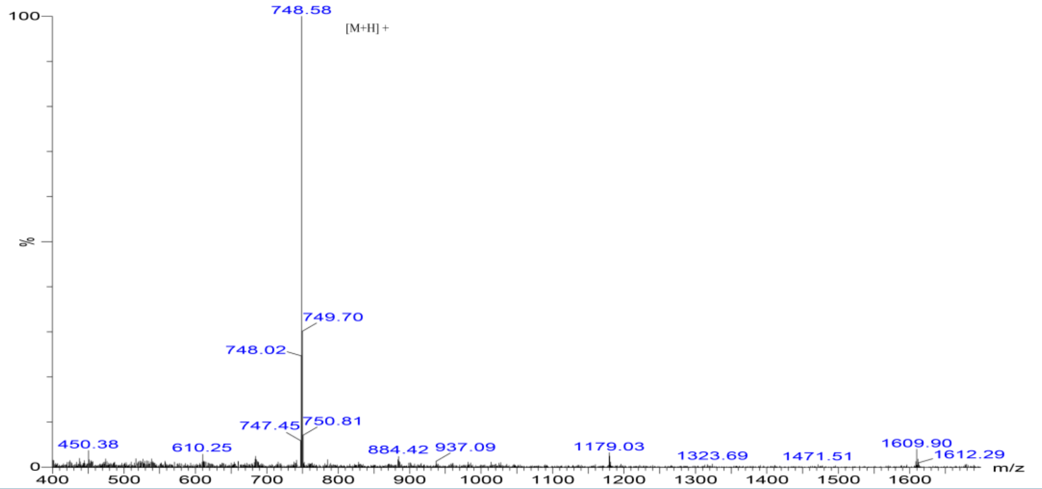

Description: Mobile phase:Buffer A:0.1%TFA in H2O
Buffer B:0.1%TFA in ACN
Gradient:26-36% B in 20min, Flow rate:1.0ml/min
Column:Unitary C18,4.6*150mm,5um 100A

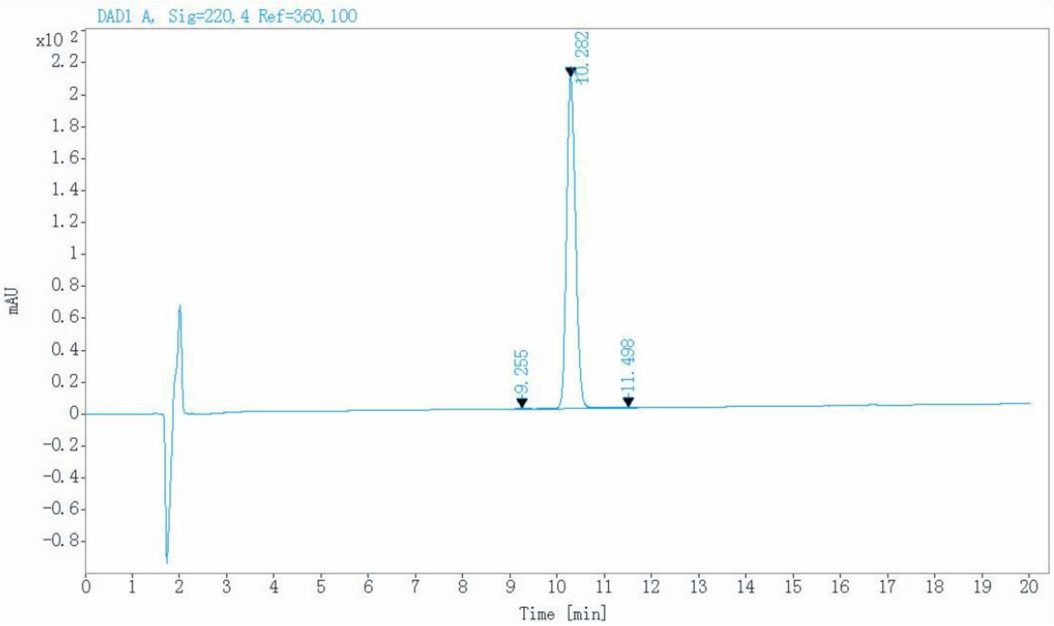

Signal: DAD1 A, Sig=220,4 Ref=360,100

RT[min]	Type	Width[min]	Area	Height	Area%	Name
9.255	BV	0.2115	7.8653	0.5612	0.2858	
10.282	VV R	0.2052	2725.6990	207.6193	99.0521	
11.498	VV E	0.7865	18.2189	0.2817	0.6621	
		Sum	2751.7832			

The LRMS and HPLC purity data of VTP-19

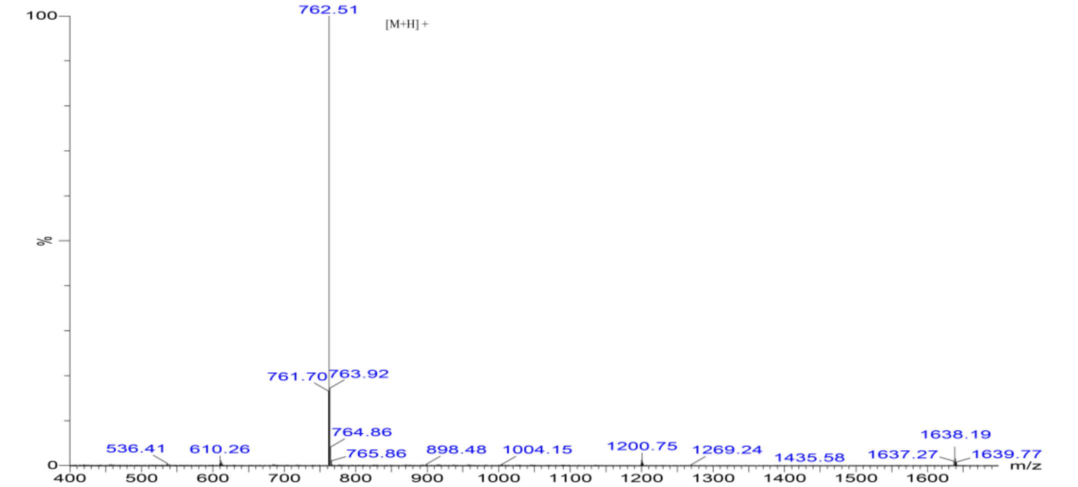

Description: Mobile phase:Buffer A:0.1%TFA in H2O
Buffer B:0.1%TFA in ACN
Gradient:26-36% B in 20min, Flow rate:1.0ml/min
Column:Unitary C18,4.6*150mm,5um 100A

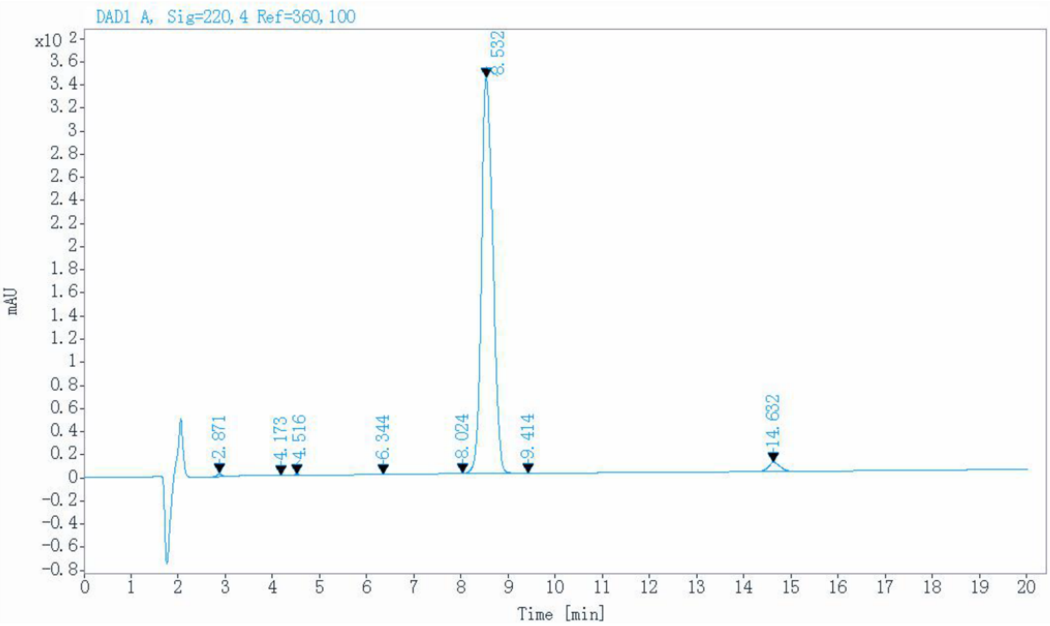

Signal: DAD1 A, Sig=220,4 Ref=360,100

RT[min]	Type	Width[min]	Area	Height	Area%	Name
2.871	BB	0.1074	17.5581	2.4548	0.2948	
4.173	BV	0.2255	7.4123	0.4423	0.1245	
4.516	VB	0.1549	6.7471	0.6304	0.1133	
6.344	VV R	0.2378	5.9794	0.3390	0.1004	
8.024	MF	0.1712	3.8343	0.3732	0.0644	
8.532	MF	0.2807	5773.4771	342.8300	96.9469	
9.414	FM	0.3579	2.7633	0.1287	0.0464	
14.632	FM	0.2901	137.5278	7.9005	2.3093	
		Sum	5955.2994			

The LRMS and HPLC purity data of VTP-20

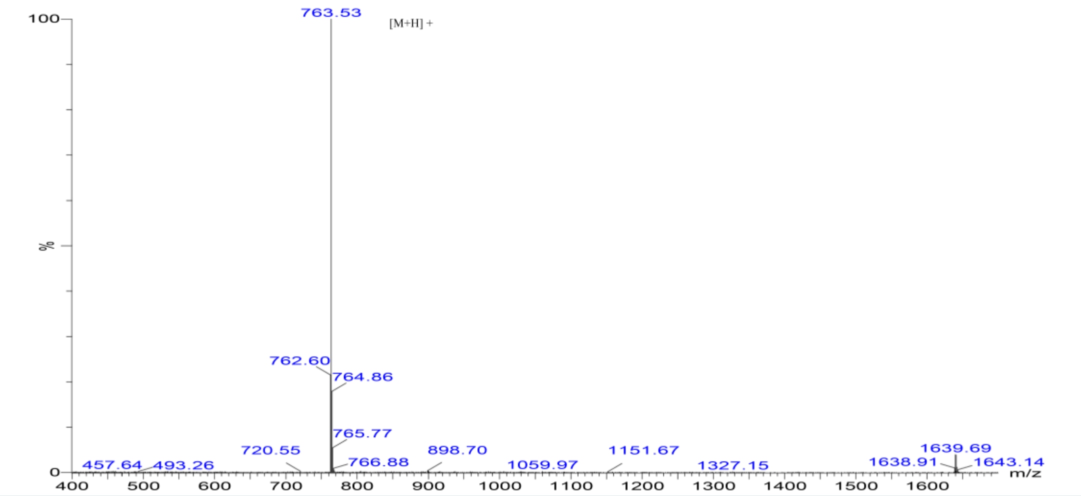

Description: Mobile phase:Buffer A:0.1%TFA in H2O
Buffer B:0.1%TFA in ACN
Gradient:28-38% B in 20min, Flow rate:1.0ml/min
Column:Unitary C18, 4.6*150mm, 5um 100A

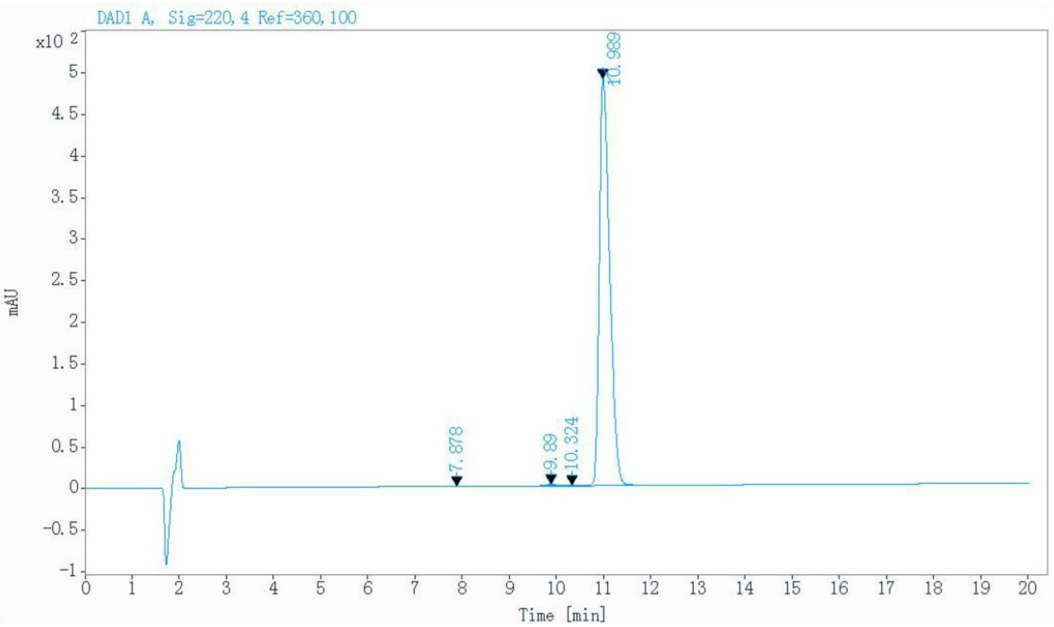

Signal: DAD1 A, Sig=220, 4 Ref=360, 100

RT[min]	Type	Width[min]	Area	Height	Area%	Name
7.878	BV	0.1866	2.9638	0.2569	0.0395	
9.890	BV E	0.1946	21.9466	1.6998	0.2928	
10.324	VV E	0.2252	10.8697	0.7155	0.1450	
10.989	VB R	0.2339	7458.8159	489.2209	99.5226	
		Sum	7494.5960			

The LRMS and HPLC purity data of VTP-21

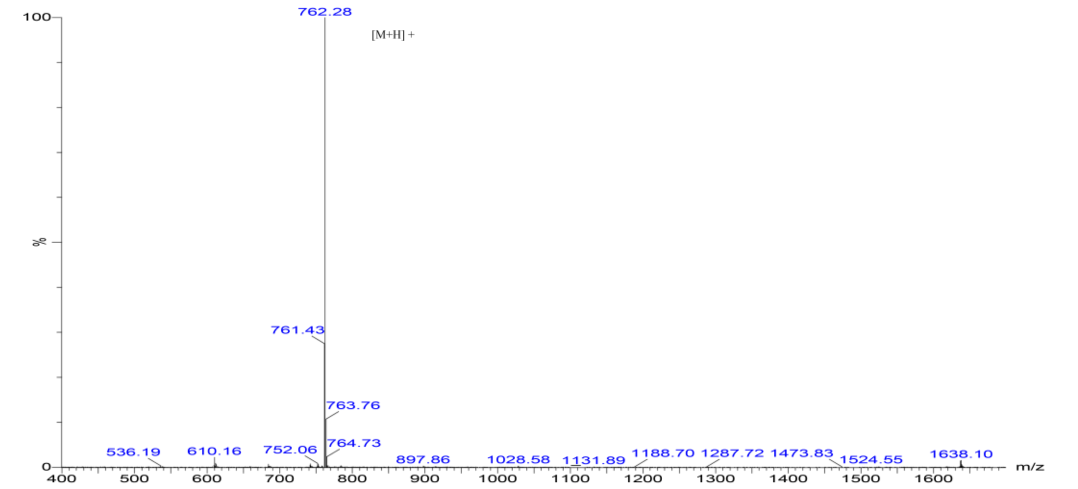

Description: Mobile phase:Buffer A:0.1%TFA in H2O
Buffer B:0.1%TFA in ACN
Gradient:21-31% B in 20min , Flow rate:1.0ml/min
Column:Unitary C18, 4.6*150mm, 5um 100A

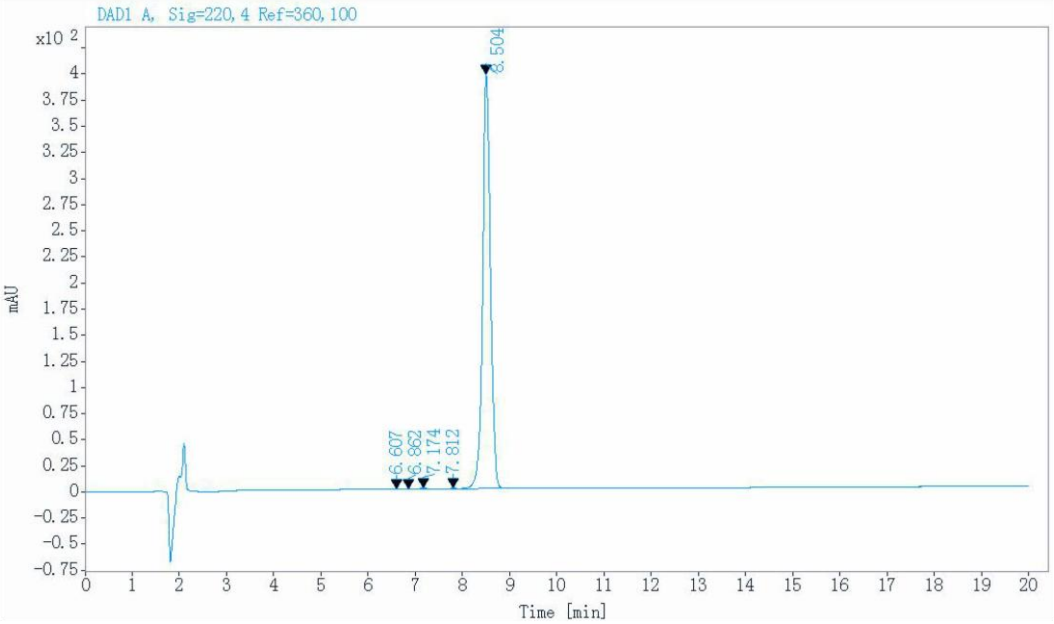

Signal: DAD1 A, Sig=220,4 Ref=360,100

RT[min]	Type	Width[min]	Area	Height	Area%	Name
6.607	BV	0.1530	5.5086	0.5491	0.1199	
6.862	VV	0.1433	4.4980	0.4892	0.0979	
7.174	VB	0.1444	7.5082	0.8082	0.1634	
7.812	BV E	0.1878	4.8066	0.3794	0.1046	
8.504	VB R	0.1748	4573.0977	395.7067	99.5143	
		Sum	4595.4190			

The LRMS and HPLC purity data of VTP-22

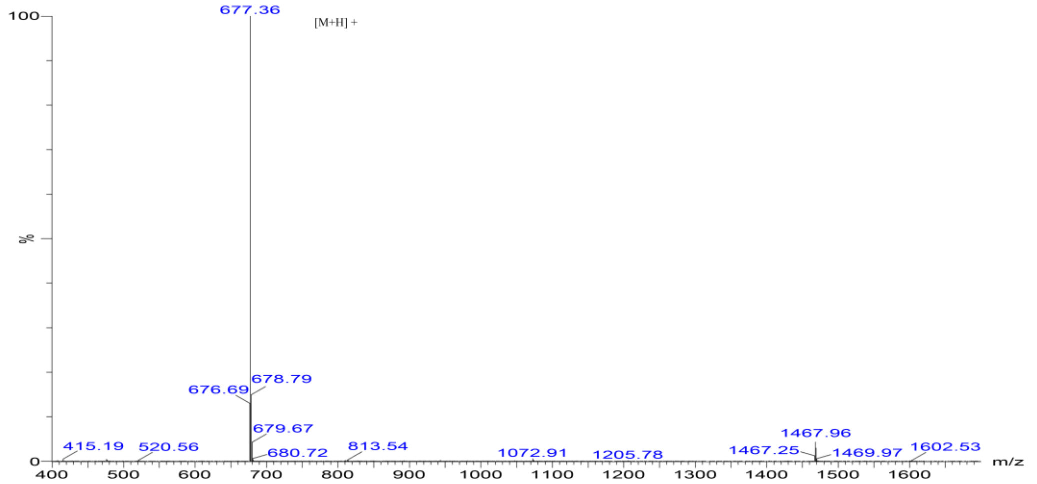

Description: Mobile phase:Buffer A:0.1%TFA in H2O
Buffer B:0.1%TFA in ACN
Gradient:13-23% B in 20min , Flow rate:1.0ml/min
Column:Unitary C18,4.6*150mm,5um 100A

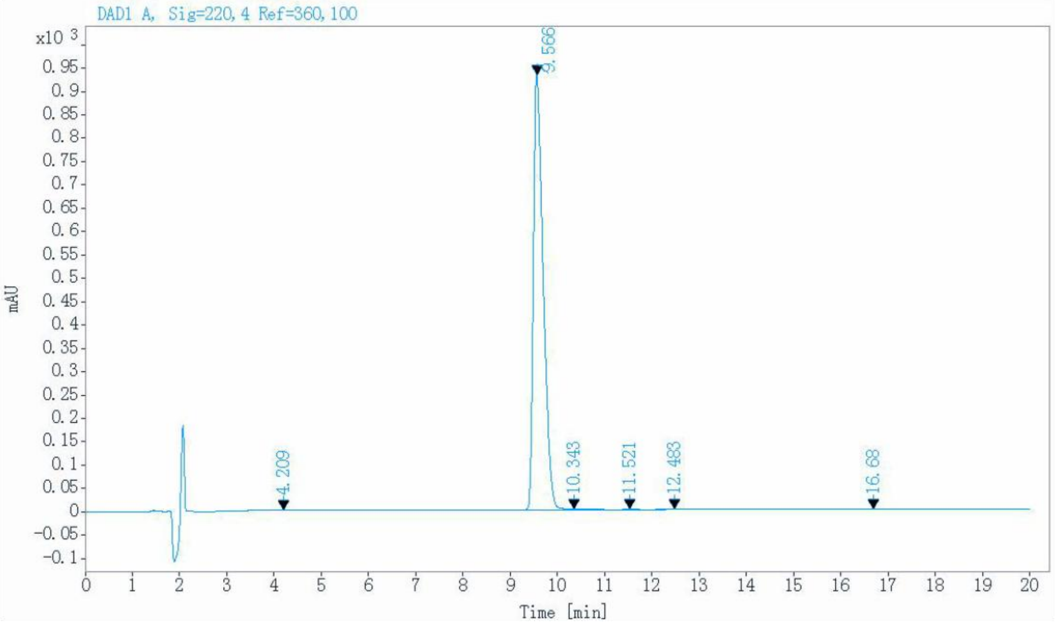

Signal: DAD1 A, Sig=220,4 Ref=360,100

RT[min]	Type	Width[min]	Area	Height	Area%	Name
4.209	BB	0.1110	6.2646	0.8803	0.0465	
9.566	VV R	0.2212	13447.1543	927.4581	99.7241	
10.343	VV E	0.2091	9.6551	0.6824	0.0716	
11.521	VB E	0.2823	6.2835	0.3239	0.0466	
12.483	BV	0.2912	6.5041	0.3069	0.0482	
16.680	FM	0.2382	8.5007	0.5949	0.0630	
		Sum	13484.3623			

The LRMS and HPLC purity data of VTP-23

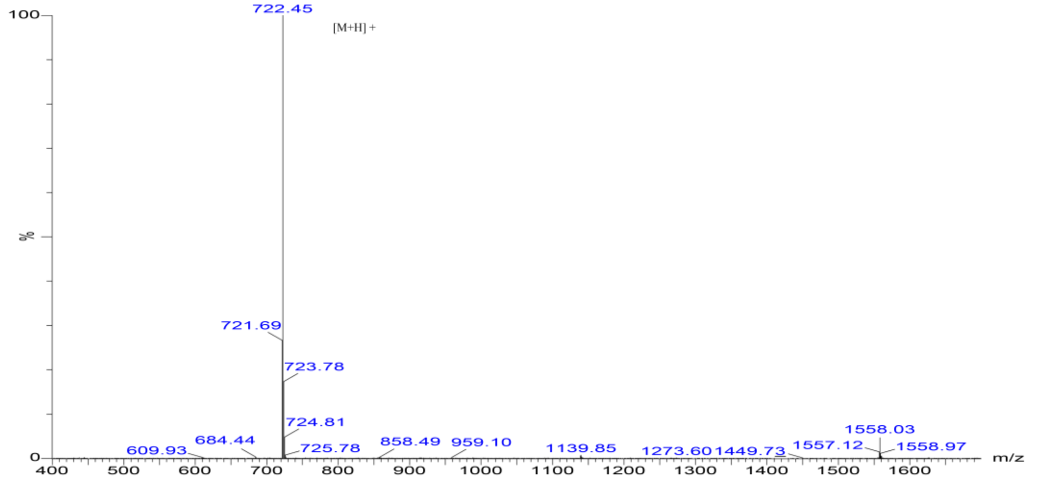

Description: Mobile phase:Buffer A:0.1%TFA in H2O
Buffer B:0.1%TFA in ACN
Gradient:24-34% B in 20min , Flow rate:1.0ml/min
Column:Unitary C18,4.6*150mm,5um 100A

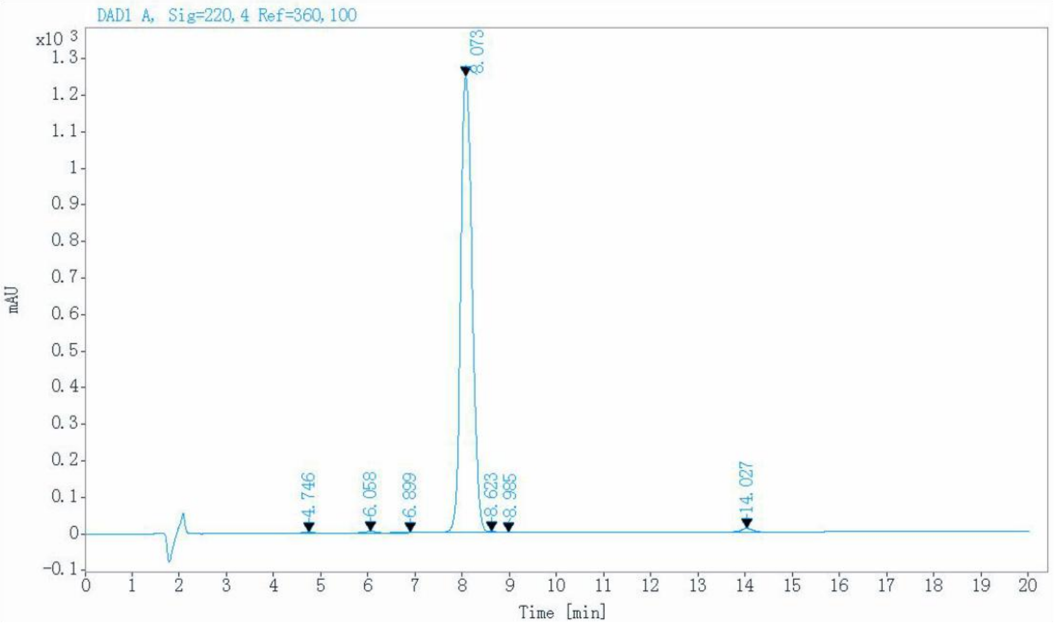

Signal: DAD1 A, Sig=220,4 Ref=360,100

RT[min]	Type	Width[min]	Area	Height	Area%	Name
4.746	BB	0.2056	38.8321	2.6719	0.1819	
6.058	BB	0.2111	68.6114	4.9085	0.3214	
6.899	MF	0.4405	62.7098	2.3729	0.2938	
8.073	FM	0.2795	20962.5664	1250.1851	98.2093	
8.623	MF	0.1675	27.0923	2.6952	0.1269	
8.985	FM	0.2927	23.8434	1.3576	0.1117	
14.027	BBA	0.2547	161.1352	9.6531	0.7549	
		Sum	21344.7905			

The LRMS and HPLC purity data of VTP-24

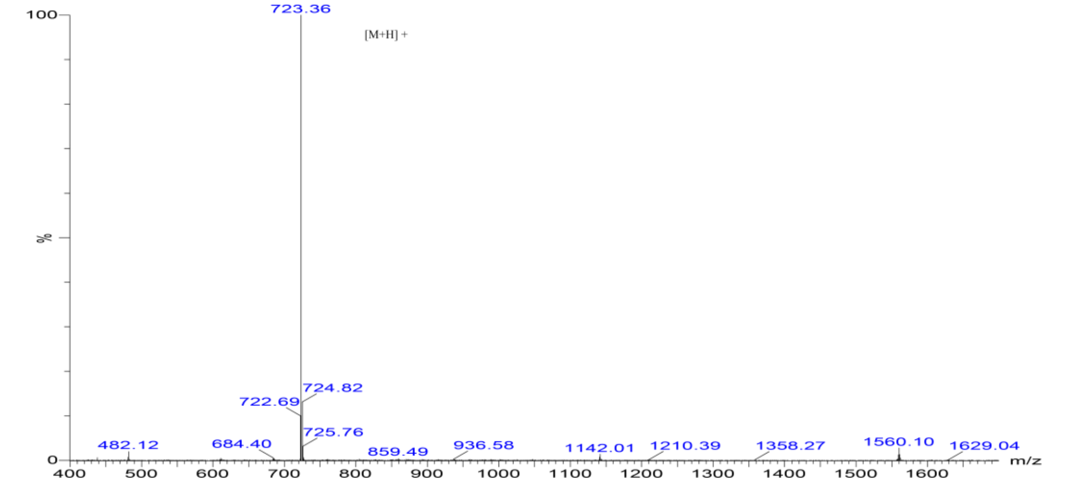

Description: Mobile phase:Buffer A:0.1%TFA in H2O
Buffer B:0.1%TFA in ACN
Gradient:21-31% B in 20min , Flow rate:1.0ml/min
Column:Unitary C18,4.6*150mm,5um 100A

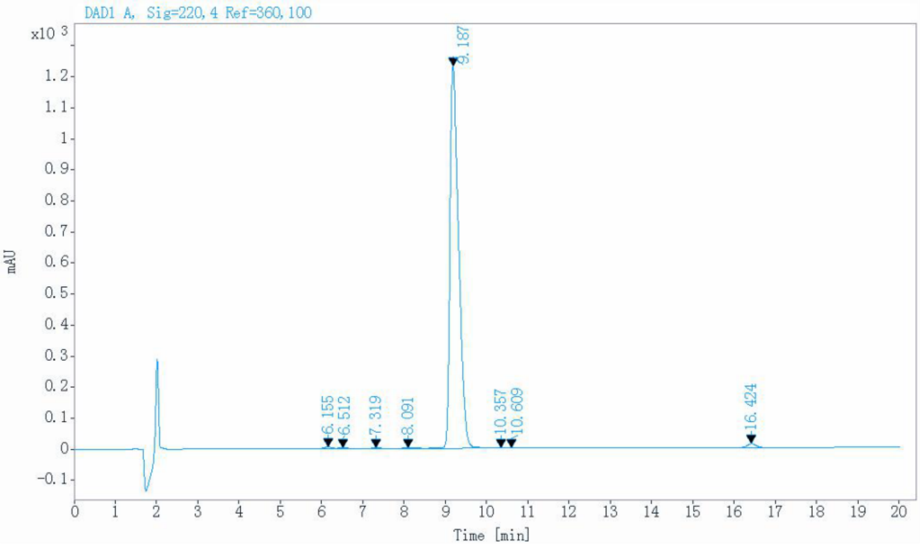

Signal: DAD1 A, Sig=220,4 Ref=360,100

RT[min]	Type	Width[min]	Area	Height	Area%	Name
6.155	VV R	0.1714	52.5234	4.8104	0.2877	
6.512	VB	0.1791	29.2502	2.5256	0.1602	
7.319	BV	0.1906	12.5215	1.0245	0.0686	
8.091	VB	0.3251	11.7454	0.4875	0.0643	
9.187	BV	0.2218	17941.3164	1232.6654	98.2843	
10.357	VV	0.2465	20.2602	1.1231	0.1110	
10.609	VB	0.2329	13.3173	0.7890	0.0730	
16.424	BB	0.2224	173.5771	12.1671	0.9509	
		Sum	18254.5116			

The LRMS and HPLC purity data of VTP-25

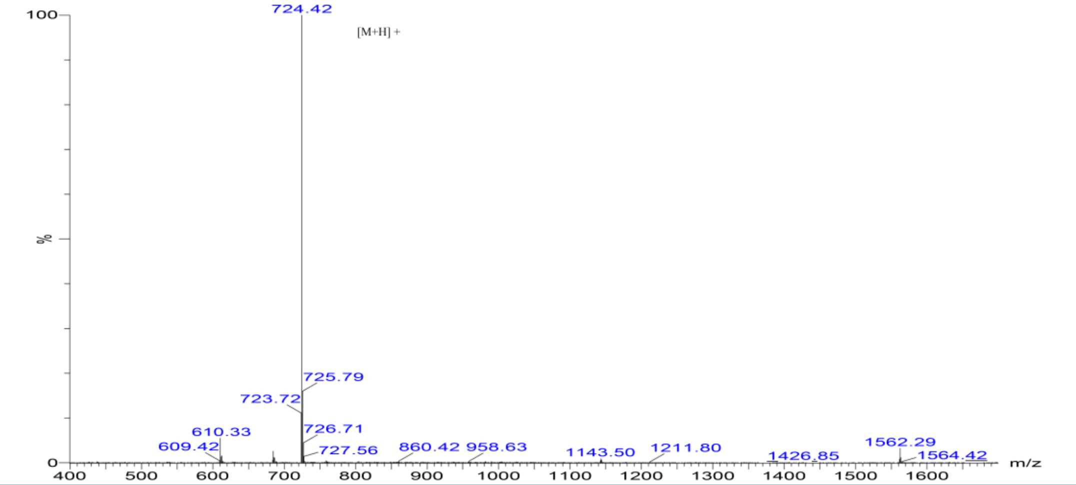

Description: Mobile phase:Buffer A:0.1%TFA in H2O
Buffer B:0.1%TFA in ACN
Gradient:17-27% B in 20min , Flow rate:1.0ml/min
Column:Unitary C18,4.6*150mm,5um 100A

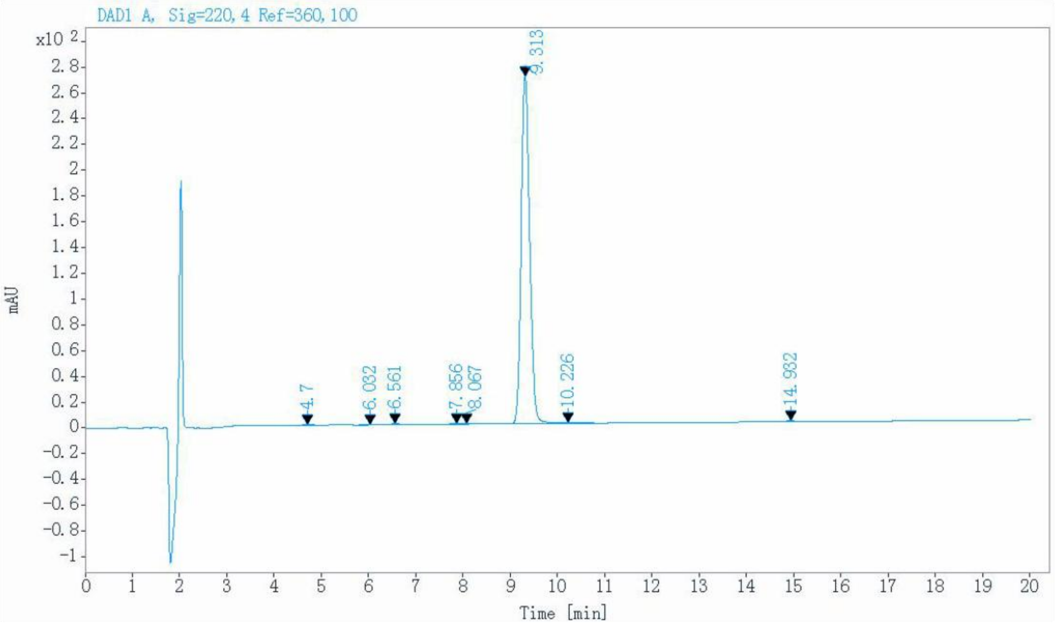

Signal: DAD1 A, Sig=220,4 Ref=360,100

RT[min]	Type	Width[min]	Area	Height	Area%	Name
4.700	BV	0.3547	11.9169	0.4363	0.3508	
6.032	BV	0.1856	6.0256	0.4965	0.1774	
6.561	VV	0.2210	12.1847	0.7853	0.3586	
7.856	BV	0.2044	4.8635	0.3370	0.1432	
8.067	VB	0.1587	2.2072	0.2032	0.0650	
9.313	BV	0.1923	3335.8264	269.7558	98.1860	
10.226	VB	0.3328	11.5009	0.4583	0.3385	
14.932	BB	0.2373	12.9318	0.8509	0.3806	
		Sum	3397.4569			

The LRMS and HPLC purity data of VTP-26

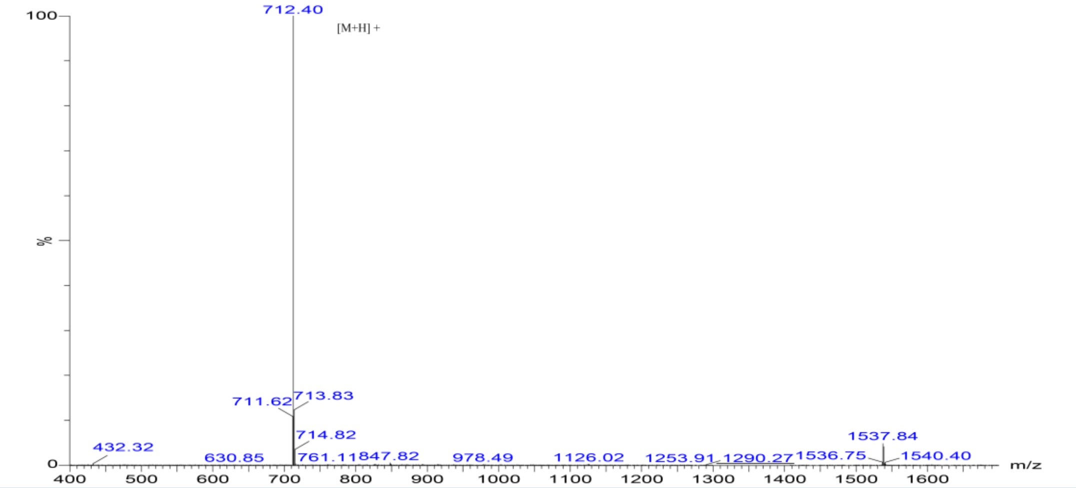

Description: Mobile phase:Buffer A:0.1%TFA in H2O

Buffer B:0.1%TFA in ACN

Gradient:20-30% B in 20min, Flow rate:1.0ml/min

Column:Unitary C18,4.6*150mm,5um 100A

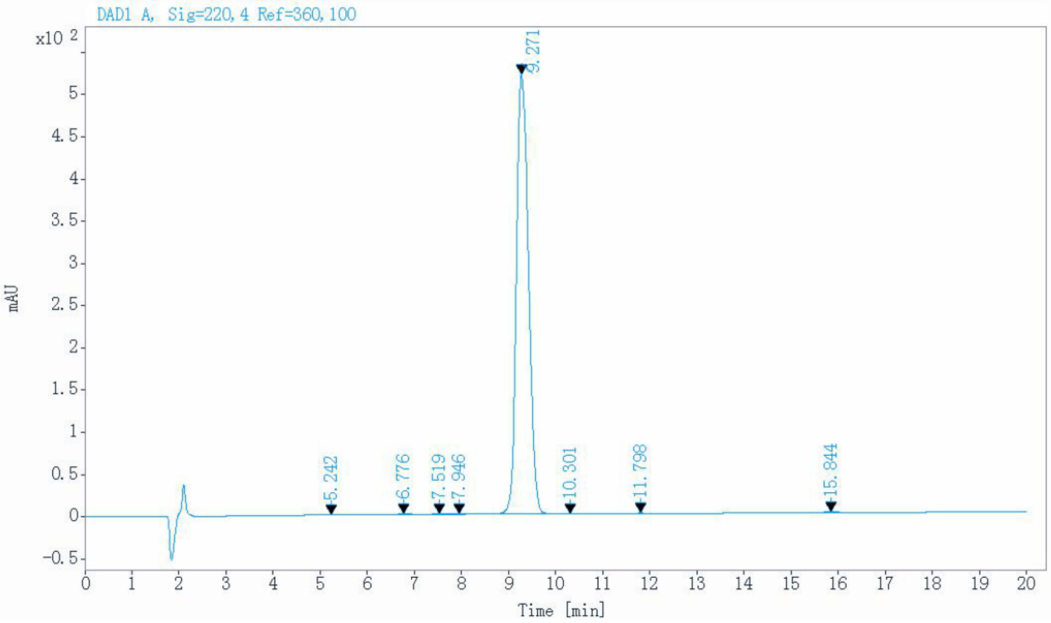

Signal: DAD1 A, Sig=220,4 Ref=360,100

RT[min]	Type	Width[min]	Area	Height	Area%	Name
5.242	BB	0.2067	6.2818	0.4396	0.0674	
6.776	BB	0.2078	22.4643	1.6399	0.2409	
7.519	BV	0.2351	16.4776	1.0732	0.1767	
7.946	VB	0.2755	9.1699	0.4122	0.0983	
9.271	BV R	0.2830	9221.8867	519.7267	98.8793	
10.301	VB E	0.4326	19.2108	0.5952	0.2060	
11.798	BBA	0.2166	5.0535	0.3498	0.0542	
15.844	BB	0.2348	25.8615	1.6502	0.2773	
		Sum	9326.4063			

The LRMS and HPLC purity data of VTP-27

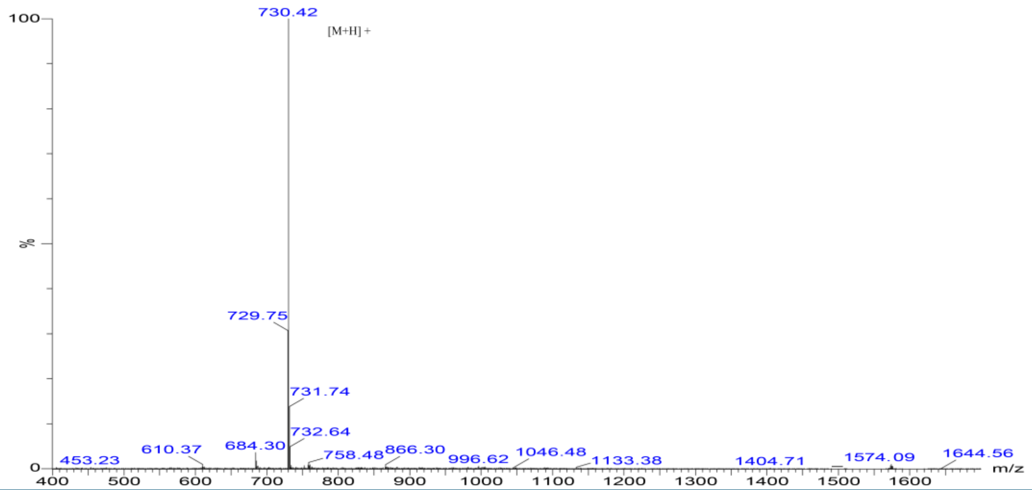

Description: Mobile phase:Buffer A:0.1%TFA in H2O
Buffer B:0.1%TFA in ACN
Gradient:22-32% B in 20min, Flow rate:1.0ml/min
Column:Unitary C18,4.6*150mm,5um 100A

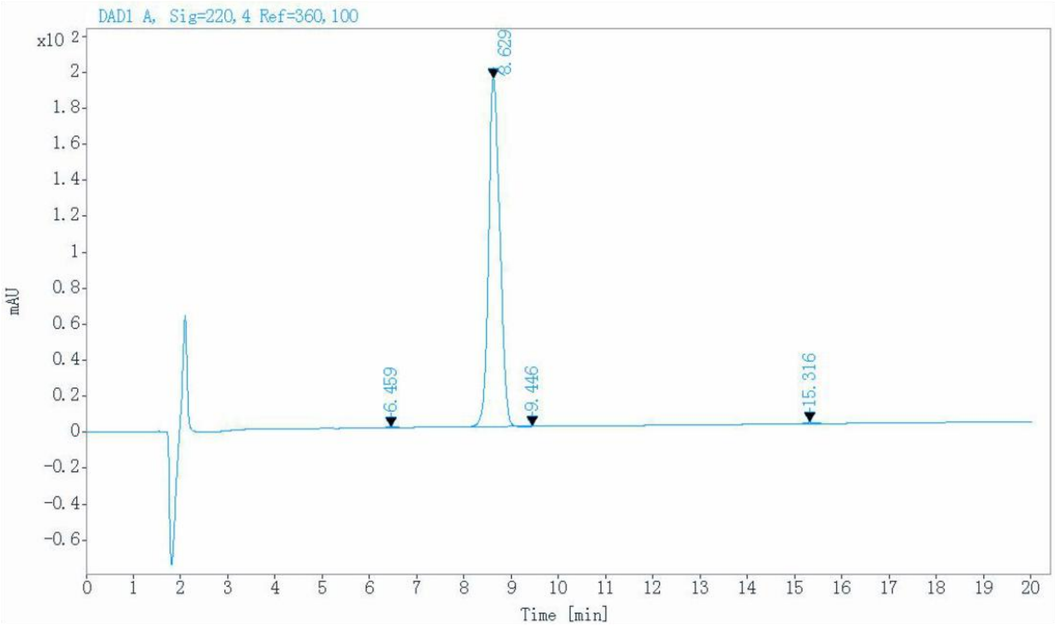

Signal: DAD1 A, Sig=220,4 Ref=360,100

RT[min]	Type	Width[min]	Area	Height	Area%	Name
6.459	BBA	0.2166	8.8630	0.6437	0.2737	
8.629	BV R	0.2568	3205.7690	193.9465	99.0043	
9.446	VB E	0.4955	9.9197	0.2785	0.3064	
15.316	BB	0.2722	13.4590	0.7844	0.4157	
		Sum	3238.0108			

The LRMS and HPLC purity data of VTP-28

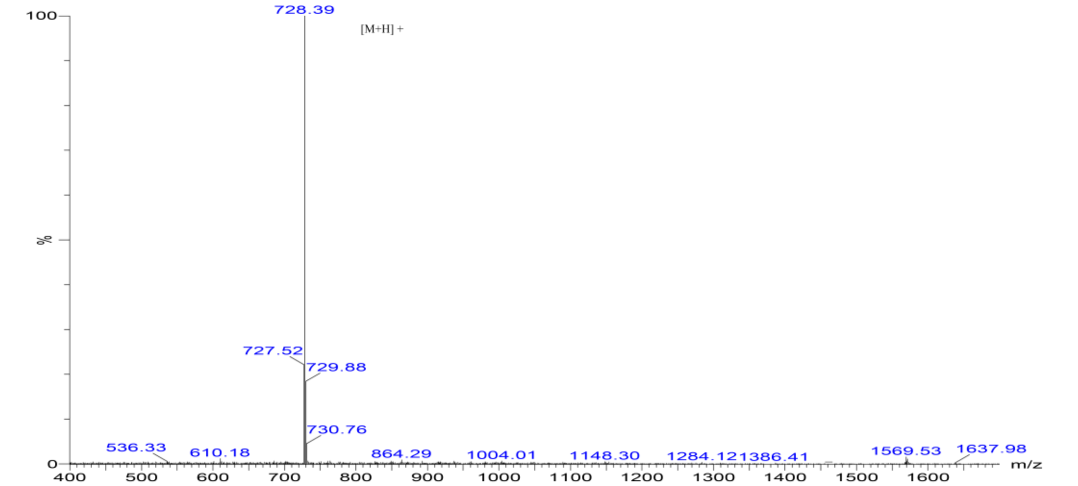

Description: Mobile phase:Buffer A:0.1%TFA in H2O
Buffer B:0.1%TFA in ACN
Gradient:23-33% B in 20min, Flow rate:1.0ml/min
Column:Unitary C18,4.6*150mm,5um 100A

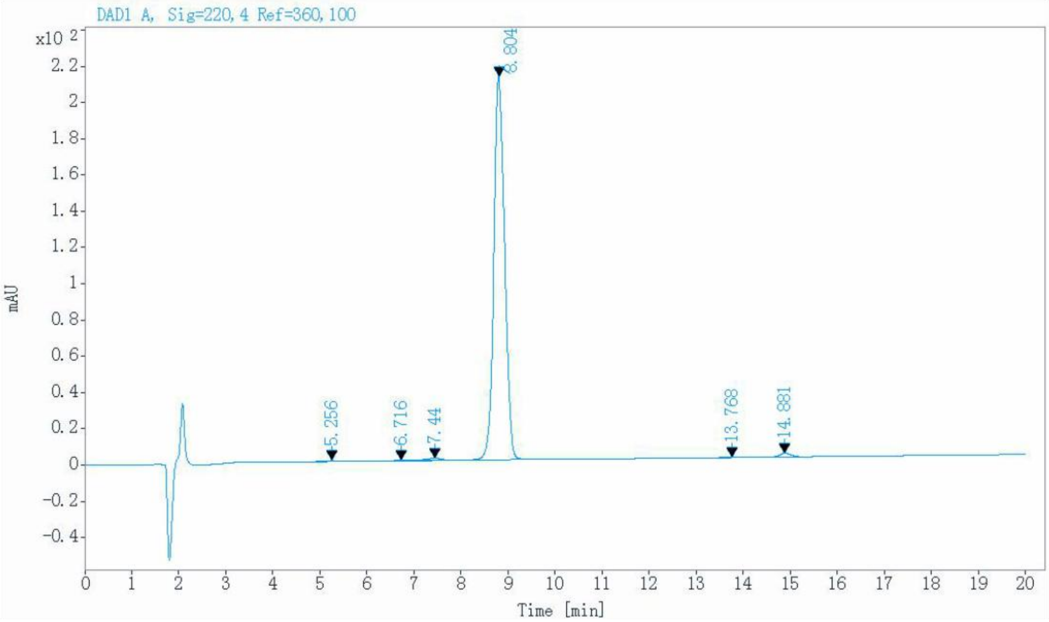

Signal: DAD1 A, Sig=220,4 Ref=360,100

RT[min]	Type	Width[min]	Area	Height	Area%	Name
5.256	MM	0.2247	7.0187	0.5207	0.2015	
6.716	BV E	0.2492	8.8798	0.5363	0.2549	
7.440	VV E	0.3811	40.8670	1.4666	1.1732	
8.804	VB R	0.2600	3380.7458	209.8469	97.0571	
13.768	MF	0.4516	11.3683	0.4196	0.3264	
14.881	FM	0.2752	34.3767	2.0821	0.9869	
		Sum	3483.2563			

The LRMS and HPLC purity data of VTP-29

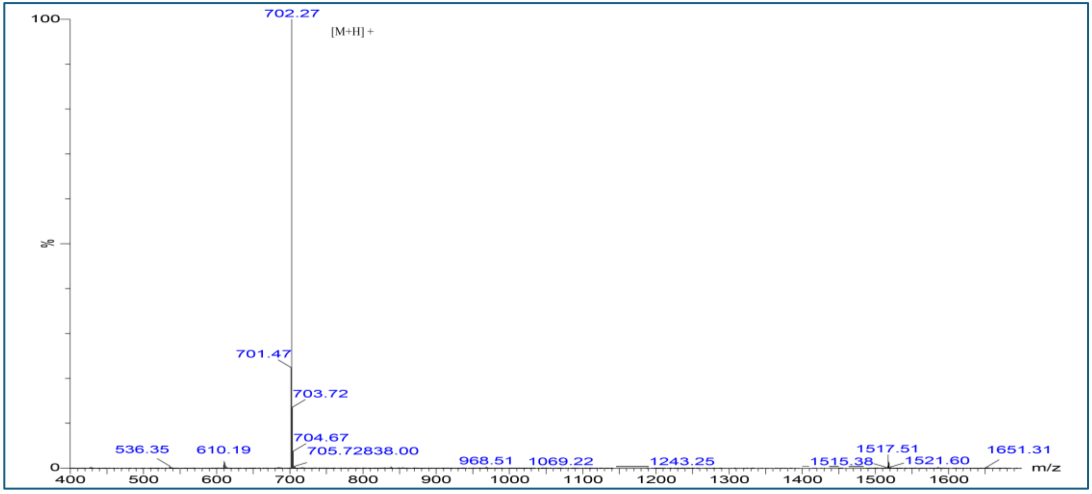

Description: Mobile phase:Buffer A:0.1%TFA in H2O
Buffer B:0.1%TFA in ACN
Gradient:20-30% B in 20min , Flow rate:1.0ml/min
Column:Unitary C18,4.6*150mm,5um 100A

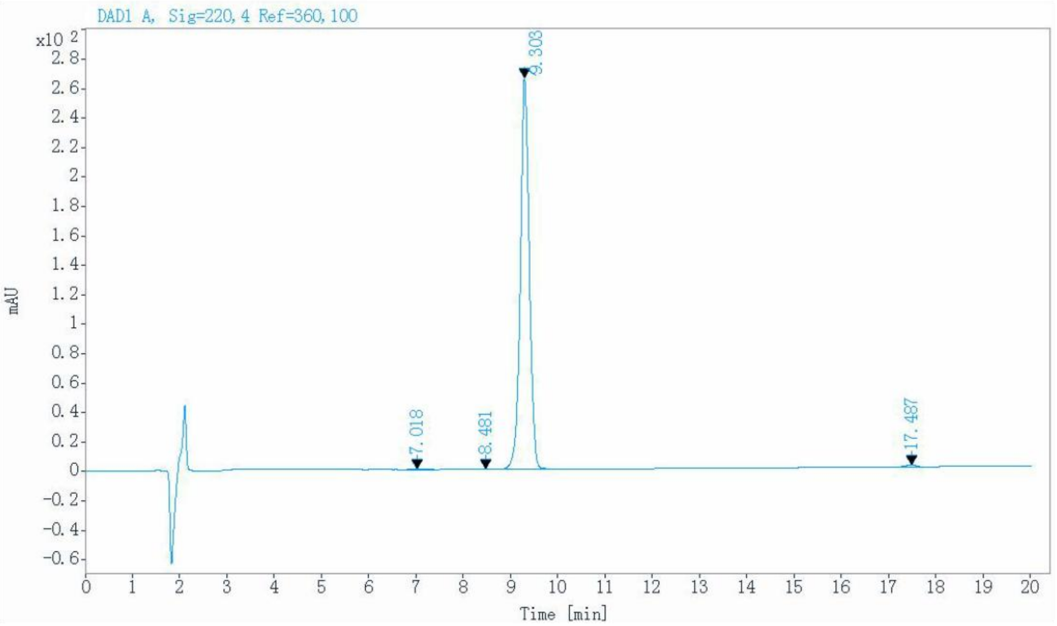

Signal: DAD1 A, Sig=220,4 Ref=360,100

RT[min]	Type	Width[min]	Area	Height	Area%	Name
7.018	BB	0.2455	5.4444	0.3499	0.1550	
8.481	BV E	0.2156	5.5619	0.4067	0.1584	
9.303	VB R	0.2086	3479.3123	265.9956	99.0697	
17.487	FM	0.2403	21.6654	1.5024	0.6169	
		Sum	3511.9839			

The LRMS and HPLC purity data of VTP-30

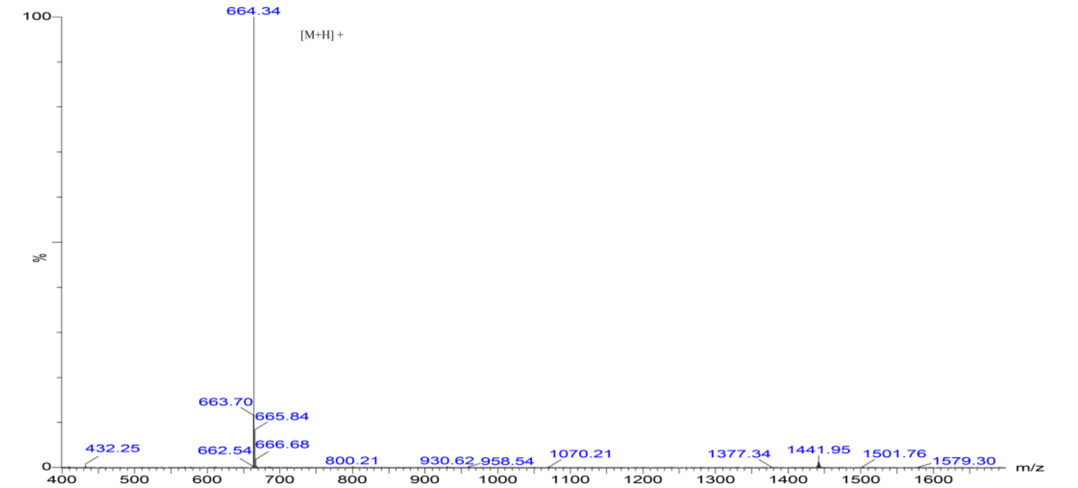

Description: Mobile phase:Buffer A:0.1%TFA in H2O
Buffer B:0.1%TFA in ACN
Gradient:12-22% B in 20min , Flow rate:1.0ml/min
Column:Unitary C18,4.6*150mm,5um 100A

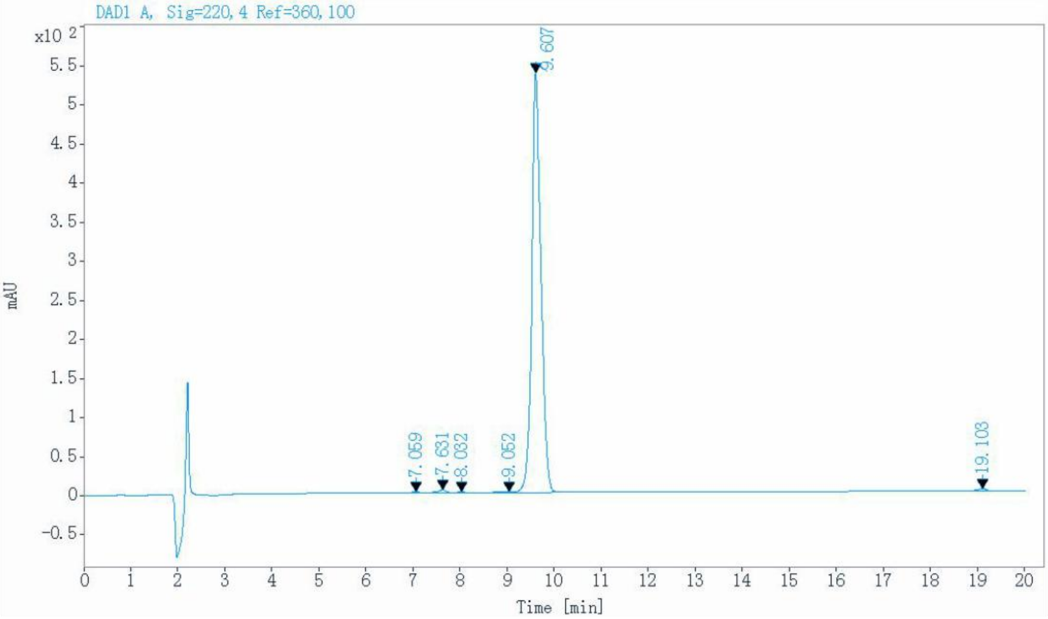

Signal: DAD1 A, Sig=220,4 Ref=360,100

RT[min]	Type	Width[min]	Area	Height	Area%	Name
7.059	BV	0.1690	18.2037	1.5733	0.2469	
7.631	VV	0.1922	47.0288	3.5577	0.6378	
8.032	MM	0.2095	9.1122	0.7248	0.1236	
9.052	MF	0.3542	15.8501	0.7457	0.2150	
9.607	FM	0.2245	7242.4731	537.7343	98.2249	
19.103	FM	0.2322	40.6896	2.9204	0.5518	
		Sum	7373.3574			

The LRMS and HPLC purity data of VTP-31

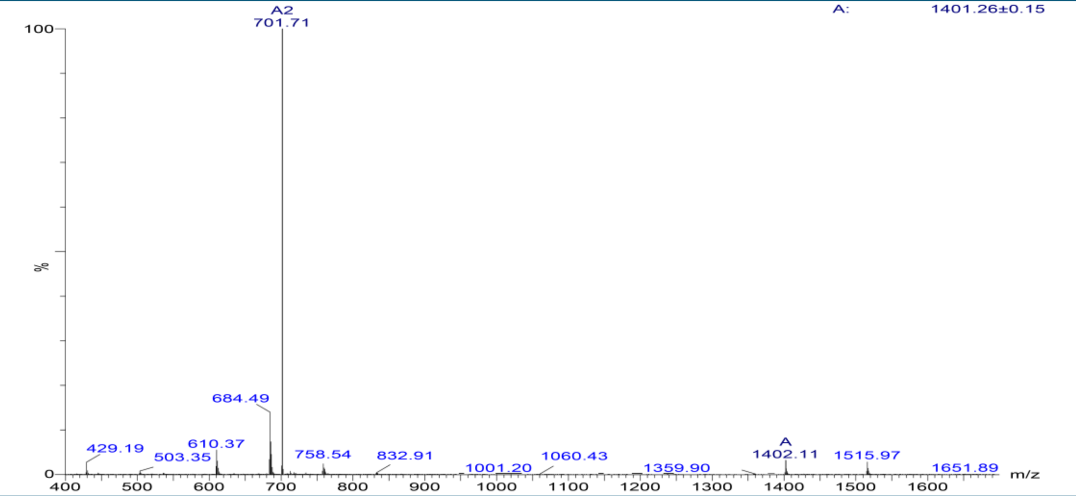

Description: Mobile phase:Buffer A:0.1%TFA in H2O
Buffer B:0.1%TFA in ACN
Gradient:25-35% B in20min, Flow rate:1ml/min
Column:Unitary C18,4.6*150mm,5um 100A

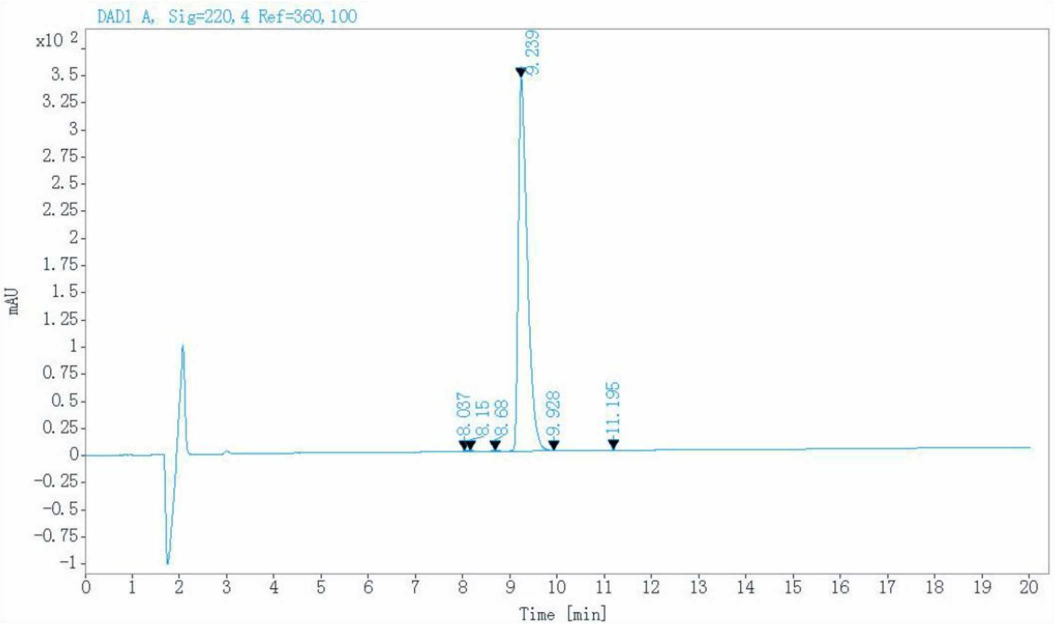

Signal: DAD1 A, Sig=220,4 Ref=360,100

RT[min]	Type	Width[min]	Area	Height	Area%	Name
8.037	BV F	0.1343	5.4273	0.6069	0.1183	
8.150	VB	0.1241	5.7158	0.7086	0.1246	
8.680	MF	0.2158	8.0237	0.6198	0.1749	
9.239	FM	0.2205	4559.9023	344.6234	99.3996	
9.928	FM	0.1827	5.2419	0.4783	0.1143	
11.195	MM	0.1954	3.1359	0.2675	0.0684	
		Sum	4587.4470			

The LRMS and HPLC purity data of VTP-32

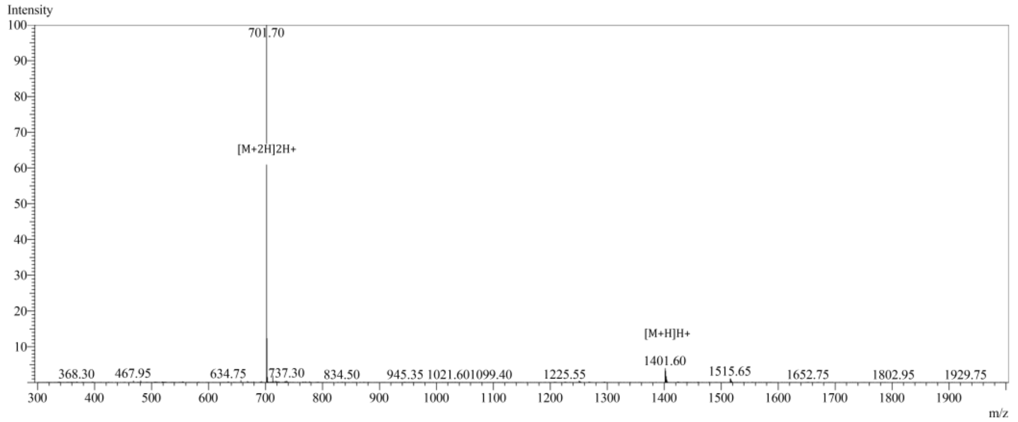

Pump A :0.1%Trifluoroacetic in 100% water
Pump B :0.1%Trifluoroacetic in 100% acetonitrile
Total Flow :1ml/min
Wavelength :220nm
Analytical column type :SHIMADZU Inertsil ODS-SP(4.6*250mm*5um)
Dissolution method :15%ACN+85%H2O
Inj. Volume :15uL

Time	Module	Action	Value
0.01	Pumps	B.Conc	20
20.00	Pumps	B.Conc	60
23.00	Pumps	B.Conc	100
38.00	Pumps	B.Conc	100
40.00	Pumps	B.Conc	20
50.00	Controller	Stop	

Chromatogram

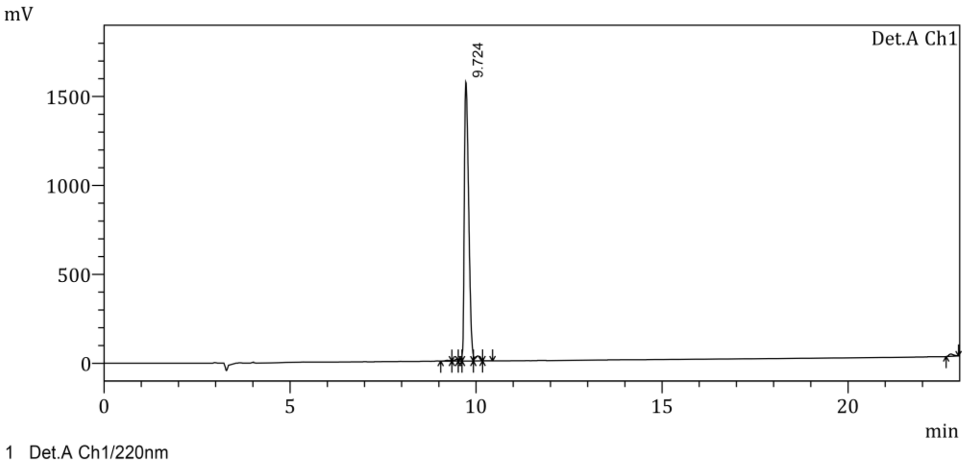

PeakTable

Detector A Ch1 220nm

Peak#	Ret. Time	Area	Height	Area %	Height %
1	9.217	34498	4017	0.260	0.245
2	9.472	59636	9267	0.450	0.565
3	9.617	61730	15978	0.466	0.975
4	9.724	12714254	1567623	95.963	95.652
5	10.043	248241	27665	1.874	1.688
6	10.183	11887	1414	0.090	0.086
7	22.760	118822	12925	0.897	0.789
Total		13249067	1638889	100.000	100.000

The LRMS and HPLC purity data of VTP-33

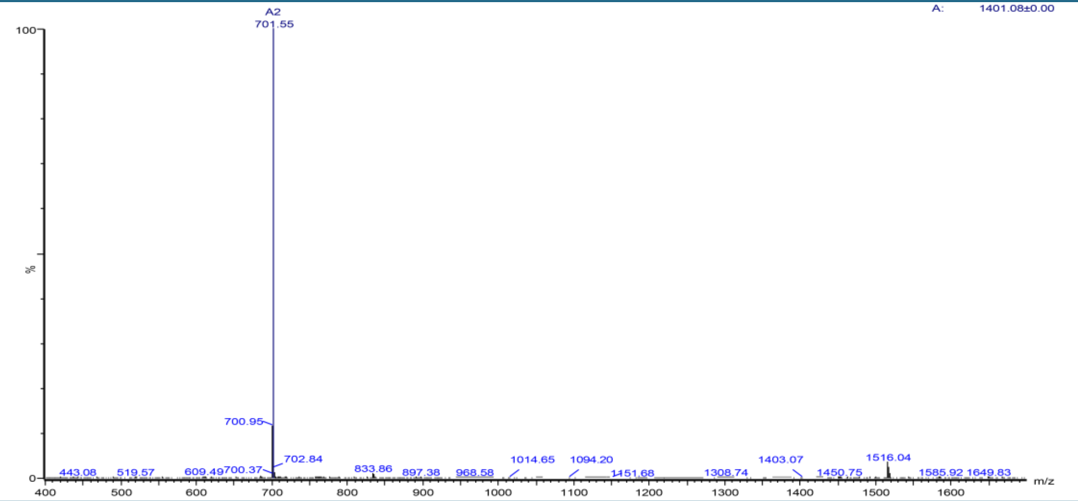

Description: Mobile phase:Buffer A:0.1%TFA in H2O
Buffer B:0.1%TFA in ACN
Gradient:24-34% B in20min , Flow rate:1ml/min
Column:Unitary C18, 4.6*150mm, 5um 100A

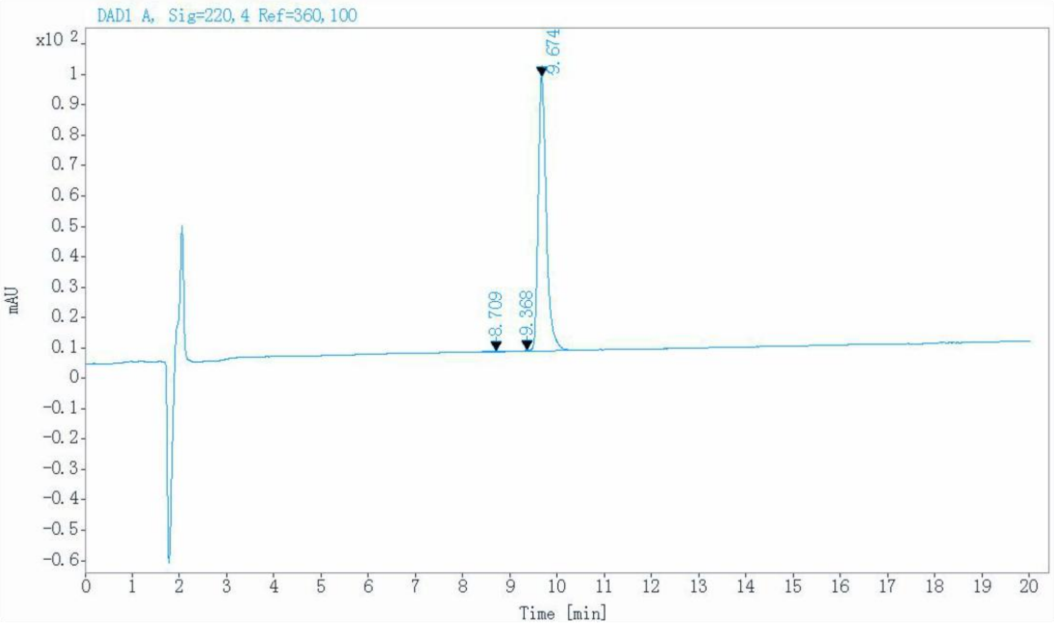

Signal: DAD1 A, Sig=220, 4 Ref=360, 100

RT[min]	Type	Width[min]	Area	Height	Area%	Name
8.709	VV E	0.1242	1.5879	0.2009	0.1470	
9.368	VV E	0.1167	2.2768	0.2748	0.2108	
9.674	VBAR	0.1831	1076.1362	90.2377	99.6422	
		Sum	1080.0010			

The LRMS and HPLC purity data of VTP-34

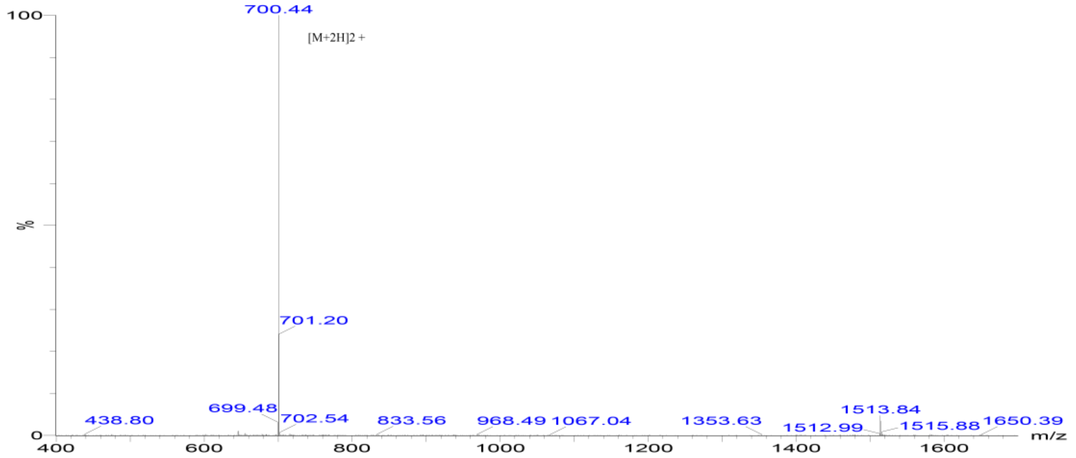

Description: Mobile phase:Buffer A:0.1%TFA in H2O
Buffer B:0.1%TFA in ACN
Gradient:29-39% B in 20min , Flow rate:1.0ml/min
Column:Unitary C18, 4.6*150mm, 5um 100A

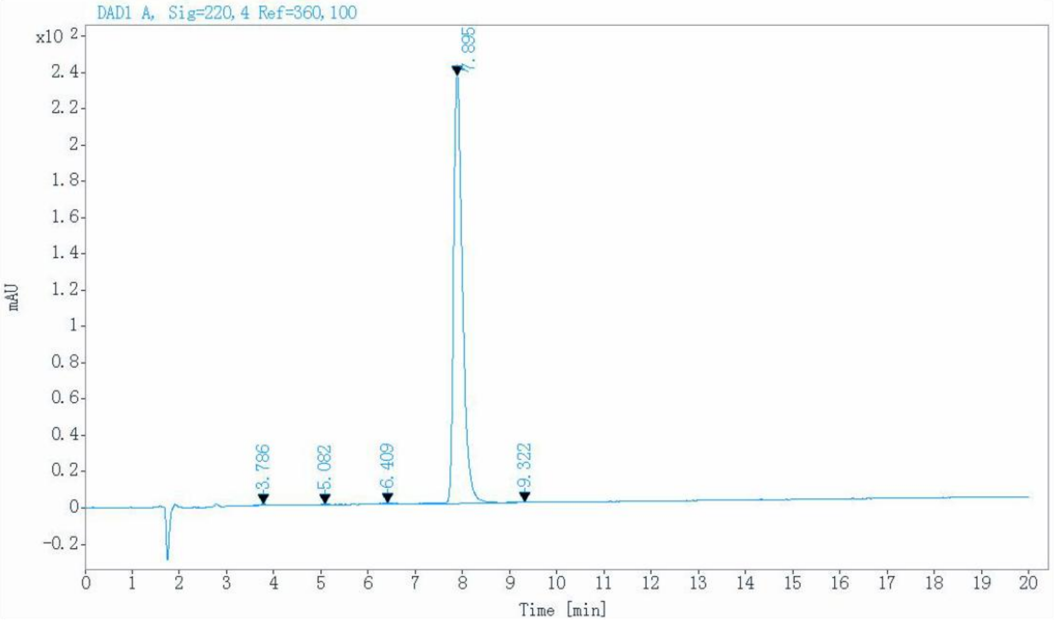

Signal: DAD1 A, Sig=220, 4 Ref=360, 100

RT[min]	Type	Width[min]	Area	Height	Area%	Name
3.786	BB	0.1869	9.8975	0.8312	0.3175	
5.082	BB	0.3298	10.5557	0.4250	0.3387	
6.409	BB	0.3213	13.6182	0.5989	0.4369	
7.895	BB	0.1990	3076.9832	237.6725	98.7206	
9.322	BV F	0.1903	5.8059	0.4506	0.1863	
		Sum	3116.8605			

The LRMS and HPLC purity data of VTP-35

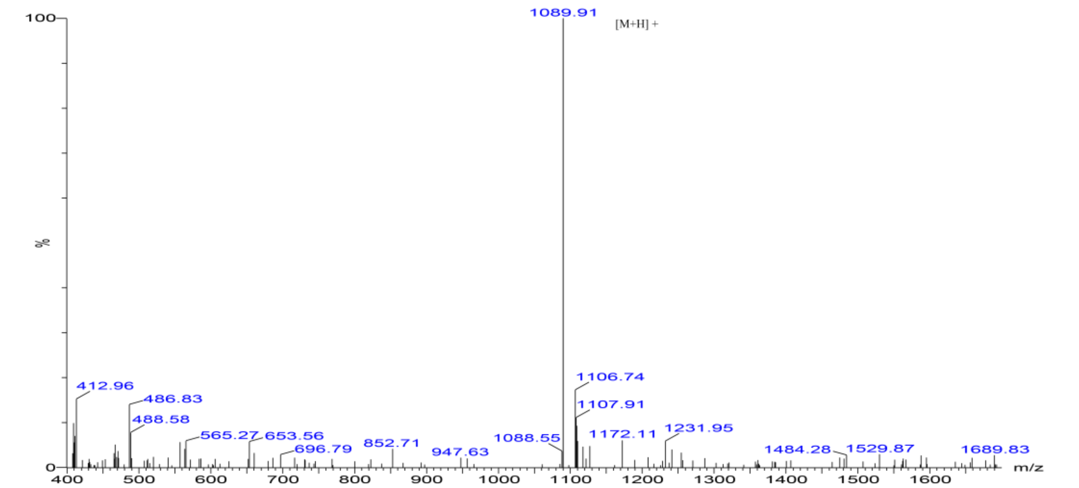

Description: Mobile phase:Buffer A:0.1%TFA in H2O
Buffer B:0.1%TFA in ACN
Gradient:30-40% B in20min, Flow rate:1ml/min
Column:Unitary C18, 4.6*150mm, 5um 100A

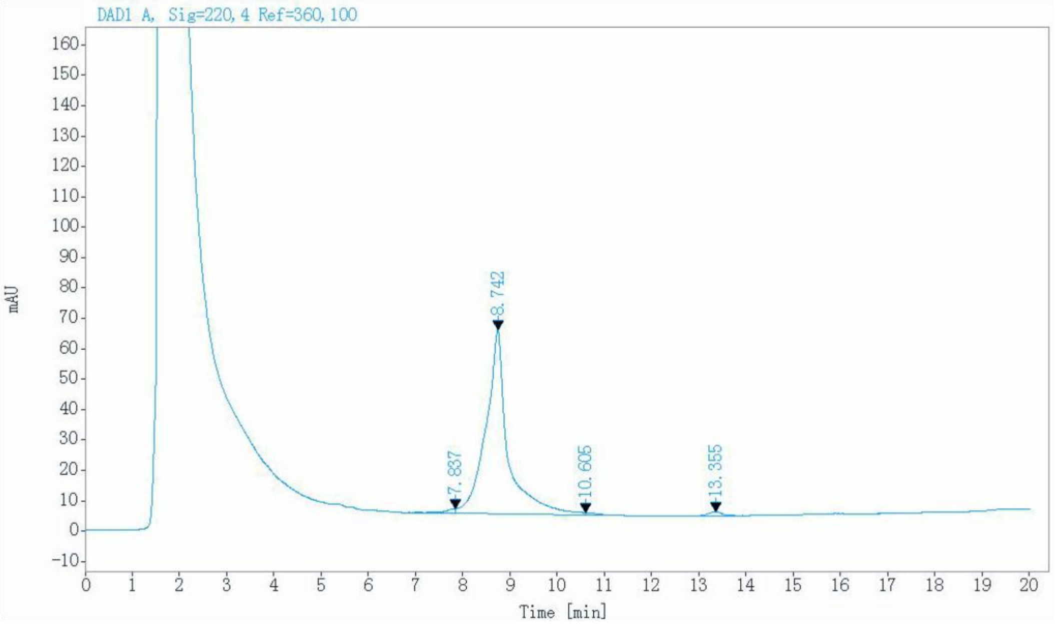

Signal: DAD1 A, Sig=220,4 Ref=360,100

RT[min]	Type	Width[min]	Area	Height	Area%	Name
7.837	MF	0.2503	21.7372	1.4475	1.0776	
8.742	FM	0.5392	1956.1571	60.4679	96.9786	
10.605	FM	0.2652	10.3249	0.6488	0.5119	
13.355	BB	0.3280	28.8835	1.3588	1.4319	
		Sum	2017.1027			

The LRMS and HPLC purity data of VTP-36

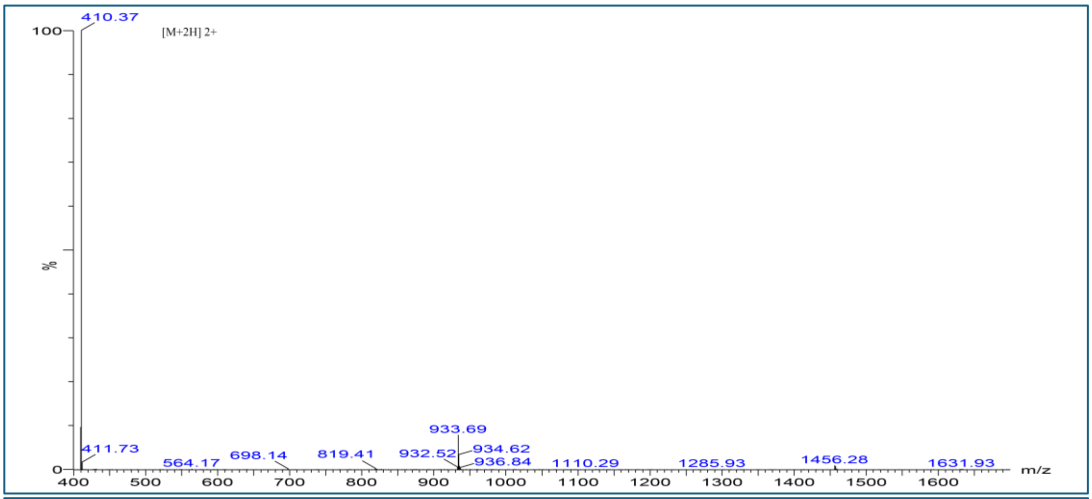

Description: Mobile phase:Buffer A:0.1%TFA in H2O
Buffer B:0.1%TFA in ACN
Gradient:26-36% B in20min , Flow rate:1ml/min
Column:Unitary C18,4.6*150mm,5um 100A

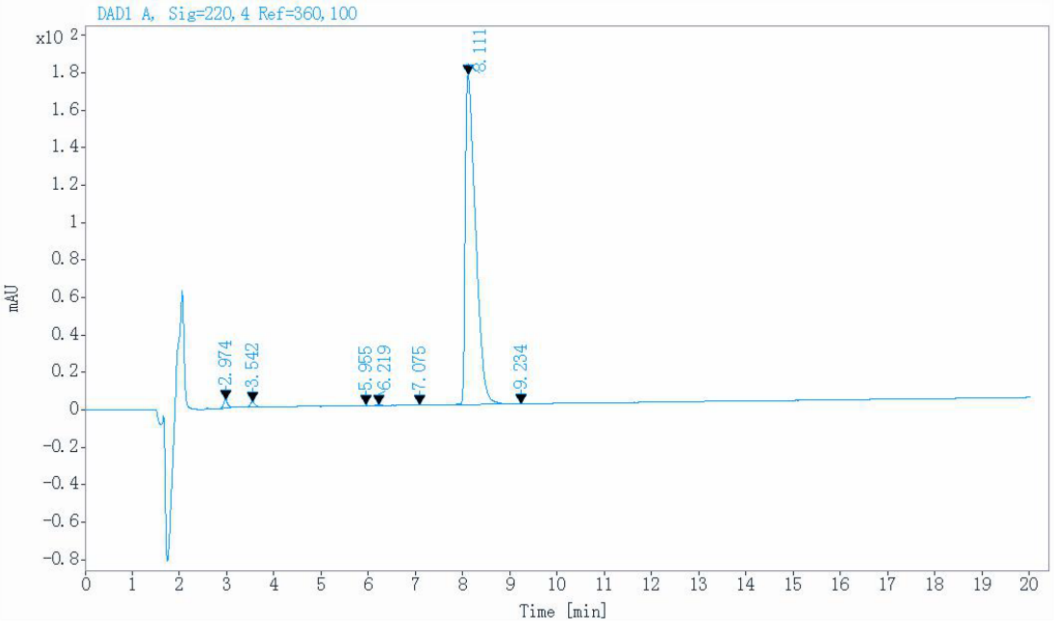

Signal: DAD1 A, Sig=220,4 Ref=360,100

RT[min]	Type	Width[min]	Area	Height	Area%	Name
2.974	BBA	0.0980	29.2071	4.6045	1.0768	
3.542	BB	0.1160	22.4019	2.9709	0.8259	
5.955	VV	0.1956	3.3991	0.2160	0.1253	
6.219	VB	0.1735	2.5571	0.2304	0.0943	
7.075	BV	0.1711	3.0331	0.2785	0.1118	
8.111	MF	0.2504	2649.2012	176.3435	97.6682	
9.234	FM	0.3442	2.6500	0.1283	0.0977	
		Sum	2712.4494			

The LRMS and HPLC purity data of VTP-37

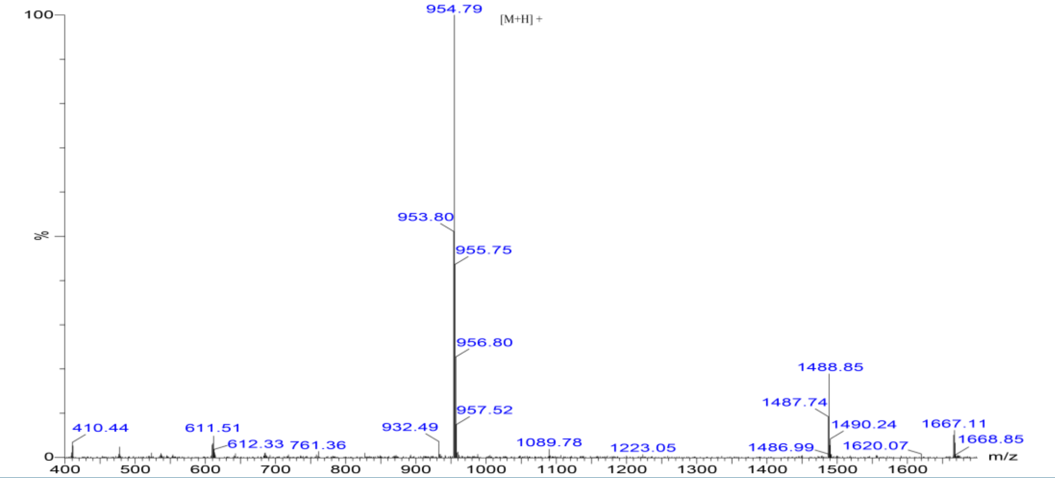

Description: Mobile phase:Buffer A:0.1%TFA in H20
Buffer B:0.1%TFA in ACN
Gradient:27-37% B in20min , Flow rate:1ml/min
Column:Unitary C18,4.6*150mm,5um 100A

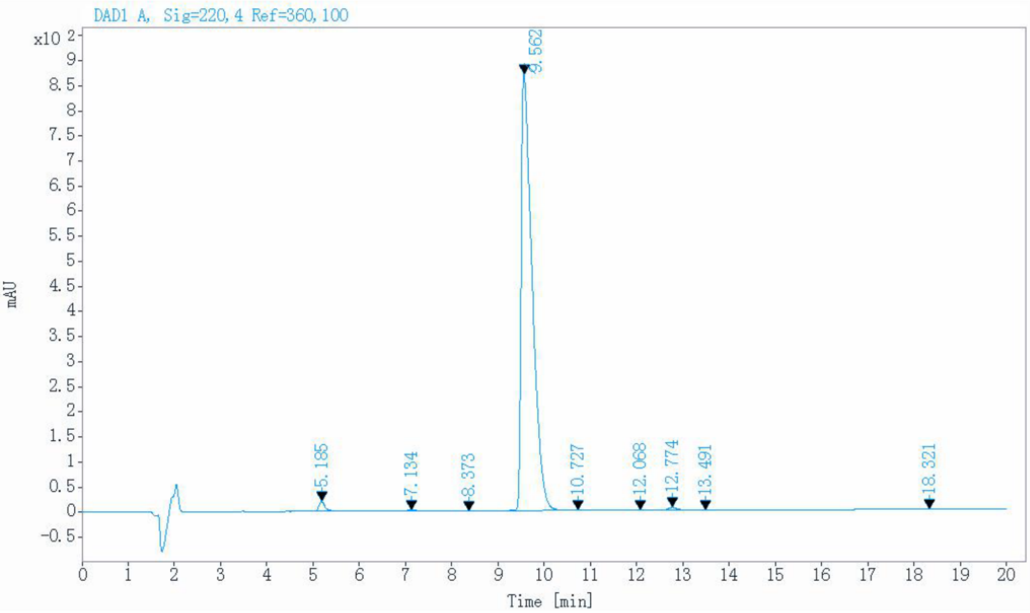

Signal: DAD1 A, Sig=220,4 Ref=360,100

RT[min]	Type	Width[min]	Area	Height	Area%	Name
5.185	MM	0.1532	179.1643	19.4948	1.2170	
7.134	BB	0.1594	29.2207	2.8551	0.1985	
8.373	BV R	0.2153	7.0695	0.4139	0.0480	
9.562	BV R	0.2536	14393.1826	867.2505	97.7691	
10.727	VV E	0.1383	2.2918	0.2424	0.0156	
12.068	VB R	0.2462	9.4676	0.5356	0.0643	
12.774	BV R	0.2110	74.4834	5.3296	0.5059	
13.491	VV E	0.1717	4.3372	0.3336	0.0295	
18.321	BV R	0.2558	22.3862	1.2567	0.1521	
		Sum	14721.6034			

The LRMS and HPLC purity data of VTP-38

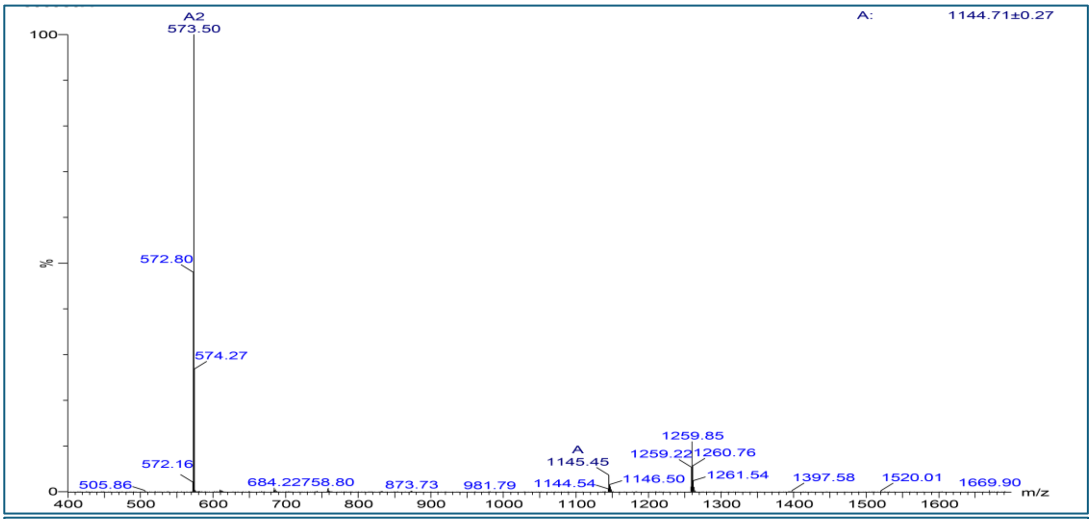

Description: Mobile phase:Buffer A:0.1%TFA in H2O
Buffer B:0.1%TFA in ACN
Gradient:25-45% B in20min , Flow rate:1ml/min
Column:Unitary C18,4.6*150mm,5um 100A

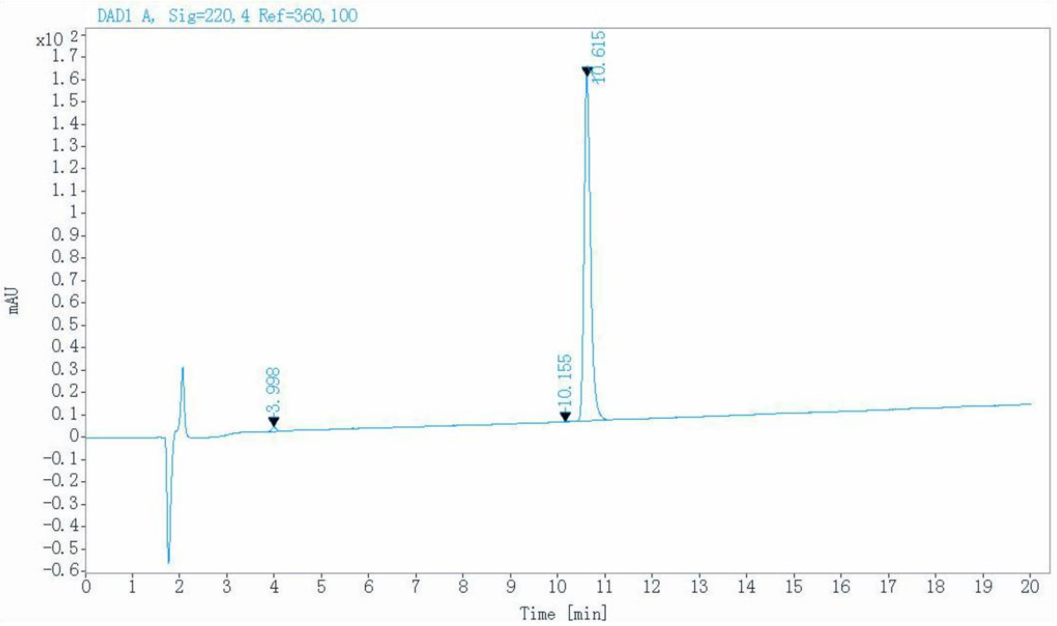

Signal: DAD1 A, Sig=220,4 Ref=360,100

RT[min]	Type	Width[min]	Area	Height	Area%	Name
3.998	BB	0.1150	12.7654	1.7946	0.8581	
10.155	MF	0.1928	0.9360	0.0809	0.0629	
10.615	FM	0.1579	1473.8660	155.6027	99.0789	
		Sum	1487.5673			

The LRMS and HPLC purity data of VTP-39

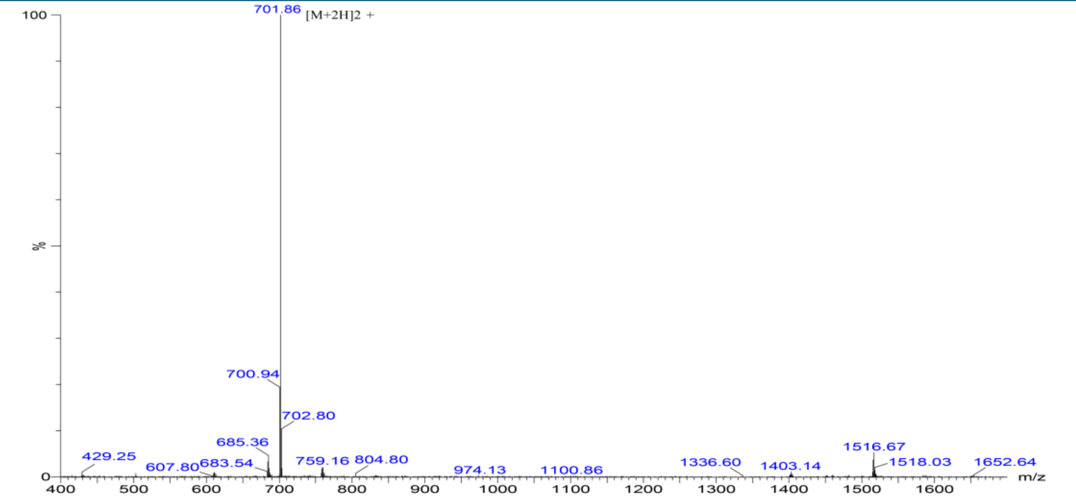

Description: Mobile phase:Buffer A:0.1%TFA in H2O
Buffer B:0.1%TFA in ACN
Gradient:20-40% B in20min , Flow rate:1ml/min
Column:Unitary C18, 4.6*150mm, 5um 100A

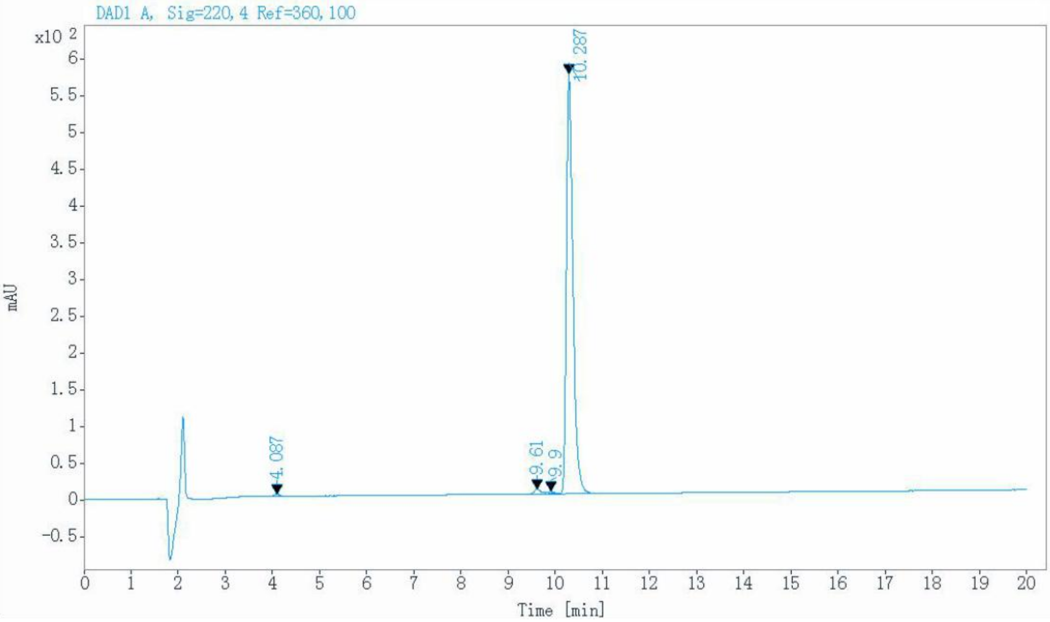

Signal: DAD1 A, Sig=220, 4 Ref=360, 100

RT[min]	Type	Width[min]	Area	Height	Area%	Name
4.087	BB	0.1080	22.3678	3.1809	0.3980	
9.610	BV E	0.1378	63.9200	6.9168	1.1374	
9.900	VV E	0.1550	32.6560	3.0000	0.5811	
10.287	VB R	0.1462	5500.7788	572.1063	97.8835	
		Sum	5619.7226			

The LRMS and HPLC purity data of VTP-40

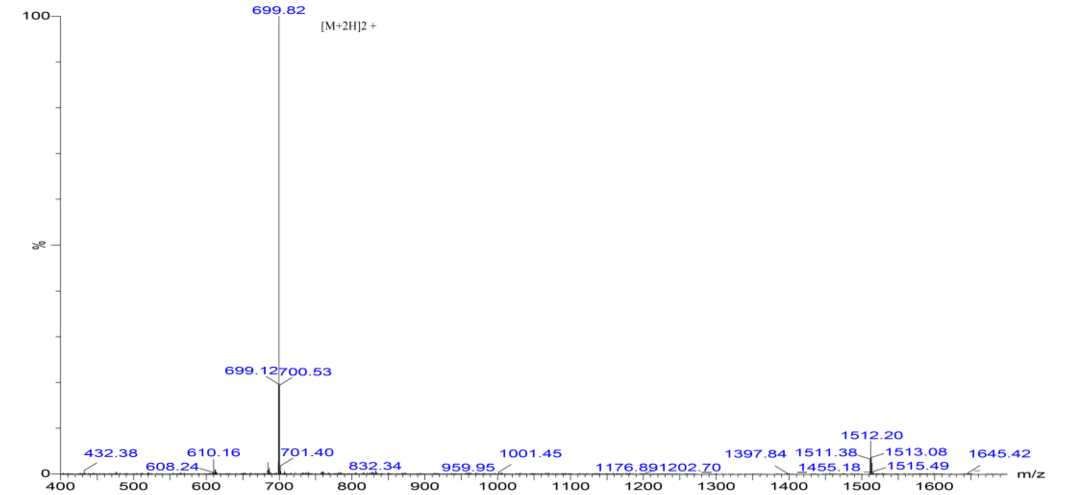

Description: Mobile phase:Buffer A:0.1%TFA in H2O
Buffer B:0.1%TFA in ACN
Gradient:24-34% B in20min , Flow rate:1ml/min
Column:Unitary C18,4.6*150mm,5um 100A

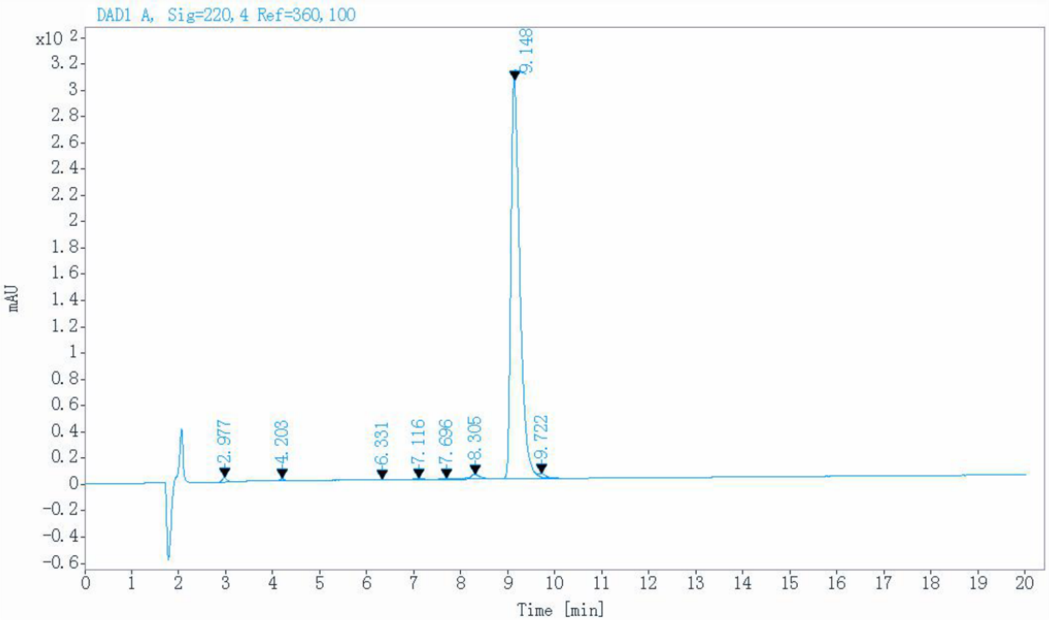

Signal: DAD1 A, Sig=220,4 Ref=360,100

RT[min]	Type	Width[min]	Area	Height	Area%	Name
2.977	BBA	0.1178	21.7196	3.2587	0.5196	
4.203	VV R	0.1441	12.1685	1.3638	0.2911	
6.331	BB	0.1705	3.4526	0.3288	0.0826	
7.116	BB	0.2546	7.1782	0.4393	0.1717	
7.696	BV E	0.2061	5.9049	0.4147	0.1413	
8.305	VB R	0.2387	53.0863	3.3896	1.2699	
9.148	BV R	0.2012	4043.7104	307.9478	96.7310	
9.722	VV E	0.1815	33.1465	2.5866	0.7929	
		Sum	4180.3671			

The LRMS and HPLC purity data of VTP-41

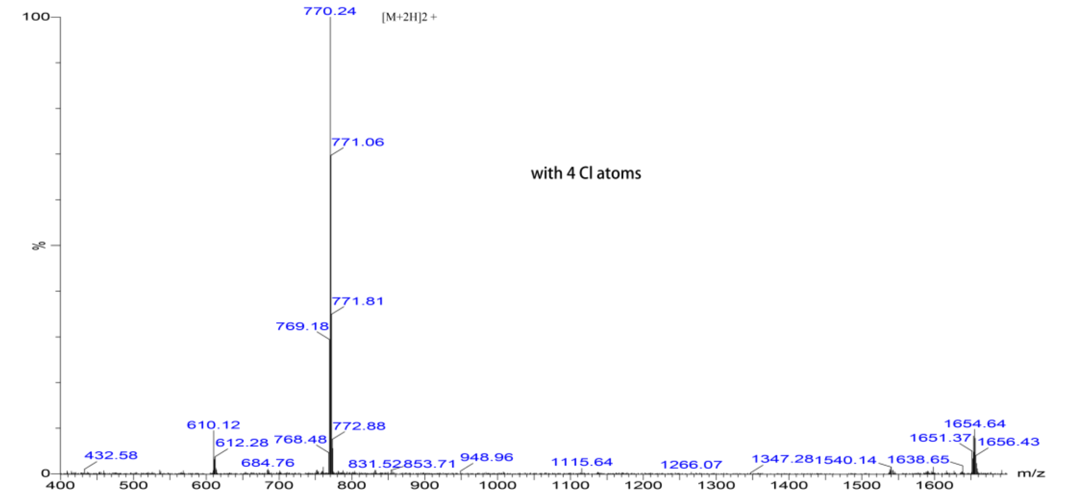

Description:
Mobile phase:Buffer A:0.1%TFA in H2O
Buffer B:0.1%TFA in ACN
Gradient:29-49% B in20min , Flow rate:1ml/min
Column:Unitary C18,4.6*150mm,5um 100A

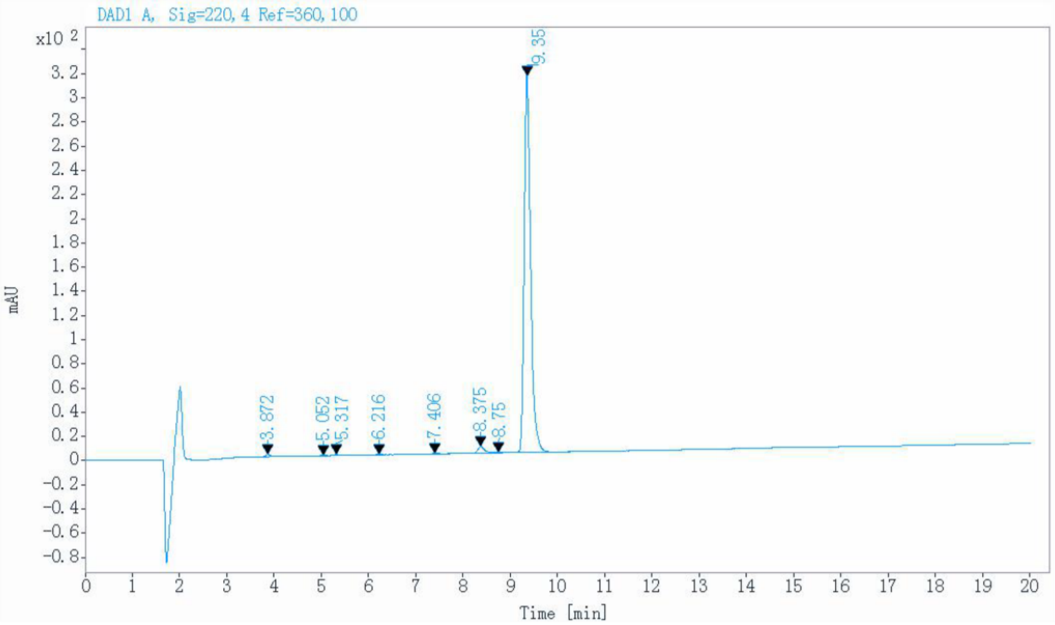

Signal: DAD1 A, Sig=220,4 Ref=360,100

RT[min]	Type	Width[min]	Area	Height	Area%	Name
3.872	MM	0.1155	13.0722	1.8868	0.4330	
5.052	MM	0.1339	2.4194	0.3011	0.0801	
5.317	MM	0.1266	6.3867	0.8409	0.2115	
6.216	MM	0.1258	4.0478	0.5361	0.1341	
7.406	MM	0.1854	4.1258	0.3710	0.1366	
8.375	MF	0.1623	54.2664	5.5714	1.7973	
8.750	FM	0.1805	4.8194	0.4449	0.1596	
9.350	VV R	0.1416	2930.1597	311.9366	97.0477	
		Sum	3019.2973			

The LRMS and HPLC purity data of VTP-42

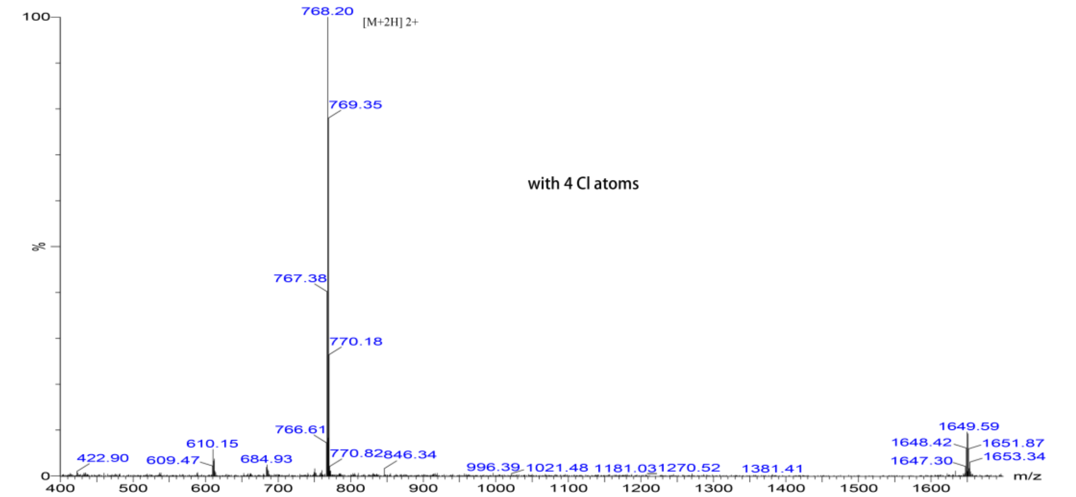

Description: Mobile phase:Buffer A:0.1%TFA in H2O

Buffer B:0.1%TFA in ACN

Gradient:30-40% B in 20min , Flow rate:1.0ml/min

Column:Unitary C18,4.6*150mm, 5um 100A

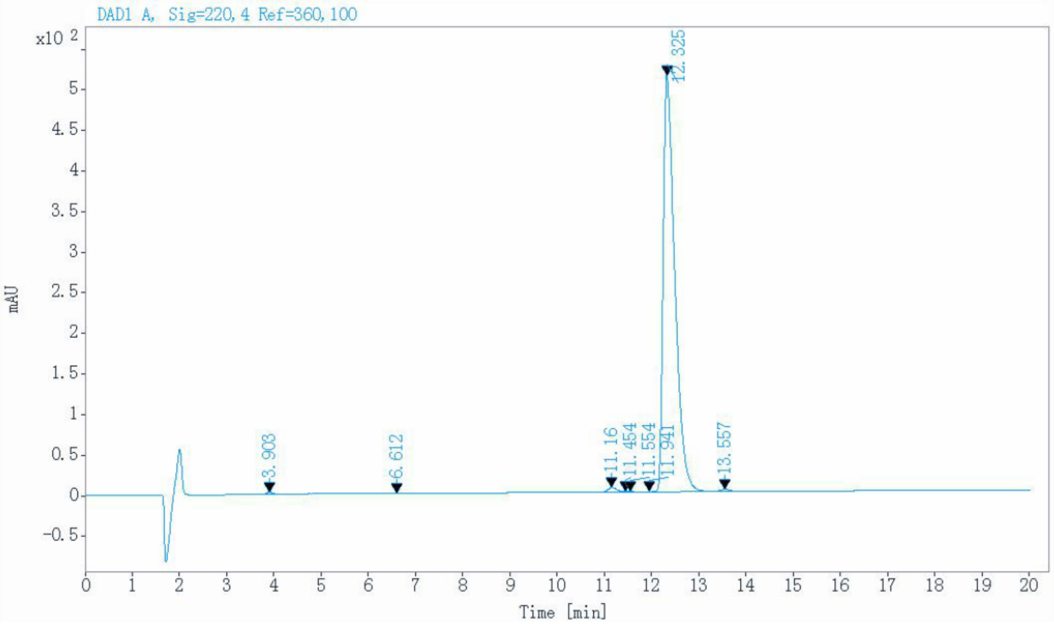

Signal: DAD1 A, Sig=220,4 Ref=360,100

RT[min]	Type	Width[min]	Area	Height	Area%	Name
3.903	BV R	0.1222	19.8935	2.4638	0.2237	
6.612	BV	0.1315	4.4832	0.5054	0.0504	
11.160	BV B	0.2050	76.3810	5.6755	0.8589	
11.454	VV	0.0982	7.2019	1.1331	0.0810	
11.554	VV B	0.0874	4.5122	0.7377	0.0507	
11.941	VV F	0.0959	1.7155	0.2385	0.0193	
12.325	VV B	0.2587	8734.1934	512.9434	98.2175	
13.557	VV R	0.2539	44.3227	2.6123	0.4984	
		Sum	8892.7033			

The LRMS and HPLC purity data of VTP-43

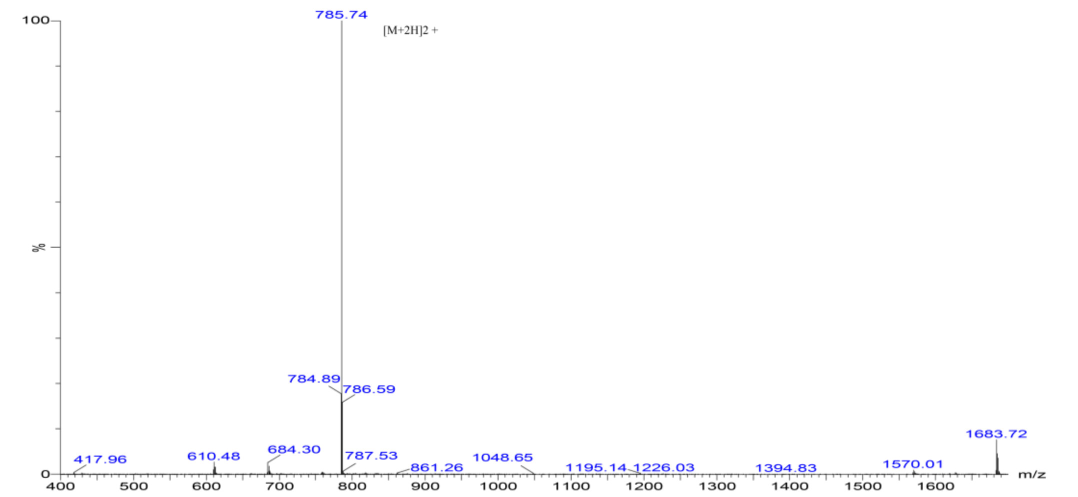

Description: Mobile phase:Buffer A:0.1%TFA in H2O
Buffer B:0.1%TFA in ACN
Gradient:34-44% B in20min , Flow rate:1ml/min
Column:Unitary C18,4.6*150mm,5um 100A

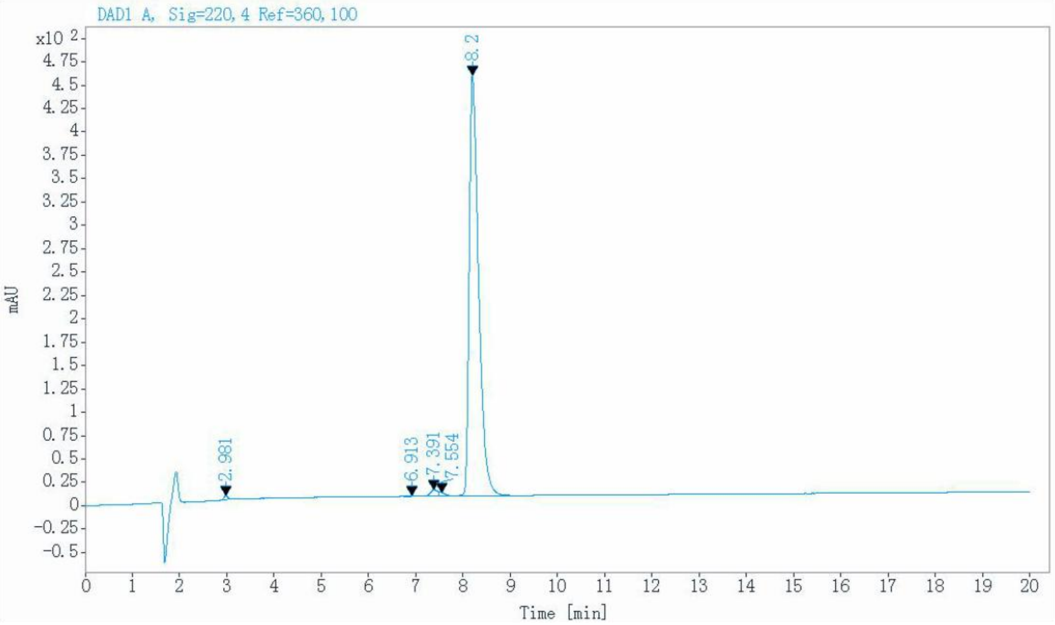

Signal: DAD1 A, Sig=220,4 Ref=360,100

RT[min]	Type	Width[min]	Area	Height	Area%	Name
2.981	BB	0.0981	27.0223	4.2524	0.4264	
6.913	VV B	0.1773	7.5686	0.6829	0.1194	
7.391	BV E	0.1515	68.9122	6.7270	1.0873	
7.554	VV E	0.1393	35.5743	3.7291	0.5613	
8.200	VV R	0.2086	6198.9824	450.3712	97.8057	
		Sum	6338.0599			

The LRMS and HPLC purity data of VTP-44

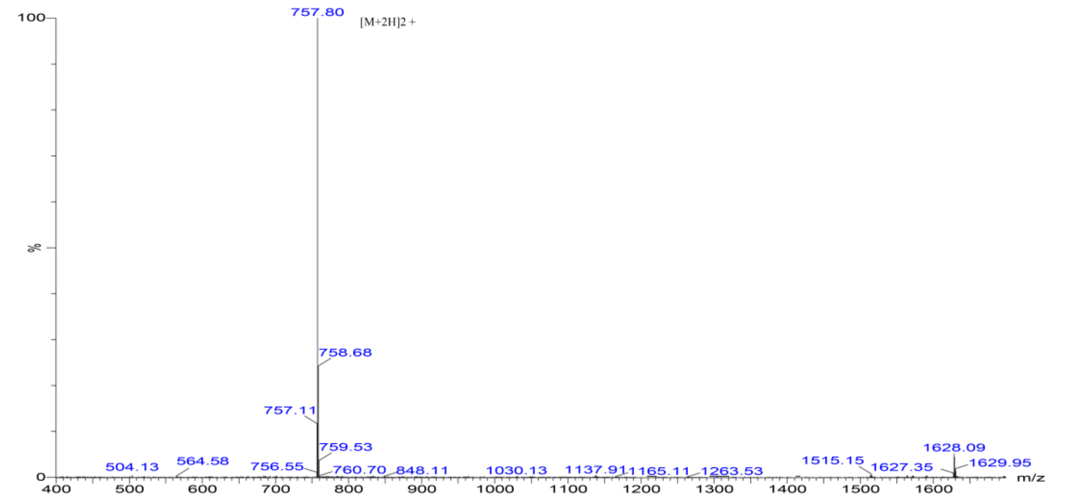

Description: Mobile phase:Buffer A:0.1%TFA in H2O
Buffer B:0.1%TFA in ACN
Gradient:34-54% B in20min , Flow rate:1ml/min
Column:Unitary C18,4.6*150mm,5um 100A

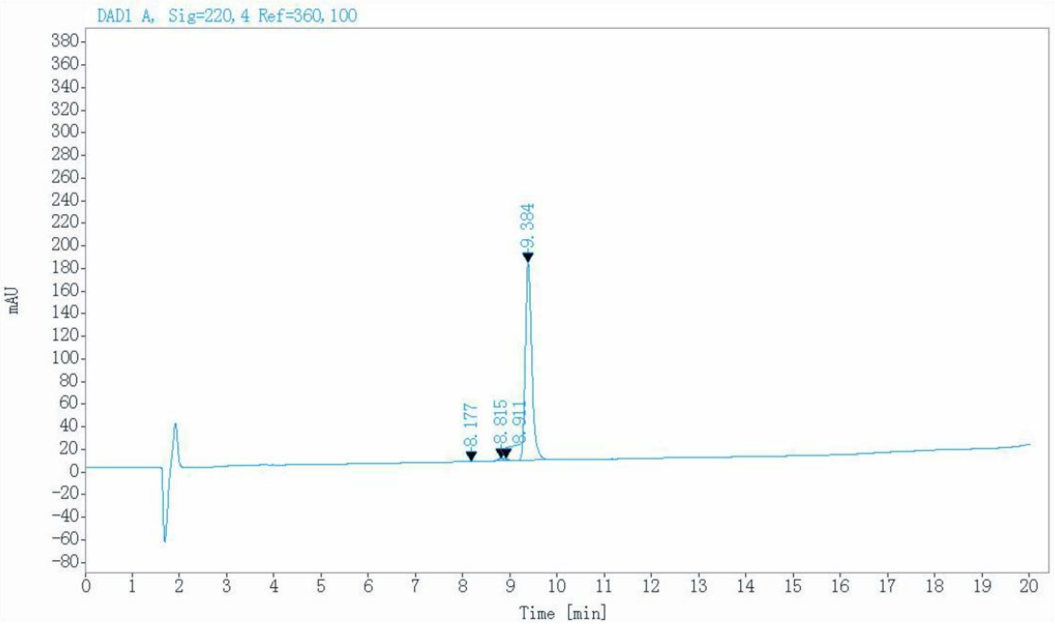

Signal: DAD1 A, Sig=220,4 Ref=360,100

RT[min]	Type	Width[min]	Area	Height	Area%	Name
8.177	MM	0.1155	2.3102	0.3333	0.1349	
8.815	VV	0.1240	15.0493	1.9955	0.8788	
8.911	VV B	0.1111	11.7842	1.5781	0.6881	
9.384	VBA	0.1482	1683.3337	175.0083	98.2981	
		Sum	1712.4776			

The LRMS and HPLC purity data of VTP-45

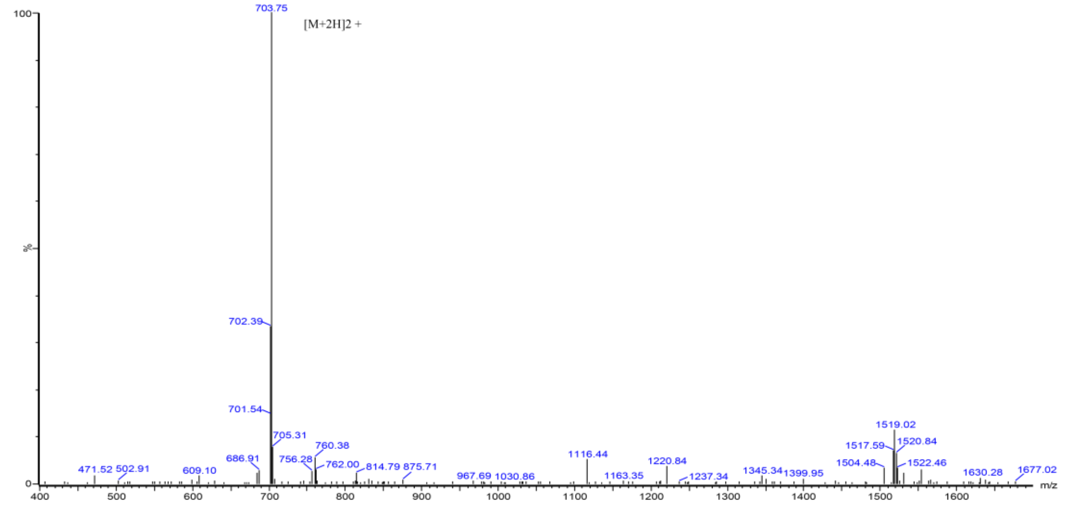

Description: Mobile phase:Buffer A:0.1%TFA in H2O
Buffer B:0.1%TFA in ACN
Gradient:30-50% B in20min , Flow rate:1ml/min
Column:Unitary C18, 4.6*150mm, 5um 100A

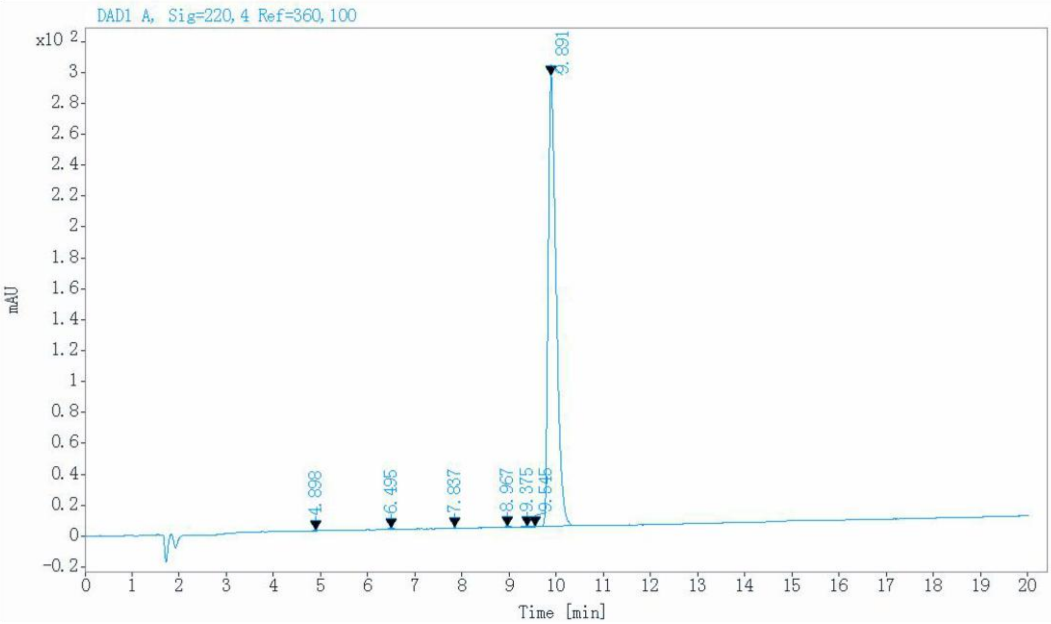

Signal: DAD1 A, Sig=220, 4 Ref=360, 100

RT[min]	Type	Width[min]	Area	Height	Area%	Name
4.898	MM	0.1239	3.6215	0.4873	0.1046	
6.495	BB	0.1280	7.3686	0.8954	0.2128	
7.837	MM	0.1435	3.6482	0.4238	0.1054	
8.967	MM	0.1268	2.9821	0.3920	0.0861	
9.375	MF	0.1672	4.4560	0.4441	0.1287	
9.545	FM	0.1547	4.4064	0.4748	0.1273	
9.891	FM	0.1965	3435.6621	291.4286	99.2351	
		Sum	3462.1449			

The LRMS and HPLC purity data of VTP-46

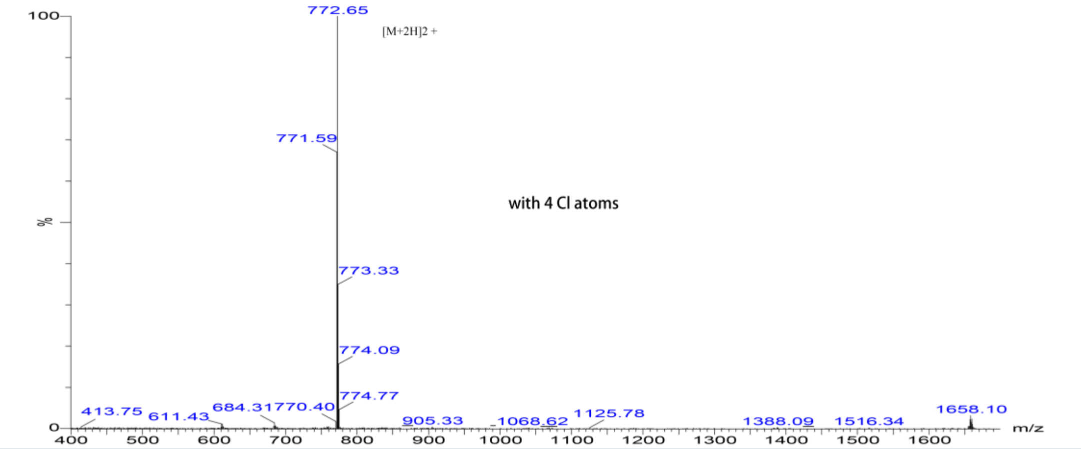

Description: Mobile phase:Buffer A:0.1%TFA in H2O
Buffer B:0.1%TFA in ACN
Gradient:38-48% B in20min , Flow rate:1ml/min
Column:Unitary C18, 4.6*150mm, 5um 100A

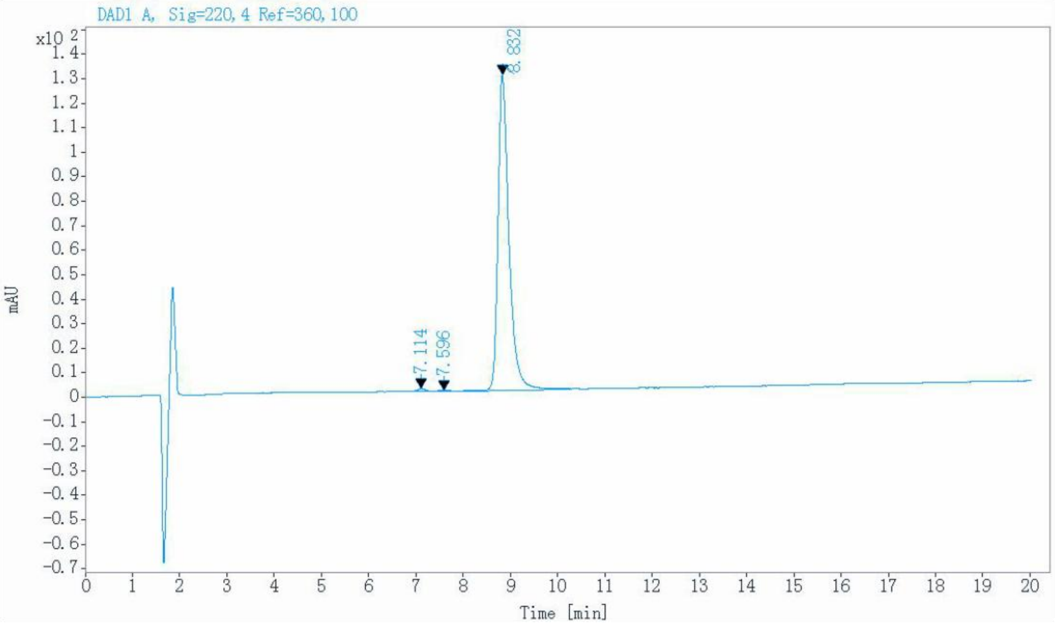

Signal: DAD1 A, Sig=220,4 Ref=360,100

RT[min]	Type	Width[min]	Area	Height	Area%	Name
7.114	BV	0.1859	13.9017	1.1427	0.6711	
7.596	VB	0.1892	4.5753	0.3312	0.2209	
8.832	BBA	0.2386	2053.0742	128.3594	99.1081	
		Sum	2071.5513			

The LRMS and HPLC purity data of VTP-47

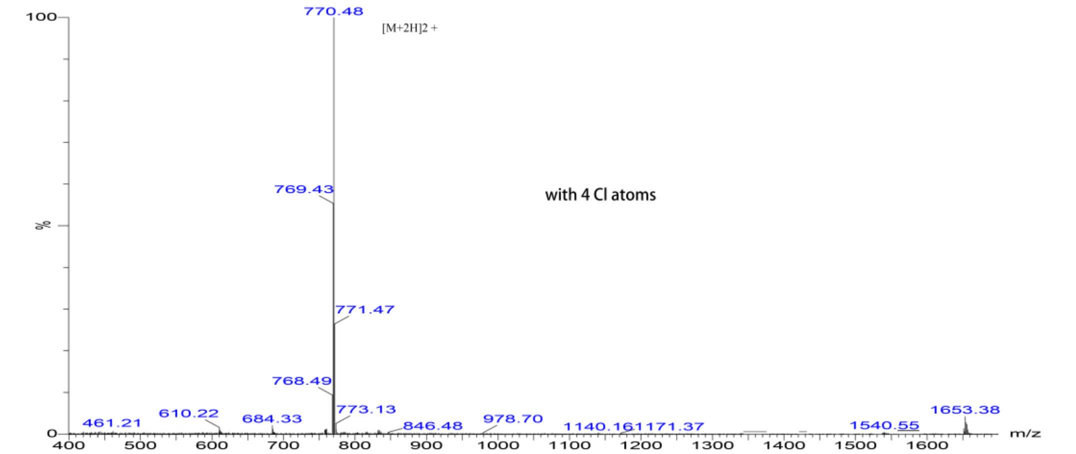

Description: Mobile phase:Buffer A:0.1%TFA in H2O
Buffer B:0.1%TFA in ACN
Gradient:40-50% B in 20min , Flow rate:1.0ml/min
Column:Unitary C18,4.6*150mm, 5um 100A

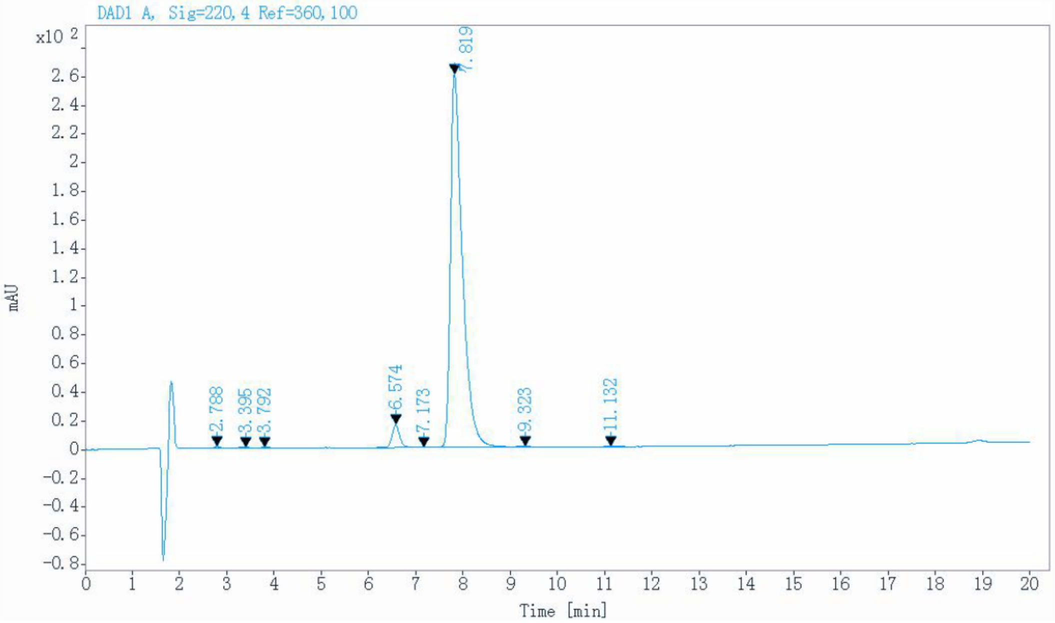

Signal: DAD1 A, Sig=220,4 Ref=360,100

RT[min]	Type	Width[min]	Area	Height	Area%	Name
2.788	BV	0.1454	13.4533	1.2924	0.2793	
3.395	VV	0.2047	11.7763	0.7954	0.2445	
3.792	VB	0.1630	7.5007	0.5795	0.1557	
6.574	MF	0.1860	178.3331	15.9830	3.7028	
7.173	FM	0.1901	4.1462	0.3635	0.0861	
7.819	BV R	0.2611	4579.4907	260.5300	95.0848	
9.323	VBAE	0.2575	13.2893	0.6420	0.2759	
11.132	BBA	0.2045	8.2266	0.5090	0.1708	
		Sum	4816.2162			

The LRMS and HPLC purity data of VTP-48

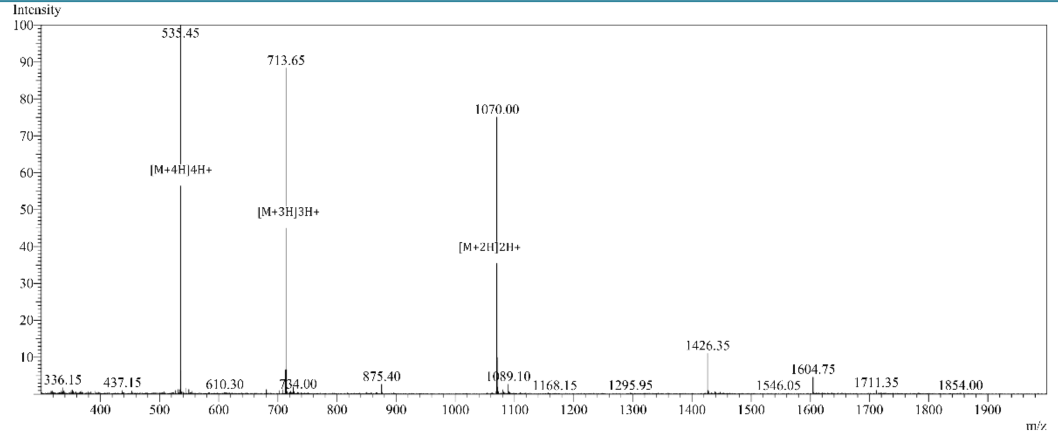

Pump A	:0.1%Trifluoroacetic in 100% water
Pump B	:0.1%Trifluoroacetic in 100% acetonitrile
Total Flow	:1ml/min
Wavelength	:220nm
Analytical column type	:SHIMADZU Inertsil ODS-SP(4.6*250mm*5um)
Dissolution method	:15%ACN+85%H2O
Inj. Volume	:8 uL

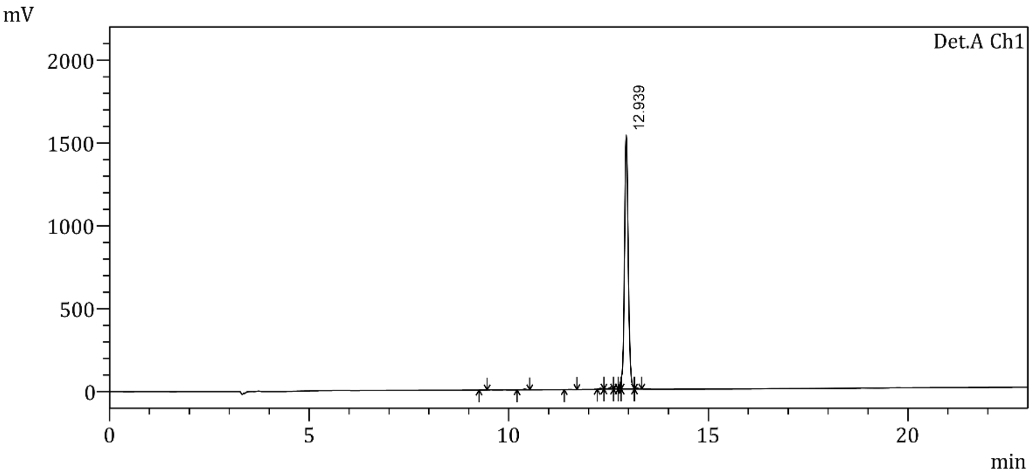

PeakTable

Detector A Ch1 220nm

Peak#	Ret. Time	Area	Height	Area %	Height %
1	9.356	11513	2076	0.110	0.128
2	10.399	26689	3638	0.254	0.224
3	11.530	6487	1290	0.062	0.079
4	12.296	21984	4450	0.209	0.274
5	12.569	77259	11234	0.735	0.692
6	12.733	79739	12647	0.759	0.779
7	12.808	139531	48973	1.328	3.017
8	12.939	10104618	1531576	96.152	94.364
9	13.186	41135	7166	0.391	0.442
Total		10508956	1623050	100.000	100.000

The LRMS and HPLC purity data of FITC-VTP-32

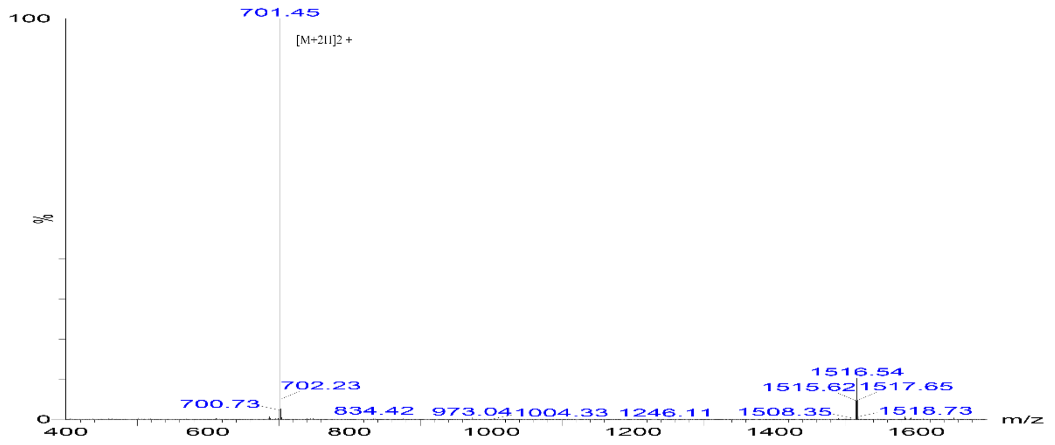

Description: Mobile Phase:Buffer A:0.1% TFA in H20
Buffer B: 0.1% TFA in ACN
Gradient:25-35%B in 20min, Flow rate:1.0ml/min
Column:Unitary C18 5um 100A 4.6*150mm
Instrument:Agilent HPLC 1100

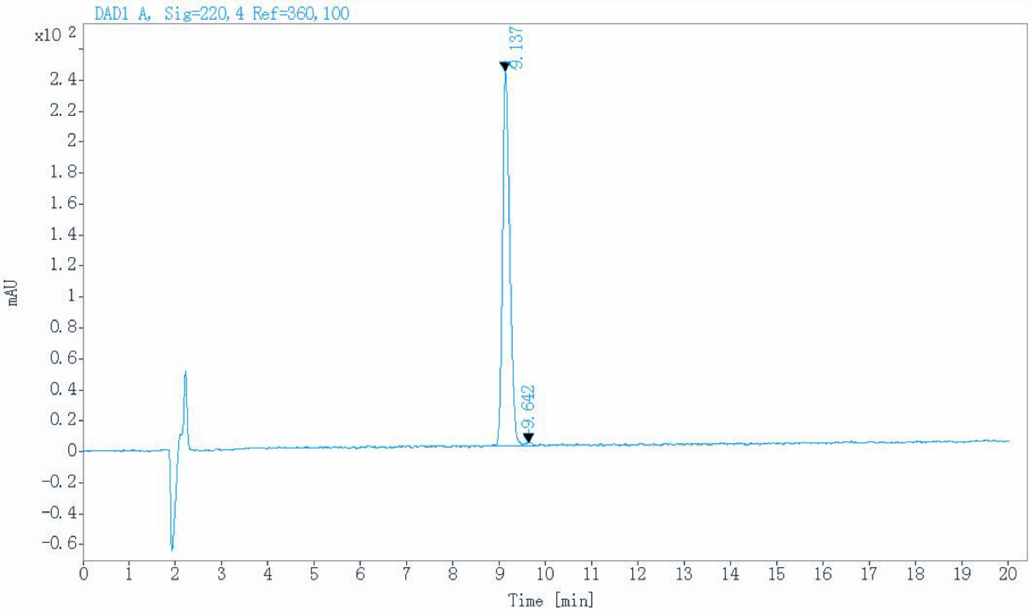

Signal: DAD1 A, Sig=220,4 Ref=360,100

RT[min]	Type	Width[min]	Area	Height	Area%	Name
9.137	MF	0.1814	2651.8301	243.5986	98.9633	
9.642	FM	0.2030	27.7791	2.2802	1.0367	
		Sum	2679.6091			

The LRMS and HPLC purity data of PAL-VTP-32
